# Characterizing heterologous protein burden in *Komagataella phaffii*

**DOI:** 10.1093/femsyr/foaf007

**Published:** 2025-02-19

**Authors:** Louise La Barbera Kastberg, Irene Hjorth Jacobsen, Emre Özdemir, Christopher T Workman, Michael Krogh Jensen, Jochen Förster

**Affiliations:** Department of Biotechnology and Biomedicine, Technical University of Denmark, Søltofts Plads Building 223, 2800 Kgs. Lyngby, Denmark; Novo Nordisk Foundation Center for Biosustainability, Technical University of Denmark, Kemitorvet Building 220, 2800 Kgs. Lyngby, Denmark; Department of Biotechnology and Biomedicine, Technical University of Denmark, Søltofts Plads Building 223, 2800 Kgs. Lyngby, Denmark; Novo Nordisk Foundation Center for Biosustainability, Technical University of Denmark, Kemitorvet Building 220, 2800 Kgs. Lyngby, Denmark; Department of Biotechnology and Biomedicine, Technical University of Denmark, Søltofts Plads Building 223, 2800 Kgs. Lyngby, Denmark; Novo Nordisk Foundation Center for Biosustainability, Technical University of Denmark, Kemitorvet Building 220, 2800 Kgs. Lyngby, Denmark; Novo Nordisk Foundation Center for Biosustainability, Technical University of Denmark, Kemitorvet Building 220, 2800 Kgs. Lyngby, Denmark

**Keywords:** heterologous protein production, burden, omics, continuous cultivation, *Komagataella phaffii*

## Abstract

Yeast is a widely utilized chassis for heterologous protein production, with *Komagataella phaffii* well-established as a prominent nonconventional yeast in this field. Despite its widespread recognition, there remains considerable potential to further optimize these cell factories to meet high production demands in a cost-effective and sustainable manner. Understanding the cellular response to the challenges of heterologous protein production can equip genetic engineers with crucial knowledge to develop enhanced strategies for constructing more efficient cell factories. In this study, we explore the molecular response of various *K. phaffii* strains that produce either the human insulin precursor or Mambalgin-1, examining changes in transcription and changes in intra- and extracellular protein levels. Our findings provide valuable insights into the molecular mechanisms that regulate the behaviour of *K. phaffii* production strains under the stress of producing different heterologous proteins. We believe that these results will serve as a foundation for identifying new genetic targets to improve strain robustness and productivity. In conclusion, we present new cellular and molecular insights into the response of *K. phaffii* cell factories to the challenges of burdensome heterologous protein production and our findings point to different engineering strategies for improved cell factory performance.

## Teaser

Cellular and molecular evidence is provided that production-limiting bottlenecks in *Komagataellaphaffii* cell factories, arising from burdensome heterologous protein secretion, are multifaceted, with respiration as the overarching factor.

## Introduction

The biomanufacturing industry is rapidly expanding, with an increasing share of proteins originating from genetically engineered organisms, particularly from microorganisms—microbial cell factories—and mammalian cell hosts (Rettenbacher et al. 2022). Engineering production strains requires careful consideration when selecting a host organism, as does the parts selection of, for example, the DNA construct (e.g. plasmid versus targeted integration site in host cell genome), promoter sequence to drive expression (e.g. constitutive or inducible), codon optimization, gene dosage, and a signal peptide (if desired). Yeasts are unicellular eukaryotes that grow and divide rapidly. They are relatively simple to genetically engineer and require much less expensive culturing conditions compared to mammalian cell lines or other higher eukaryotic protein expression systems. Importantly, unlike other microorganisms such as bacteria, yeast possess many of the posttranslational and secretion pathways present in higher eukaryotes, making these fungi particularly desirable for expressing a wide range of proteins from higher eukaryotic sources, such as human insulin. Indeed, most of the world’s insulin is produced from genetically engineered yeast cell factories (Kjeldsen et al. 2024).

Apart from the traditional yeast production host, *Saccharomyces cerevisiae*, a growing number of alternative or ‘nonconventional’ yeast species are already being used and explored for heterologous protein production optimization to overcome some limitations associated with *S. cerevisiae*. Among the nonconventional yeasts is *Komagataella phaffii* (formerly *Pichia pastoris*), representing one of the most utilized yeasts in the commercial production of heterologous proteins for pharmaceutical, industrial, or research applications. Some clear advantages of *K. phaffii* over *S. cerevisiae*, which can be exploited in certain fermentations, are: (1) *K. phaffii* can grow to much higher cell densities, (2) *K. phaffii* has a greater secretory capacity, and (3) *K. phaffii* secretes fewer proteins compared to other yeasts such as *S. cerevisiae*, facilitating less laborious downstream processes (Ata et al. 2021, Duman-Özdamar and Binay 2021).

However, a major limitation for biomanufacturing processes of heterologous protein in general, is that a living organism, such as yeast cell factories including *S. cerevisiae* or *K. phaffii*, is harnessed to produce a biomolecule that most often provides no benefit to the host cell. Furthermore, this process itself requires cellular resources such as metabolic precursors, redox cofactors, and energy sources, to be reallocated away from normal cellular metabolism towards the production of the desired biomolecule, and specifically resources for posttranslational processes involving protein folding, maturation, and secretion are expected to represent a burden-inducing bottleneck (Mattanovich et al. 2004, 2014). Burden is therefore imposed on the host cell metabolism and consequently can have a negative impact on cell growth, limit normal cellular metabolic processes, trigger stress or cell death, and even cause selective enrichment of low- or nonproducer cells during longer cultivations (Glick 1995, Heyland et al. 2011, Olsson et al. 2022). From the commercial perspective, burden is likely to cause a decrease in heterologous protein production over time in continuous fermentations as part of long-term adaptation (Kazemi Seresht et al. 2013a ). Thus, engineering strains that simply drive excessive expression of a heterologous protein might not actually lead to sustained high levels of production during the fermentation. Such strains might yield lower final product titres compared to strains driving less burdensome levels of expression (Cámara et al. 2016, He et al. 2020). Therefore, a better molecular understanding of the host cell response to production of heterologous proteins is crucial for the improvement of cell factories. Such knowledge could prove invaluable towards engineering more robust cell factories with enhanced product titres.

In recent years, significant progress has been made to understand the cellular and molecular biology of *K. phaffii* and further improve this powerful host for heterologous protein production. However, much of the reported application of *K. phaffii* as a host organism for heterologous protein production in publicly available literature takes advantage of its methylotrophic nature by engineering strains using the methanol-inducible alcohol oxidase gene (*AOX1*) derived promoter (p*_AOX1_*). As mentioned earlier, *K. phaffii* has many other advantages as host apart from its ability to assimilate methanol (indeed, methanol as a carbon source can be undesirable due to flammability), yet the genetic and molecular toolbox for this organism is limited. Large-scale system-level studies of this organism in different conditions are needed to add valuable molecular knowledge.

Studying burden response specifically in host cells driving heterologous protein production might unlock key targets and identify new strategies for genetically engineering cell factories that better tolerate/mitigate burden (Kastberg et al. 2022). Omics approaches provide powerful genome-wide insights into an organism at the molecular level for greater phenotypic understanding. Since flow of information in living organisms is not always unidirectional and does not always correlate linearly—for example RNA-guided gene regulation reverses the central dogma—combining multiple omics analyses can be helpful for linking the phenotype to the genotype (Amer and Baidoo 2021, Chang and Qi 2023). Overall, optimizing this process using information gained from these types of systems-level omics approaches will be essential for developing more sustainable and cost-effective production strains.

Here, we test varying levels of heterologous protein production in *K. phaffii* to assess the burden response at the level of the transcriptome, intracellular proteome, and secretome. We test the expression/secretion of two different heterologous proteins: human insulin precursor (hIP), a highly valuable pharmaceutical protein used to treat diabetes and commonly produced in yeast cell factories, and Mambalgin-1, a black mamba venom peptide with promising analgesic effect (Walsh 2005, Diochot et al. 2012, Kazemi Seresht et al. 2013a, Research Corporation Technologies 2024, Wright et al. 2020). We test the expression of these proteins in batch and continuous cultivations using glucose as carbon source, and measure the cellular impact under the control of the well-characterized promoter derived from *glyceraldehyde-3-phosphate* dehydrogenase gene (P*_GAP_*) (Çalik et al. 2015) in either one or six copies, as well as testing a novel promising powerful promoter derived from the *Stationary phase induced 1* gene (P*_SPI1_*), for the expansion of the *K. phaffii* genetic toolbox.

## Materials and methods

### Strains

**Figure 1.**
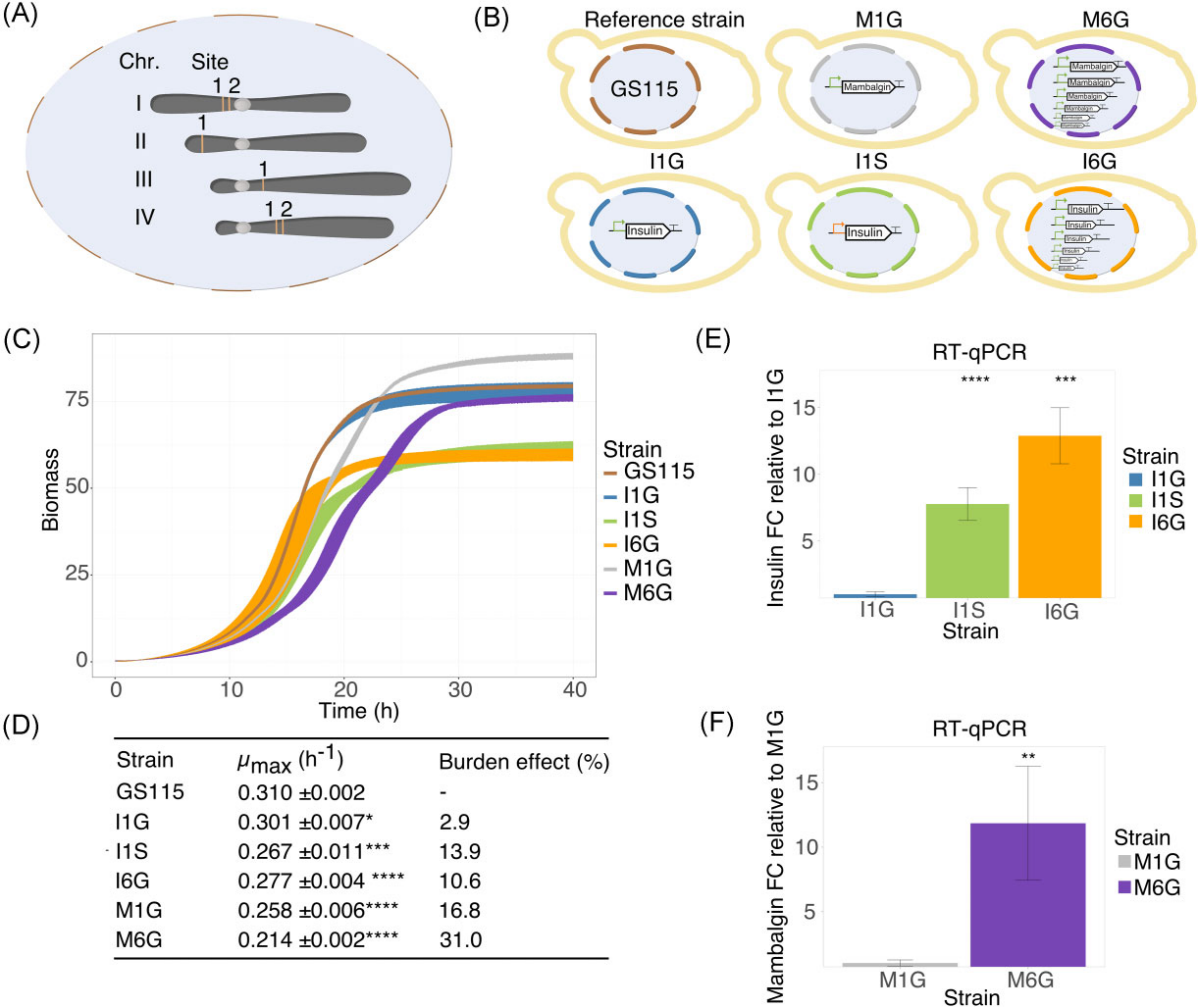
Production strain screening. (A) Location of genomic integration sites: ChrI-1, CI-2, CII-1, CIII-1, CIV-1, and CIV-2. (B) Production strains included in this study: reference strain GS115, Mambalgin-1 1 cassette with GAP promoter (M1G), M6G, Insulin 1 cassette SPI1 promoter (I1S), I1G, and I6G. (C) Growth profiles from parallelized scale-down batch mode cultivations in BioLector. Production strains were run in biological triplicates and technical duplicates. (D) Mean maximum growth rates (µ_max_) in exponential phase of each strain and the burden effect on growth as % drop in µ_max_ are listed. (E and F) RT-qPCR results from BioLector end-point samples for relative quantification of (E) insulin precursor and (F) Mambalgin-1 transcript levels in production strains. Statistical significance was assessed with *t*-test (*: *P* ≤ .05, **: *P* ≤ .01, ***: *P* ≤ .001, and ^****^: *P* ≤ .0001) against: (D) reference strain, (E) I1G, and (F) M1G. Abbreviations: Chr (chromosome), GAP (*glyceraldehyde-3-phosphate*), SPI1 (*stationary phase induced*), RT-qPCR (reverse transcription quantitative real-time PCR), and FC (fold change).

The commercial histidine auxotroph (*his4*) and haploid strain of *K. phaffii*, GS115 (Invitrogen), was used for this study. All strains used and engineered in this study are listed in Table 1, genomic sites for integration of expression cassettes are visualized in Fig. 1(A), and strains in Fig. 1(B).

**Table 1.** *Komagataella phaffii* strains. Abbreviations: Kp (*Komagataella phaffii*) and Sc (*Saccharomyces cerevisiae*).

Strain name	Genotype	Description	Reference
**GS115**	*his4*-	GS115 (reference strain) Kp.	Invitrogen
**Kp_ku70**	*his4*-, *ku70*∆::*UIDA*	GS115 with *ku70* knockout replaced with UidA cassette.	Kastberg et al. (2024)
**I1S (I1S-A, I1S-B, I1S-C)**	*his4-*, CI-1:: P*_SPI1_*-hIP-t*_AOX1_*	GS115 (*KU70* restored) expressing codon-optimized insulin precursor (EWK c-chain) under control of P*_SPI1_* and t*_AOX1_* with an Sc a-MF signal peptide and C-terminal his tag, integrated at CI-1.	This study. For integration site info see Kastberg et al. (2024)
**I1G (I1G-A, I1G-B, I1G-C)**	*his4-*, CI-1::P_*GAP*_-hIP-t_AOX1_	GS115 (*KU70* restored) expressing codon-optimized insulin precursor (EWK C-chain) under control of P*_GAP_* and t*_AOX1_* with an Sc MF-alpha signal peptide and C-terminal his tag, integrated at CI-1.	This study. For integration site info see Kastberg et al. (2024)
**I6G (I6G-A, I6G-B, I6G-C)**	*his4-*, CI-1::P*_GAP_*-hIP-t_*AOX1*_, CIII-1:: P*_GAP_*-hIP-t_*AOX1*_, CIV-1::P*_GAP_*-hIP-t_*AOX1*_, CII-1::P*_GAP_*-hIP-t_*AOX1*_, CIV-2::P*_GAP_*-hIP-t*_AOX1_*, CI-2::P*_GAP_*-hIP-t_*AOX1*_	GS115 (*KU70* restored) expressing codon-optimized insulin precursor (EWK C-chain) under control of P*_GAP_* and t*_AOX1_*with an Sc MF-alpha signal peptide and C-terminal his tag, integrated at CI-1, CI-2, CII-1, CIII-1, CIV-1, and CIV-2.	This study. For integration site info see Kastberg et al. (2024)
**M1G (M1G-A, M1G-B, M1G-C)**	*his4-*, CI-1::P*_GAP_*-Mam-t*_AOX1_*	GS115 (*KU70* restored) expressing codon-optimized Mambalgin-1 (Mam) under control of P*_GAP_* and t*_AOX1_*with an Sc aMF signal peptide and C-terminal his tag, integrated at CI-1.	This study. For integration site info see Kastberg et al. (2024)
**M6G (M6G-A, M6G-B, M6G-C)**	*his4-*, CI-1::P*_GAP_*-Mam-1-t*_AOX1_*, CIII-1:: P*_GAP_*-Mam-t*_AOX1_*, CIV-1::P*_GAP_*-Mam-t*_AOX1_*, CII-1::P*_GAP_*-Mam-t*_AOX1_*, CIV-2::P*_GAP_*-Mam-t*_AOX1_*, CI-2::P*_GAP_*-Mam-t*_AOX1_*	GS115 (*KU70* restored) expressing codon-optimized Mambalgin-1 (Mam) under control of P*_GAP_*and t*_AOX1_* with an Sc MF-alpha signal peptide and C-terminal his tag, integrated at CI-1, CI-2, CII-1, CIII-1, CIV-1, and CIV-2.	This study. For integration site info see Kastberg et al. (2024)

The competent *Escherichia coli* DH5α strain was used for propagating and storing cloned plasmids.

### Media

For initial screening, *K. phaffii* was grown in standard yeast peptone dextrose (YPD) medium containing 10 g/l yeast extract, 20 g/l peptone, and 20 g/l glucose. For solid YPD agar plates, 20 g/l agar was included. For selection of yeast with antibiotic resistance marker NatMX, medium was supplemented with 100 mg/l nourseothricin (ClonNat, Jena Bioscience). *K. phaffii* was incubated at 30°C and, for liquid cultures, in a MaxQ 8000 shaking incubator (Thermo Fisher Scientific) at 200 rpm. Defined growth medium (Baumann et al. 2008) was used for further characterization and chemostat cultivations. The composition of the defined growth medium was 10 or 20 g/l glucose, 0.9 g/l citric acid, 4.35 g/l (NH_4_)_2_HPO_4_, 0.01 g/l CaCL_2_•2H_2_O, 1,7 g/l KCl, 0.65 g/l MgSO_4_•7H_2_O, 0.48 g/l l-histidine, 1.6 ml/l *Pichia* trace metal 1 (PTM1), and 2 M HCl was used for pH adjustment to pH 5.0. PTM1 contained 5.99 g/l CuSO_4_•5H_2_O, 0.08 g/l NaI, 3.36 g/l MnSO_4_•H_2_O, 0.2 g/l Na_2_MoO_4_•2H_2_O, 0.02 g/l H_3_BO_3_, 0.5 g/l CoCl_2_•6H_2_O, 20.44 g/l ZnCl_2_, 63.95 g/l FeSO_4_•7H_2_O, 0.2 g/l biotin, and 5 ml/l H_2_SO_4_. For chemostat seed cultures, 500 ml shake flasks with 100 ml modified defined growth medium (Baumann et al. 2008) was used containing 20 g/l glucose, 1.8 g/l citric acid, 12.6 g/l (NH_4_)_2_HPO_4_, 0.02 g/l CaCL_2_•2H_2_O, 0.9 g/l KCl, 0.5 g/l MgSO_4_•7H_2_O, 0.48 g/l l-histidine, 4.6 ml/l PTM1, and 2 M HCl was used for pH adjustment to pH 5.0.

*Escherichia coli* was grown in Luria–Bertani broth supplemented with 100 mg/l ampicillin (Sigma) as liquid or solid (2% agar) medium.

### Plasmid and strain engineering

Uracil-Specific Excision Reagents (USER) cloning was used for plasmid construction (Nour-Eldin et al. 2006, 2010). Backbones were digested with restriction enzyme PacI or AsiSI (Thermo Fisher Scientific) and nicking enzyme Nb.BsmI or Nt.BbvCI (NEB), respectively. Cloning fragments were amplified with Phusion U Hot Start PCR Master Mix (Thermo Fisher Scientific) to allow integration of uracil in overhangs for USER cloning. Backbones and polymerase chain reaction (PCR) fragments were treated with USER enzyme (NEB) to get USER-compatible overhangs. Plasmids are listed in Table S1. All primers were ordered as oligos and purchased from Integrated DNA Technologies (IDT). USER-mix was transformed into competent *E. coli* DH5a by heat shock. Plasmids were isolated from *E. coli* using GenElute Plasmid Miniprep Kit (Sigma-Aldrich). Regions of interest were PCR amplified using Phusion High-Fidelity PCR Master Mix with HF Buffer (Thermo Fisher Scientific), and PCR products were column purified using illustra GFX PCR DNA and Gel Band Purification Kit (GE Healthcare) and validated with Sanger sequencing (Eurofins Genomics).

The DNA sequences encoding Mambalgin-1 and hIP, and the promoters and terminators controlling their expression were ordered as Gene Fragments from Twist Bioscience, fragments are listed in Table S2. The hIP protein is expressed as a single-chain protein comprising the A and B(1–29) chains of proinsulin and the C-chain of the human proinsulin was replaced by a small synthetic EWK sequence and a synthetic spacer peptide was added upstream of the B-chain for endoprotease processing (Kjeldsen et al. 2002, Kazemi Seresht et al. 2013b). The two heterologous proteins fused to a signal peptide were codon optimized using the free tool from GENEWIZ (Azenta Life Sciences) as this was found to improve two tested codon scores [number of effective codons (Nc) and tRNA adaptation index (tAI)] for the heterologous genes compared to codon optimization with IDT and Twist tools (Fig. S1).

For marker-free CRISPR/Cas9 mediated genomic integration in *K. phaffii*, we used a previously developed system (Strucko et al. 2024). We previously identified six new genomic integration sites, which we used for strain engineering in this study (Fig. 1A) (Kastberg et al. 2024). Transformation, plasmids containing repair templates were digested with NotI (NEB) restriction enzyme and column purified prior to transformation.

*Komagataella phaffii* was transformed by electroporation (Lin-Cereghino et al. 2005): cells from overnight cultures were resuspended and incubated in suspension buffer with 100 mM lithium acetate, 10 mM dithiothreitol, 0.6 M sorbitol, and 6 mM Tris–HCl pH 8.5. After three washes in 1 M sorbitol, cells were mixed with DNA and transferred to 0.2 cm electroporation cuvettes and pulsed with 2.0 kV for 6.10 ms in a MicroPulser Electroporator (BioRad). After incubation in YPD 1 M sorbitol, cells were streaked onto selective YPD plates. Transformants were subsequently replica plated onto nonselective plates for plasmid loss. Correct integration was verified by PCR with Phusion High-Fidelity PCR Master Mix with HF buffer (ThermoFisher Scientific) and Sanger sequencing (Eurofins, Germany). Prior to PCR and Sanger sequencing, genomic DNA was extracted by lithium acetate–SDS method (Lõoke et al. 2011). Regions of interest were PCR amplified and PCR products from single transformations were column purified using illustra^TM^ GFX PCR DNA and Gel Band Purification Kit (GE Healthcare).

### Microbioreactor cultivation

Microbioreactor batch fermentations were performed in 48-well FlowerPlates using BioLectorII (m2p-labs GmbH, Germany). Plates were covered with adhesive gas-permeable adhesive seals (Thermo Fisher Scientific). Noninvasive online measurements were performed detecting biomass in the form of scattered light intensity (LED module for biomass) and green fluorescence (LED module for GFP). Overnight cultures were diluted to OD_600_ 0.1 in a working volume of 1.5 ml in defined growth medium, and conditions were set to be controlled at 1000 rpm, 85% humidity, and 30°C. Data was analysed in R. End-point samples for RNA analysis were taken from the plates, snap-frozen in liquid nitrogen, and stored at −80°C.

### Chemostat cultivations

Continuous cultivations were carried out in fully controlled 1 l bioreactors (total volume of 1.6 l, max working volume 1 l) (Biostat Q plus, Sartorius, Germany) at 1 l working volume. Conditions were as follows: 25°C, airflow of 1 vvm, 800 rpm, and pH 5 (adjusted by 2 M H_2_SO_4_/2 M ammonia). Defined growth medium with 20 g/l glucose was used for batch, and the feed contained 10 g/l glucose. Off-gas from the process was analysed online by mass spectrometry (Thermo Scientific Prima PRO, UK), and data were collected continuously by MFCS/win software (Sartorius). After depletion of glucose in the batch medium, feeding was initiated at a dilution rate of 0.1/h. Sampling occurred at 6 and 9 residence times for the following purposes: OD_600_, cell dry weight (cDW), substrate and exometabolite (glucose, glycerol, acetate, ethanol, and succinat) HPLC, proteomics, secretomics, and transcriptomics.

OD was determined spectrophotometric at 600 nm (Shimadzu UV1800, Japan). cDW was determined by filtration using a 0.45 µM PES filter (Frisenette, Denmark), washing with ddH_2_O in approximately two times the sample volume, and subsequent drying in a microwave oven at 180 W for 20 min.

For substrate and exometabolite HPLC, samples were filtered using 0.45 µm filters (Qmax, Frisenette) and kept at −20°C until time of analysis. The analysis was run on an Ultimate 3000 UHPLC system (Thermo Scientific, Finland) in ion-exchange HPLC-mode with a BioRad Aminex HPX-87H column (Hercules, CA, USA) kept at 60°C. The injection volume was 20 µl, and the flow rate of 5 mM H_2_SO_4_ was 0.6 ml/min. Signals were detected by UV and RID, obtained data was treated using Chromeleon® Chromatography Data System (Thermo Fisher Scientific).

For omics studies, sampling into prechilled 50 ml Falcon tubes on ice, centrifuged at 4°C and 5000 × *g* for 5 min. Supernatant from proteomics samples was transferred to 15 ml prechilled Falcon tubes and the cell pellets were then washed once with phosphate buffered saline (PBS) buffer (1x Dulbecco’s, PanReac AppliChem, Germany). Pellets for transcriptomics were snap-frozen in liquid nitrogen and all sample types were stored at −80°C.

### Reverse transcription quantitative real-time PCR

Cell pellets were disrupted and homogenized by bead-milling in a TissueLyser (Qiagen), and total RNA extracted with RNeasy Plus Mini kit (Qiagen) according to manufacturer’s instructions. RNA was reverse transcribed into cDNA using iScript Select cDNA Synthesis Kit (BioRad). cDNA was further used as template for quantitative real-time PCR using Brilliant III Ultra-Fast SYBR Green QPCR Master Mix (Agilent) and run on a CFX Connect Real-Time PCR detection System (BioRad). For quantification of hIP the following primers were used: forward (fw) 5′-GTGGTTCTCATTTGGTTGAAGC-3′ and reverse (rv) 5′-ACAATACCTTTCCATTCTTTTGGAG-3′, and for Mambalgin: Fw 5′- AGCATGGGAAAGTAGTCACTTG-3′ and rv 5′-ACAAGAAGAAGAACAACCTTGC-3′, and for the reference gene ACT1: Fw 5′- GCTGAGCGTATGCAAAAGGAG-3′, rv 5′-ACCCAAAGAAGCGAGGATAGAAC-3′.

### Transcriptome analysis

#### RNA isolation, library preparation, and sequencing for transcriptomics

RNA was extracted by Eurofins RNA-seq service (Eurofins) and samples were sequenced on an Illumina platform NovaSeq 6000 S4 PE150 XP (Illumina, USA) by Eurofins RNA-seq service (Eurofins), generating 150 bp paired-end read strand-specific cDNA libraries. Poly(A)+ RNA was enriched from total RNA samples.

#### RNA-seq data processing and differential expression analysis

Raw reads were quality controlled with FastQC version 0.11.5 and reports were visualized and assessed with MultiQC version 1.14. Raw reads were quality trimmed from both ends and low-quality reads discarded using Trimmomatic version 0.39 settings ILLUMINACLIP: TruSeq3-PE-2.fa:2:30:10, LEADING:3, TRAILING:3, SLIDINGWINDOW:4:15, MINLEN:36. Unpaired reads were discarded, and paired read alignment and index building was carried out with HISAT2 version 2.2.1 using GS115 (reference strain) genome with GenBank accession number GCA_000027005.1 as reference (De Schutter et al. 2009). Insulin or Mambalgin-1 expression cassettes were added to the fasta and GTF file as an additional chromosome for alignment. StringTie version 2.2.1 was used for transcript assembly and quantification. For differential expression (DE) analysis edgeR R package was used and the limma R package for linear modelling.

### Proteome analysis

Proteome and secretome analyses were performed by Proteomics Core at DTU Bioengineering as described below.

#### Sample preparation

Cell pellets were resuspended in lysis buffer (6 M GdCl, 10 mM TCEP, 40 mM CAA, and 50 mM HEPES pH8.5) and subsequently boiled at 95°C for 5 min, after which they were sonicated on high for 5 × 30 s in a Bioruptor sonication water bath (Diagenode) at 4°C. Concentration of the samples were determined by BCA rapid gold (Thermo Scientific). Samples were diluted 1:3 with 10% acetonitrile (ACN) and 50 mM HEPES pH 8.5, and LysC (MS grade, Wako) was added in a 1:50 (enzyme to protein) ratio. Samples were incubated at 37°C for 4 h. Samples were further diluted to 1:10 with 10% ACN and 50 mM HEPES pH 8.5, and trypsin (MS grade, Promega) was added in a 1:100 (enzyme to protein) ratio and samples were incubated overnight at 37°C. Enzyme activity was quenched by adding 2% trifluoroacetic acid (TFA) to a final concentration of 1%. The eluted samples were concentrated in the speedvac for 30mins at 60°C before being acidified and desalted on SOLAµ SPE plate (HRP, Thermo Scientific), following the same procedure as previously described (Rappsilber et al. 2007). Dried peptides were reconstituted in 12 µl 2% ACN, 1%TFA.

#### Liquid chromatography tandem mass spectrometry

Peptides were loaded onto a 2 cm C18 trap column (ThermoFisher 164 946), connected in-line to a 15 cm C18 reverse-phase analytical column (Thermo EasySpray ES904) using 100% Buffer A (0.1% formic acid in water) at 750 bar, using the Thermo EasyLC 1200 HPLC system, and the column oven operating at 30°C. Peptides were eluted over a 70-min gradient ranging from 10% to 60% of 80% ACN, 0.1% formic acid at 250 nl/min, and the Orbitrap Exploris instrument (Thermo Fisher Scientific) was run in DDA mode with FAIMS Pro^TM^ Interface (ThermoFisher Scientific) cycling between CVs of −50 V and −70 V every 28 scans. Full MS spectra were collected at a resolution of 60 000, with an AGC target of 300% or maximum injection time set to ‘auto’ and a scan range of 375–1500 m/z. MS1 precursors with an intensity of > 5 × 10^3^ and charge state of 2–6 were selected for MS2 analysis. Dynamic exclusion was set to 60 s, the exclusion list was shared between CV values and Advanced Peak Determination was on. Precursors selected for MS2 were isolated in the quadrupole with a 1.6 m/z window. Ions were collected to a normalized AGC target set to 75% and maximum injection time was set to ‘auto’. Fragmentation was performed with a HCD normalized collision energy of 30% and MS2 spectra were acquired in the orbitrap at a resolution of 15 000. MS performance was verified for consistency by running complex cell lysate quality control standards, and chromatography was monitored to check for reproducibility.

#### Data analysis

The raw files were analysed using Proteome Discoverer 2.4. Label-free quantification was enabled in the processing and consensus steps, and spectra were matched against the Uniprot *K. phaffii* database and sequences of the heterologous proteins. Dynamic modifications were set as oxidation (M) and acetyl on protein N-termini. Cysteine carbamidomethyl was set as a static modification. All results were filtered to a 1% FDR, and protein quantification done using the built-in Minora Feature Detector. Data were further analysed in R. Protein abundance data were normalized with quantile normalization, and outliers were filtered out. The limma R package was used for DE analysis

#### Secretome analysis

Protein from supernatant samples were acetone precipitated overnight, the protein pellet resuspended in lysis buffer (6 M GdCl, 10 mM TCEP, 40 mM CAA, and 50 mM HEPES pH 8.5). Sample preparation for mass spectrometry followed the same procedure as for proteomics samples. 500 ng of each of peptide samples were loaded onto a 2 cm C18 trap column (ThermoFisher 164 705), connected in-line to a 15 cm C18 reverse-phase analytical column (Thermo EasySpray ES803) using 100% Buffer A (0.1% formic acid in water) at 750 bar, using the Thermo EasyLC 1200 HPLC system, and the column oven operating at 35°C. Peptides were eluted over a 140-min gradient ranging from 6% to 60% of 80% ACN, 0.1% formic acid at 250 nl/min, and the Q-Exactive instrument (Thermo Fisher Scientific) was run in a DD-MS2 top10 method. Full MS spectra were collected at a resolution of 70 000, with an AGC target of 3 × 10^6^ or maximum injection time of 20 ms and a scan range of 300–1750 m/z. The MS2 spectra were obtained at a resolution of 17 500, with an AGC target value of 1 × 10^6^ or maximum injection time of 60 ms, a normalized collision energy of 25 and an intensity threshold of 1.7e^4^. Dynamic exclusion was set to 60 s, and ions with a charge state < 2 or unknown were excluded. MS performance was verified for consistency by running complex cell lysate quality control standards, and chromatography was monitored to check for reproducibility.

Data analysis was following the same procedure as for proteome data. Signal-BLAST search (Frank and Sippl 2008) was conducted on protein sequences of differentially expressed secreted proteins to test if the proteins were predicted to contain a signal peptide.

### Functional profiling

Gene ontology (GO) enrichment analysis was performed using g:Profiler, searching for both GO terms and KEGG pathways enrichment (Raudvere et al. 2019) (https://biit.cs.ut.ee/gprofiler/gost). Default *P*-value correction method g:SCS was used.

## Results

### *Establishing K. phaffii* production strains to study burden response

For the characterization and comparison of different heterologous production strains in *K. phaffii* (Fig. 1B), we aimed to compare protein production and gene expression profiles in strains with different gene dosage (one and six copies) under the regulation of two different promoters, P*_GAP_* or P*_SPI1_*. P*_GAP_* is a widely used constitutive promoter and P*_SPI1_* is a less applied but promising constitutive promoter in *K. phaffii* (Waterham et al. 1997, Liang et al. 2013, Ata et al. 2017). We recently reidentified P*_SPI1_* through an *in silico* screen for strong promoters, prompting its further evaluation in this study (Kastberg et al. 2024). We therefore engineered production strains secreting either: (1) hIP or (2) Mambalgin-1. Using CRISPR-Cas9 technology, we stably integrated expression cassettes into each their integration site previously defined as suitable for strong expression in *K. phaffii* (Kastberg et al. 2024). The specific sites for integration are listed in Table 1 and visualized in Fig. 1(A). In total, we developed five production strains to test the burden response to heterologous protein production in *K. phaffii*: I1G (hIP, 1 cassette, P*_G__AP_*), I6G (hIP, 6 cassettes, P*_G__AP_*), I1S (hIP, 1 cassette P*_S__PI1_*), M1G (Mambalgin-1, 1 cassette, P*_G__AP_*), and M6G (Mambalgin-1, 6 cassettes, P*_G__AP_*) (Fig. 1B).

### *K. phaffii* production strain screening in parallelized batch mode microbioreactors

For the initial screening of production strains, we cultured the strains in defined growth medium in parallelized microbioreactors using the BioLector. For both, hIP and Mambalgin-1 production strains, we found that the maximum accumulated biomass (Tables S3 and S4) was lower for strains with six expression cassettes (M6G and I6G) compared to reference strain and strains with only one expression cassette under regulation of P*_GAP_* (M1G and I1G) (Fig. 1C). Similarly, the maximum growth rate (µ_max_) was lower for strains with six cassettes compared to reference strain as well as the two single-cassette strains, causing a larger percentage effect on growth (Fig. 1C and D). The growth profile of strains with a single hIP cassette under regulation of P*_SPI1_* (I1S) had similar growth profile as the strain with six hIP cassettes under regulation of P*_GAP_* (I6G) (Fig. 1C). Notably, the strain with a single Mambalgin-1 cassette (M1G) accumulated more biomass over time but exhibited significantly lower growth rate than reference strain (Fig. 1D; Tables S3 and S4). Meanwhile, the strain with a single hIP cassette under regulation of P*_GAP_* (I1G) grew like the reference strain and was not affected by production (Fig. 1C).

To directly measure differences in expression levels of hIP and Mambalgin-1 in these strains, we examined mRNA levels of the hIP and Mambalgin-1 genes in their respective strains by reverse transcription quantitative real-time PCR (RT-qPCR). Relative to I1G, the transcript levels increased ∼13-fold for I6G. For M6G, Mambalgin-1 mRNA levels increased by ∼12-fold relative to M1G. Overall, we found significantly greater relative mRNA expression for hIP and Mambalgin-1 in strains with six expression cassettes under regulation of P*_GAP_* (M6G and I6G) compared to strains with only one expression cassette under regulation of P*_GAP_* (M1G and I1G) in the microbioreactor batch mode cultivations performed here. We also note that mRNA transcript levels of hIP were also significantly higher by ∼8-fold in I1S relative to I1G.

### Validation of production strains in chemostat continuous cultivations

We next sought to apply the production strains under more industrially relevant bioprocess conditions to compare strain performance and acquire a system-level molecular understanding of the strains to couple genotype and phenotype. For this purpose, we carried out chemostat continuous cultivations for ∼90 h (dilution rate/growth rate at 0.1/h), preceded by an initial batch phase of ∼26 h (Fig. S2). During the chemostat we sampled twice: Time 1 (T1) at ∼60 h (8.7 generations/6 residence times) and time 2 (T2) at ∼90 h (13 generations/9 residence times) hours for physiology and omics analyses.

Chemostat cell density of the protein producing strains and the reference strain achieved steady-state (Fig. S2 and Table S5; CO_2_ yield) and carbon limitation (Table S5; glucose). A chemostat requires at least five residence times (volume changes) to achieve steady state without any disturbance (Bollmann et al. 2011). Thus, we sampled first time after six residence times (T1), confirmed by stable carbon evolution rate curves (Fig. S2). Only small levels of acetate and ethanol (<0.35 g) were detected (Table S5). No significant differences were detected for the variables listed in Table S5 between all strains and T1 and T2 between strains (except from one outlier M6G T1 versus M1G T2 acetate and ethanol) (Table S6).

### Transcriptional profiles of insulin precursor and Mambalgin-1

The transcriptomic response in *K. phaffii* to hIP and Mambalgin-1 production in continuous cultivation by RNA sequencing (RNA-seq) was investigated. Samples from the two time points T1 and T2 showed no significant difference and R-squared values of >0.95 in correlation analysis between T1 and T2 (Fig. S3), these were therefore treated as replicates to strengthen the statistical significance of the analysis.

**Figure 2.**
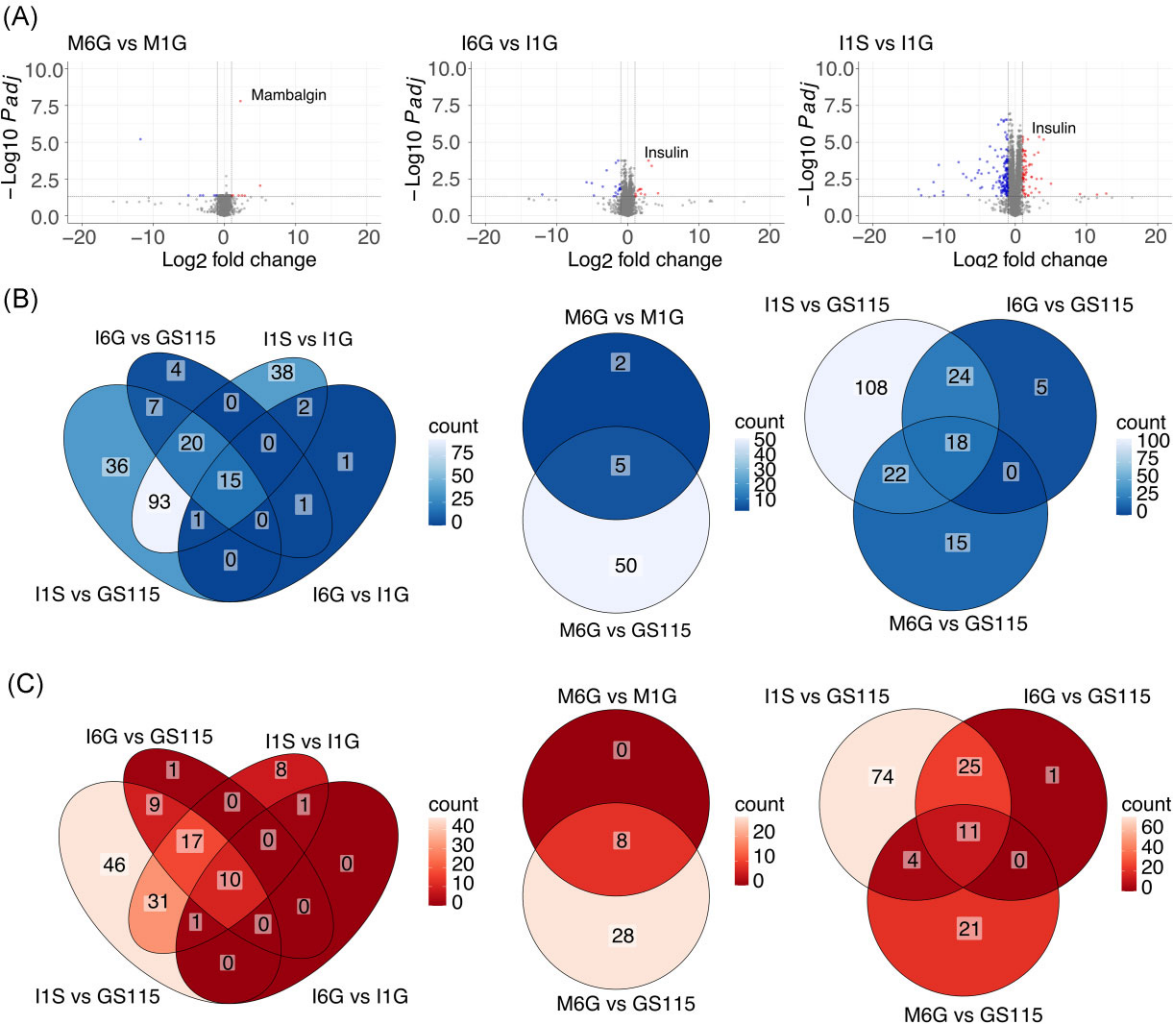
Transcriptome analysis of *K. phaffii* production strains in chemostat. (A) Volcano plots displaying DE genes for M6G relative to M1G and I6G or I1S relative to I1G. Each point represent a gene. Significantly downregulated genes are positioned to the left above the adjusted *P*-value cutoff line (blue), significantly upregulated genes are positioned to the right above the adjusted *P*-value cutoff (red), and genes that do not meet the significance threshold are located in the center or below the adjusted *P*-value cutoff line (grey) [transcript level, cutoff values (dashed lines) at |log_2_FC| >1 and adjusted *P*-value < .05]. Venn diagrams displaying number of DE (B) downregulated (log_2_FC <1 and adj. *P*-value < .05), and (C) upregulated (log_2_FC > 1 and adj. *P*-value < .05) genes and number of overlapping DE genes between all production strains and wild type. Abbreviations: DE (differential expressed) and FC (fold change).

With regard to the heterologous genes, DE analysis revealed that transcription levels of hIP were significantly (FDR < 0.05) and differentially (|log_2_FC|>1) expressed in both I1S and I6G relative to I1G strains, with a log_2_FC of 3.4 and 2.9, respectively (Fig. 2A; Tables S7 and S8). Compared to RT-qPCR analyses in batch cultivations, RNA-seq analyses reveal numerically higher hIP transcription levels in I1S compared to I6G. However, this difference was not statistically significant. Thus, six expression cassettes under regulation of P_*GAP*_ result in similar levels of the hIP transcript as a single expression cassette under regulation of P_*SPI1*_ under these conditions (Fig. S4). Transcription of Mambalgin-1 showed a 4.74 log_2_FC upregulation in M6G relative to M1G (Fig. 2A; Table S9).

### Transcriptomic response to various levels of Mambalgin-1 and hIP production

**Figure 3.**
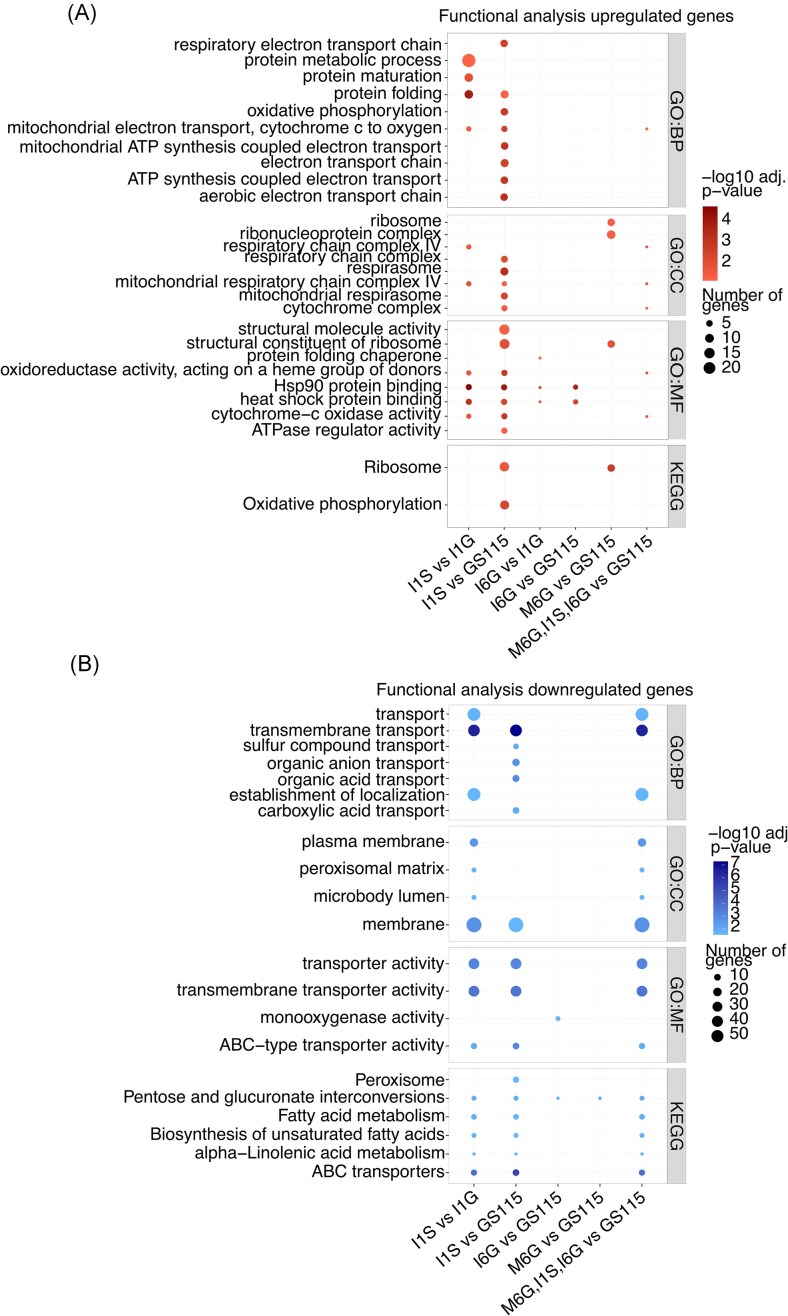
Functional profiling of RNA-seq DE genes. GO terms (BP, CC, and MF categories) and KEGG enrichment analysis of DE (A) upregulated and (B) downregulated genes for all strains. Abbreviations: DE (differentially expressed), GO (gene ontology), BP (biological process), CC (cellular component), MF (molecular function), and KEGG (Kyoto Encyclopaedia of Genes and Genomes).

Apart from changes in hIP and Mambalgin-1 expression, we found that more genes were DE (FDR < 0.05 and |log2FC|>1) in I1S than I6G relative to the low-producer I1G strain (237 genes for I1S and 32 genes for I6G; Tables S7 and S8) and reference strain (286 genes for I1S and 84 genes for I6G; Tables S10 and S11) (Fig. 2). The most DE genes were found in I1S (Fig. 2B and C). Among the upregulated genes in I1S, functional analysis revealed an enrichment of upregulated genes involved in respiration, protein folding, and heat shock protein binding. Conversely, genes mainly involved in transport were transcriptionally downregulated in I1S relative to I1G and reference strain (Fig. 3A and B). For I6G relative to reference strain or I1G, few GO terms were enriched. The upregulated genes were mainly related to heat shock protein binding (Fig. 3A). We found no DE genes in I1S relative to I6G (Fig. S4).

In Mambalgin-1 production strains, only Mambalgin-1 is differentially expressed in M1G relative to reference strain. A total of 91 genes are differentially expressed in M6G relative to reference strain and 15 genes in M6G relative to M1G (Fig. 2; Tables S9 and S12). Functional analysis suggests an upregulation of ribosomal genes such as 40S ribosomal protein subunit S29, *RPS29* (C4QYN4/PAS_chr1-4_0504), and 60S ribosomal protein subunit L23, *RPL23* (C4QVD0/PAS_chr1-3_0300), in M6G relative to the reference strain (Fig. 3).

Interestingly, among the 11 upregulated DE genes shared between I1S, I6G, and M6G relative to reference strain (Fig. 2C), GO term-enrichment analysis reveals increased respiration (Fig. 3A). For the shared 19 downregulated genes (Fig. 2B), GO terms related to transmembrane transport are enriched (Fig. 3B).

### Intracellular proteome response to various levels of Mambalgin-1 and hIP

**Figure 4.**
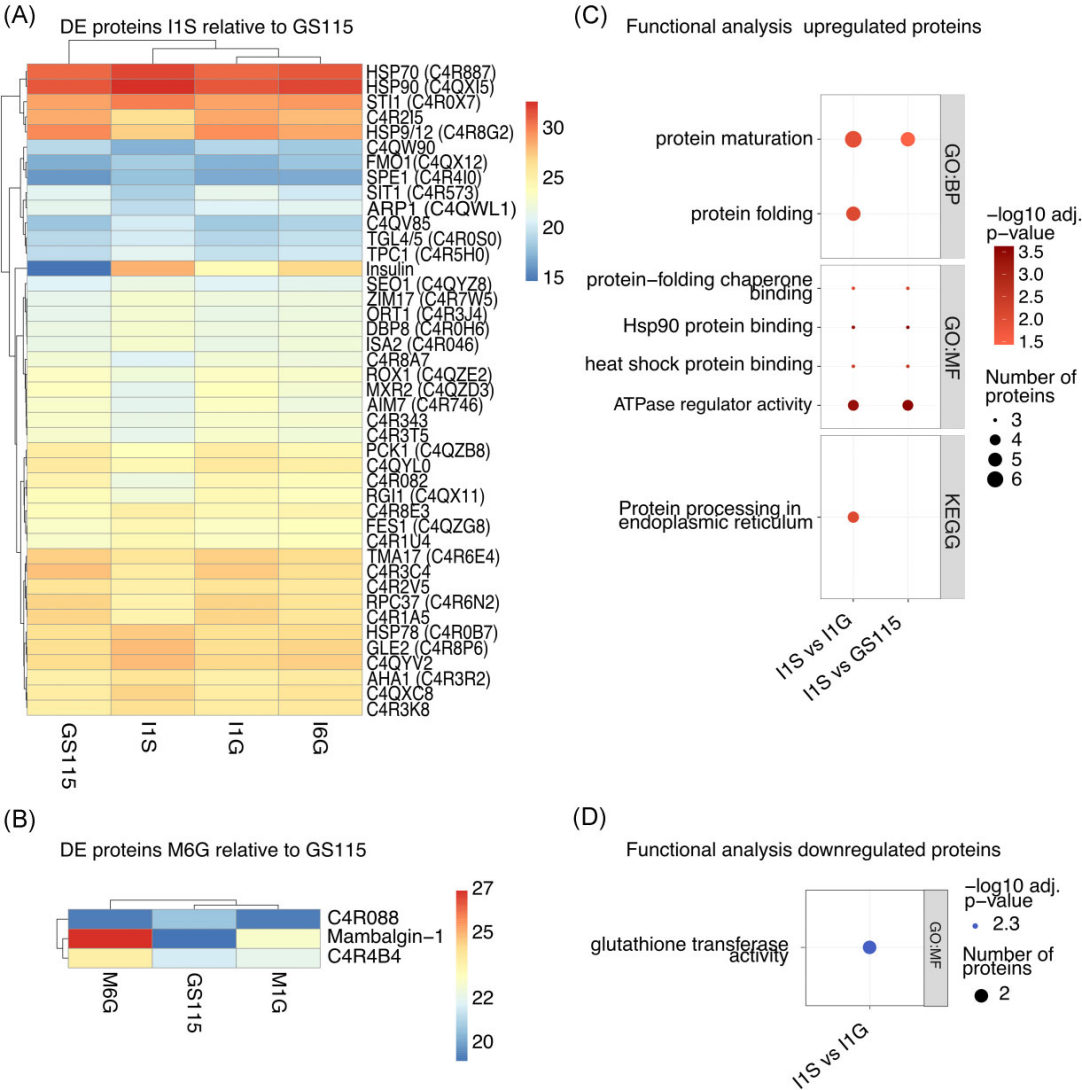
Proteome analysis of *K. phaffii* production strains in chemostat. DE analysis of proteome data. Heat map of DE proteins (adjusted *P*-value < .05, |log_2_FC| >1) listed with accession number and gene symbol if available in (A) I1S relative to reference strain and (B) M6G relative to reference strain (GS115), gradient representing the scaled log_2_ abundance. GO term-enrichment analysis in BP, CC, MF, and KEGG categories for DE (C) upregulated and (D) downregulated proteins. Data represents samples from biological triplicates and technical duplicates (T1 and T2). Abbreviations: DE (differential expression), reference strain (wild type), GO (gene ontology), BP (biological process), MF (molecular function), and KEGG (Kyoto Encyclopaedia of Genes and Genomes).

For the intracellular proteome analysis, sample T1 and T2 were also well-correlated and thus, joined to strengthen statistical power (Fig. S5). Intracellular proteome analysis of the production strains revealed significantly elevated levels of hIP in I1S relative to I1G (4.12 log_2_FC) (Fig. 4A). The intracellular hIP levels in I6G relative to I1G or I1S are not significant. In I6G relative to reference strain, hIP is the only DE. The proteome of I1S has, apart from significantly increased levels of the insulin precursor peptide, some differences relative to reference strain and I1G with 43 and 70 DE proteins, respectively (Table S13). Like RNA-seq described above, GO terms enriched for the upregulated intracellular proteins in I1S relative to I1G and reference strain centralizes around protein folding-related GO terms: ‘protein-folding chaperone binding’, ‘protein maturation’, ‘heat shock protein binding’, and ‘ATPase regulator’ (Fig. 4C). Meanwhile, the only GO term enriched among downregulated proteins in I1S relative to I1G is the molecular function ‘glutathione transferase activity’, encompassing the two proteins C4R0A6 and C4R2T6.

For Mambalgin-1 cell factories, intracellular levels of Mambalgin-1 are DE in M6G relative to the lower-producer strain M1G (4.15 log_2_FC), which is also the only DE protein in M6G relative to M1G. Only two proteins, apart from Mambalgin-1, are DE in M6G relative to reference strain (Fig. 4B; Table S14), which is the significantly upregulated C4R4B4 protein and significantly downregulated C4R088–both categorized as a hypothetical proteins with an unknown function (De Schutter et al. 2009).

### Secretome analysis reveals low heterologous protein secretion and few strain differences

**Figure 5.**
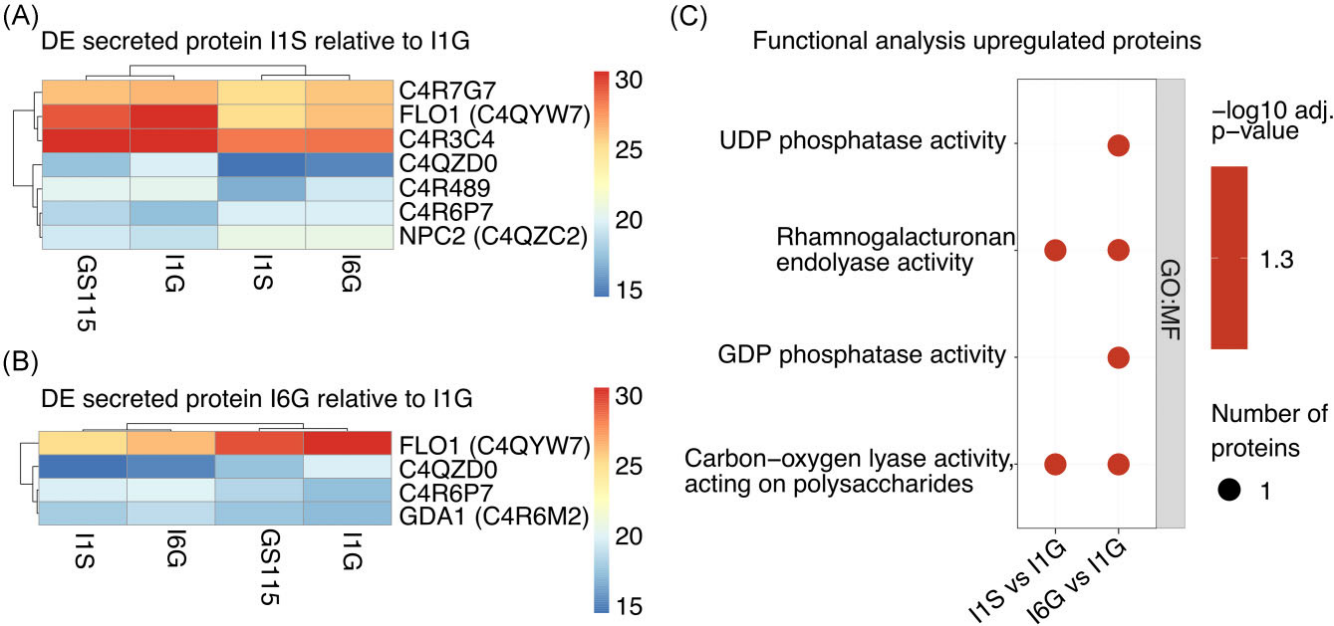
Secretome analysis of *K. phaffii* production strains in chemostat. DE analysis of secretome data: (A and B) heat map of differentially (adjusted *P*-value < .05, |log_2_FC| >1) secreted proteins listed with accession number and gene symbol if available in (A) I1S relative to I1G and (B) I6G relative to I1G, gradient representing the scaled log_2_ abundance. (C and D) GO term-enrichment analysis of differentially (C) upregulated secreted proteins in I1S and I6G strains relative to I1G. Data represent samples from biological triplicates and technical duplicates (T1 and T2). Abbreviations: DE (differential expression), GO (gene ontology), BP (biological process), MF (molecular function), and KEGG (Kyoto Encyclopedia of Genes and Genomes).

To complement and build on our analyses of the transcriptome and intracellular proteome of our production strains, we also examined the extracellular proteome (secretome) to assess the ability of our production strains to effectively secrete heterologous proteins. We were also interested in examining any potential alterations in the composition of the normal secretome of *K. phaffii* as a response to burdensome heterologous protein production. Here, the T1 and T2 biological triplicates were combined as well, to ensure consistency and more robust statistical power even though lower similarity is observed between samples which, however, also applies for the biological triplicates (Pearson correlation coefficient < 0.95 for some samples/replicates) (Fig. S6). Meanwhile, to exclude DE proteins likely present in the secretome due to cell lysis, we used Signal-BLAST to identify signal peptides in the protein sequences (Frank and Sippl 2008). Proteins lacking predicted signal peptides were excluded (Tables S15 and S16). In contrast to intracellular proteome analyses described above, we found that hIP was not significantly upregulated (cutoff: adjusted *P*-value < .05, |log_2_FC|>1) in the secretome of I1S nor I6G relative to I1G (Fig. 5A and B show hIP is absent from the list of DE proteins in the secretome of I1S and I6G relative to I1G, Table S15). Mambalgin-1, on the other hand, was significantly upregulated (1.8 log_2_FC) in secretome samples from M6G relative to M1G (Table S16), which however, is lower than the 4.15 Log_2_FC observed in the intracellular proteome.

Apart from DE of hIP and Mambalgin-1 proteins, we investigated the rest of the secretomes and assume only proteins with a predicted signal peptide as true hits. Relative to reference strain, only 1 secreted protein was DE in I1S, and none in neither I6G, I1G, M6G, or M1G (apart from the heterologous proteins) (Tables S15 and S16). When comparing high-producer strains (I6G, I1S, or M6G) to the low-producer strains (I1G or M1G), we found 7 DE secreted proteins in I1S (Fig. 5A) and 4 in I6G relative to I1G (Fig. 5B), and none in M6G relative to M1G. For these DE secreted proteins, few GO terms were enriched (Fig. 5C): For I1S and I6G relative to I1G GO terms were enriched for the upregulated proteins, encompassing GO terms related to e.g. phosphatase activity (Fig. 5C). No enrichment of GO terms was observed among the downregulated DE proteins in the secretomes.

### Overlap between omics datasets

**Figure 6.**
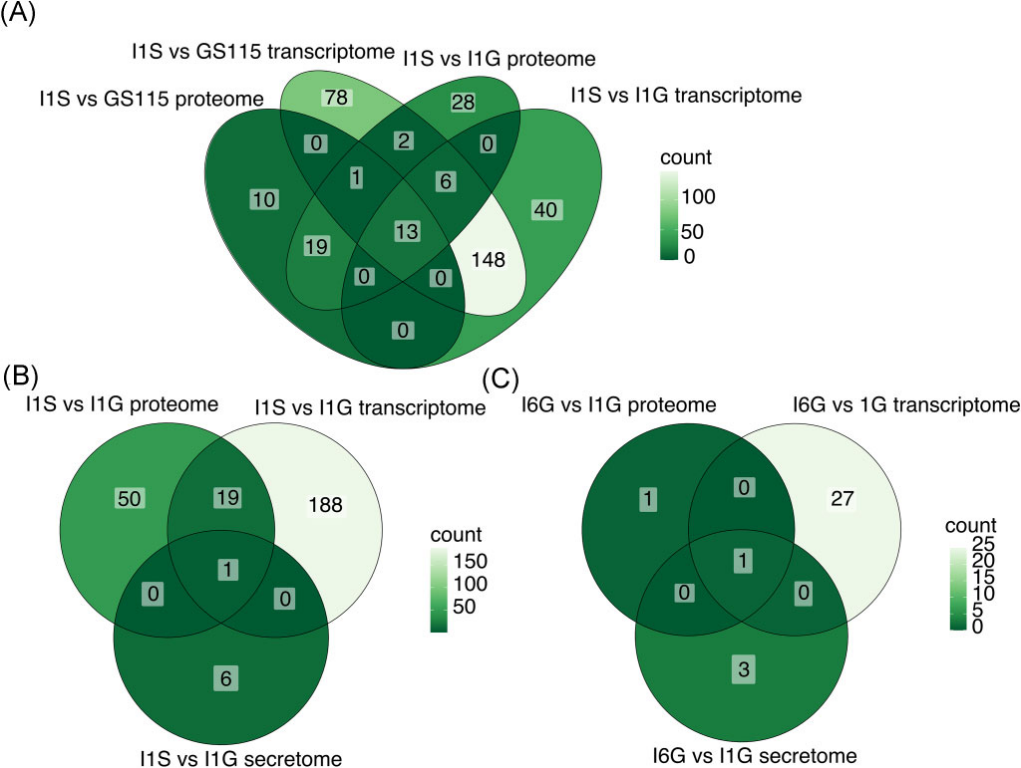
Overlap of DE genes and proteins between transcriptome, intracellular proteome and secretome. Venn diagrams showing number of overlapping DE genes/proteins for (A) I1S relative to I1G and GS115 (reference strain) in transcriptome and proteome, (B) I1S relative to I1G in transcriptome, proteome, and secretome, and (C) I6G relative to I1G in transcriptome, proteome, and secretome. Abbreviations: DE (differentially expressed).

We next investigated the overlap between DE transcripts and proteins. For I1S relative to reference strain and I1G we found an overlap of 13 genes/intracellular proteins (Fig. 6A). GO term-enrichment analysis once again indicate protein folding represents a bottleneck in I1S with the following GO terms being enriched for upregulated genes/proteins: ‘Hsp90/heat shock protein binding’, ‘protein-folding chaperone binding’, ‘ATPase regulator activity’, ‘protein folding chaperone’, ‘protein folding’, and ‘protein maturation’. No GO terms were enriched for the downregulated genes/proteins: C4QZB8 (PAS_FragB_0061) a phosphoenolpyruvate carboxykinase; C4R2I5 (PAS_chr2-2_0208) a hypothetical protein; C4R573 (PAS_chr3_0662) a ferrioxamine B transporter; C4R8G2 (PAS_chr4_0627) plasma membrane protein protecting against desiccation; C4R343 (PAS_chr2-2_0019) peroxisomal 2,4-dienoyl-CoA reductase; C4QZE2 (PAS_chr2-1_0017) a haem dependent repressor of hypoxic genes, and *Regulation by Oxygen* (*ROX1*/Rox1p) ortholog.

When including secretome results for I1S relative to I1G, 1 gene/intracellular protein/extracellular protein is shared between all three omics datasets (Fig. 6B). The overlapping gene/protein is the downregulated C4QYW7 (PAS_chr1-4_0584), a lectin-like protein similar to the *S. cerevisiae* flocculation 1 (Flo1p) protein. The Flo1p ortholog is also the only shared DE gene/protein between the three omics datasets of I6G relative to I1G (Fig. 6C). For M6G, we found no shared transcripts/proteins (apart from Mambalgin-1) between the three datasets.

## Discussion

In this study, we conducted batch and glucose-limited continuous cultivations using engineered *K. phaffii* designed to produce heterologous proteins targeted for secretion, specifically hIP and Mambalgin-1. These proteins were expressed with varying gene dosage under the control of the constitutive benchmark promoter P*_GAP_*, with hIP also expressed using the novel promoter P*_SPI1_*. Our goal was to characterize the burden associated with enhanced protein expression, achieved by modulating promoter selection and/or gene copy number. By examining the physiological and molecular response of five different production strains (I1G, I1S, I6G, M1G, and M6G), in comparison to the reference strain (GS115), our findings provide valuable insight for bioengineers. These insights will help in assessing the impact of unnatural heterologous protein production on *K. phaffii* cell factories, ultimately guiding the development of more efficient and burden-resistant strains for future biotechnological application.

### High-producers show impaired growth in batch cultivations

During initial screening in batch cultivations, we observed reduced biomass accumulation and µ_max_ for all three high-producer strains (M6G, I6G, and I1S) compared to the reference strain (GS115) and the two low-producer strains (M1G and I1G). We conclude that increasing heterologous protein expression impairs growth and cell physiology in batch mode cultivations and attribute this behaviour to reallocation of cellular resources away from biomass formation and towards heterologous protein production consistent with findings reported previously (Cámara et al. 2016, Ceroni et al. 2018, Gorczyca et al. 2022).

### RNA-seq reveals power demand and protein-specific transcriptomic responses

We subsequently performed carbon-limited continuous cultivations in chemostats. The chemostat mode provides a balanced system with all cells assumed to be in the same metabolic state, allowing for direct comparisons of omics samples (Kazemi Seresht et al. 2013a). Our chemostat experiments showed that cells reached a steady state (Fig. S2) and evidenced that there is little difference between biomass and CO_2_ yield at two sampling points (T1 and T2) during the feed phase (Table S5).

Further characterization of strains in chemostats unravelled the impact of increased gene dosage, different heterologous proteins and various promoters on cell factories by characterizing the strains at the transcriptomic, proteomic, and secretomic levels.

Functional profiling of transcriptome data revealed that DE genes shared among the high producer strains M6G, I6G, and I1S were enriched in respiration-related GO terms among the upregulated genes while genes involved in transport were down regulated (Fig. 3). This suggests a common transcriptomic response in the high-producer strains, characterized by need for increased power generation in the mitochondria at the expense of resources for transport, which may contribute to loss of secretory processing of heterologous proteins.

Besides, functional analysis of DE genes for each individual high-producer strain compared to the reference strain and/or low-producer strains, indicates distinct cellular responses depending on whether the strains produce hIP or Mambalgin-1. In the high hIP-producing strains, I1S and I6G, functional analysis revealed a transcriptional upregulation of heat shock protein-binding proteins, with I1S also showing upregulation of respiration-associated genes. For the M6G strain, ribosomal genes were generally upregulated. These findings suggest that while respiration/ATP limitations are a common trend among all three high-producer strains, translation of the Mambalgin-1 protein at high levels may pose additional challenges at the translational level. A potential increased demand on ribosomes suggests that the cell reallocates more resources to the translational machinery when expressing Mambalgin-1. In contrast for hIP, the primary challenges appear to happen posttranslationally, with a significant folding burden when expressed at high levels. These different needs between strains dealing with high heterologous protein production is possibly due to different biochemical challenges associated with translation, folding, and export of the different proteins, as previously reported in *Yarrowia lipolytica* (Korpys-Woźniak and Celińska 2021).

### Limited omics overlap and resource allocation insights

Contrary to what we observed in the transcriptomes, we found very few or no DE proteins in the proteomes and secretomes of I6G and M6G compared to the reference strain and low-producer strains. So the load on the cell stemming from high heterologous production is not reflected in the intracellular or extracellular proteome in these strains as observed in the transcriptomes. It is common to see no/poor proportional relationship between mRNA and protein expression in omics profiles (Haider and Pal 2013). For example, in order for mRNA to be translated, many physical properties will have an impact on the efficiency such as codon bias, ribosomal occupancy time, and other posttranscriptional regulatory functions (Haider and Pal 2013). Thus, it is not surprising that we observe different regulatory behaviour when looking at the transcriptomic and proteomics profiles. However, despite a poor direct overlap of features between the profiles (Fig. 6), we do see a level of overlap from the functional analyses of these datasets in the I1S strain. The differences in the intracellular proteome of I1S relative to I1G and the reference strain reflect the results observed in the transcriptomes when looking at enriched GO terms for upregulated proteins alone: more resources are allocated to protein folding and maturation.

Among downregulated intracellular proteins in I1S, we observe an enrichment of proteins related to ‘glutathione transferase activity’ (C4R0A6 and C4R2T6). A possible explanation for downregulation of such proteins could be the cell’s attempt to prevent apoptosis. For example, C4R2T6 is a glutathione transferase capable of glutathionylating proteins. The *S. cerevisiae* ortholog glutathione transferase (Gtt1p) was previously demonstrated to mediate activation of calcium channels, leading to apoptosis in response to changes in the intracellular redox state (Chandel et al. 2016). Interestingly, in a comparative proteomics study of *K. phaffii* cultivated in glucose or methanol, C4R2T6 (Gtt1p ortholog) was significantly upregulated in methanol compared to glucose cultivations (Hou et al. 2022). Therefore, we speculate that the downregulation of these proteins in I1S may be due to resource reallocation, meaning the host cell is downregulating proteins that are not as essential under the given growth conditions, similar to what was previously reported in *Y. lipolytica* overproducing various types of heterologous proteins (Korpys-Woźniak and Celińska 2021).

Meanwhile, we might not see all functional patterns in the functional analysis, as many proteins in *K. phaffii* still remain uncharacterized. For example, out of the seven DE proteins in the I1S relative to I1G secretome, four proteins are described as hypothetical protein of unknown function. This highlights a substantial challenge related to working with more novel host cell organisms compared to the conventional budding yeast *S. cerevisiae*. Beyond functional analysis, tools like BLAST (Camacho et al. 2009) can provide deeper insights into molecular responses than broader analyses, such as functional analysis with limitations as described above, deducing the function of single uncharacterized proteins and also provide more detail on characterized proteins than the enrichment of related GO terms for characterized differentially expressed features.

Despite the limited overlap between the different omics profiles, certain feature findings should be highlighted. A notable example is the downregulation of the *FLO1*/Flo1p *S. cerevisiae* ortholog, observed in both I1S and I6G compared to the reference strain and/or I1G in all three omics datasets. Flo1p is a protein involved in flocculation, the process by which yeast cells aggregate and precipitate during fermentation (Smukalla et al. 2008). In contrast, in previous studies on flocculating strains of *S. cerevisiae*, which exhibited upregulation of *FLO* genes, revealed an upregulation of transporters and downregulation of genes involved in mitosis and ribosomal functions (Smukalla et al. 2008). This is the opposite of what we observe in our hIP strains, where *FLO1*/Flo1p is downregulated suggesting a reallocation of resources from transport processes to protein folding. Although flocculation mechanisms are not yet well understood in *K. phaffii*, previous research demonstrated that knocking out genes related to morphological differentiation resulted in higher heterologous protein secretion in *K. phaffii* (Gasser et al. 2020, Ata et al. 2021). This response is similar to the burden observed in the high-producer insulin strains, I1S and I6G, compared to the lower producer strain I1G. Morphologies such as pseudohyphal or filamentous growth result in higher surface-to-volume ratio, which demands a higher supply of membrane and cell wall components (Tyo et al. 2012), and might in turn also open more surface for secretion. Meanwhile, changes in morphology toward ovoid shapes in *Y. lipolytica* production strains have been reported as a potential strategy to withstand cellular burden (Korpys-Woźniak and Celińska 2021). We speculate that the downregulation of *FLO1/Flo1p* is a strategy to conserve resources, thereby enhancing the cell’s ability to manage the burdensome demands from heterologous protein production. Future studies could investigate the morphotypes of *K. phaffii* production strains to better understand this molecular response to production and its impact on productivity.

### Genetic factors impacting the different responses

In addition to the shared increased demand for respiration-related genes observed in the transcriptomic profiles in high-producer strains, the different cellular responses to the two heterologous proteins may be attributed to distinct biochemical challenges associated with their translation, folding, and export of the two proteins. For instance, the amino acid composition of the heterologous protein might have an impact on its production. If the heterologous protein amino acid composition differs from the average amino acid composition of the host cell proteome, it may alter the global free amino acid pool or affect the cotranslational folding of the nascent polypeptide. First, the host cell may be limited by the amino acid metabolism, which can negatively affect production (Rußmayer et al. 2023). Next, the codon usage of a gene influences the timing of translational pausing, which is crucial for proper protein folding (Heyland et al. 2011, Kazemi Seresht et al. 2013a, Liao et al. 2019). Due to differences in codon preferences and amino acid availability in organisms, the codon usage pattern in heterologous genes often does not align with that of the native host, leading to improperly timed ribosome stalling as a result of amino acid depletion (Liao et al. 2019, Yip and Shao 2021). It has been demonstrated that slowing down translation elongation such as by deleting redundant ribosomal proteins, can improve cotranslational folding and increase the final production of heterologous protein in *K. phaffii* (Liao et al. 2019). This contrasts with the strategy observed in the M6G strain, where there is a transcriptional upregulation in ribosomal proteins. We expected that the translation of Mambalgin-1 would be challenging for *K. phaffii* due to its suboptimal Number of Effective Codons (Nc) and tRNA Adaptation Index scores (tAI), even after codon optimization (Fig. S1). Additionally, Mambalgin-1 belongs to the three-finger toxin family of venom proteins, known for their complex and difficult-to-translate folds that can cause ribosomal stalling (Kini and Doley 2010). This may explain the upregulation of ribosomal genes observed in the transcriptome and suggests that the primary bottleneck in high-producer strains expressing Mambalgin-1 is at the translational level. However, this translational bottleneck may be less detrimental than posttranslational bottlenecks as observed in hIP strains, given that we found significantly higher Mambalgin-1 levels in the M6G secretome compared to M1G, whereas no such significant difference was observed for I1S or I6G compared to I1G in the secretome.

Posttranslationally, protein modifications such as disulfide bonds are also likely to affect the cellular response (Hohenblum et al. 2004). Both Mambalgin-1 and hIP are stabilized by disulphide bonds: four in Mambalgin-1 and three in hIP (two inter A- and B-chain and one intra A-chain). The enrichment of GO terms related to protein folding and maturation for upregulated proteins in high-producer hIP strains (I1S and I6G) suggests that protein folding and/or disulfide bond formation are bottlenecks in these hIP strains. Secretory stress in yeast production strains can be due to overload of ER folding capacity, which can cause increased consumption of reducing equivalents (such as NADPH) if for instance protein folding is too slow to keep up with disulfide bond formation rates (Tyo et al. 2012). Such cellular load can lead to the production of more reactive oxygen species and elevated protein degradation (Tyo et al. 2012). However, we do not detect severe oxidative stress problems in the hIP producing strains. For example, C4QZE2 (*ROX1*/Rox1p ortholog), and the two proteins with glutathione transferase activity, C4R0A6 and C4R2T6, that would be expected to be upregulated during oxidative stress, are significantly downregulated. Additionally, our functional analysis did not reveal any enrichment in oxidative stress response pathways. Given that hIP protein is reported to fold relatively easily (Tyo et al. 2012), the main rate-limiting step in hIP production resides elsewhere.

The downregulation of *ROX1*/Rox1p in the I1S strains (the overlapping hit in I1S transcriptome and intracellular proteome relative to I1G strain and the reference strain, *ROX1*/Rox1p ortholog C4QZE2) could be a strategy for the cell to increase secretion capacity and protein synthesis (Liu et al. 2015). In two recent publications, improved heterologous protein production was demonstrated in ∆*rox1* knockout mutants of *S. cerevisiae* and *K. phaffii* cell factories (Liu et al. 2015, 2021), and the authors of the study in *S. cerevisiae* ascribe the mechanisms to alteration in the lipid composition resulting in more secretory vesicles and an acceleration of protein synthesis through derepression of *Anaerobically induced* gene (*ANB1*) (Liu et al. 2015).

Another genetic factor likely impacting the cellular response to burdensome heterologous protein production is the choice of signal peptide (Obst et al. 2017, Barrero et al. 2021). We used the most common signal peptide, the *S. cerevisiae* ⍺ mating factor (⍺MF). However, in a recent study in *K. phaffii* it was shown that the efficiency of ⍺MF can vary depending on the specific promoter and the downstream coding region (Obst et al. 2017). The ⍺MF signal peptide directs for posttranslation translocation into the endoplasmic reticulum (ER), opposed to other signal peptides that stimulate cotranslational translocation of the nascent peptide (Tang et al. 2015, Barrero et al. 2021). The timing of nascent peptide translocation into ER can contribute to burden. Here, we found that the cytosolic cochaperone, C4R3R2 protein, is upregulated in both the transcriptome and proteome of I1S and transcriptionally in I6G. The *S. cerevisiae* ortholog, *AHA1/*Aha1p, was also found to be upregulated in *S. cerevisiae* cell factories producing the same variant of hIP (Kazemi Seresht et al. 2013b). The authors of that study suggest *AHA1/*Aha1p upregulation is a cellular strategy to increase non-ER related folding capacity prior to ER translocation (Kazemi Seresht et al. 2013b). Thus, hIP might experience cytosolic containment by incomplete ER translocation or retranslocation from ER to cytosol for degradation. Apart from the *AHA1/*Aha1p ortholog, C4R0 × 7 is the second gene product/protein upregulated in both the transcriptome and proteome of I1S relative to I1G. This Hsp90 cochaperone is also found to activate Hsp70/Ssa1p ATPase activity, a cytosolic chaperone that mediates solubility and prevent aggregation of proteins directed to ER posttranslationally (Gasser et al. 2008). Whether upregulation of these chaperones and their cochaperones involved in maintaining protein homeostasis in the hIP strains can be ascribed to the signal peptide is unknown. However, selecting the optimal signal peptide can be challenging since its performance has been described to depend on both promoter and the protein of interest used in an expression cassette (Tang et al. 2015, Obst et al. 2017, Barrero et al. 2021).

Overall, based on DE analysis performed here, we found the most significant transcriptomic responses in the I1S strain. While the samples from two steady state time points (T1 and T2) showed similar profiles, we speculate that extending the fermentation and increasing the interval between samplings might have revealed differences, as over time, the imposed burden can disrupt steady state inducing population heterogeneity (Rugbjerg and Sommer 2019, Wright et al. 2022). For *S. cerevisiae*, a critical phase was observed during long-term chemostats of a cell factory after >35 generations. At this point, heterologous protein titres started to decrease and cell undergo metabolic shifts (Kazemi Seresht et al. 2013a). Direct comparison between the two studies is challenging due to species-specific differences in physiology and metabolic dynamics. Moreover, it remains unclear if such changes would occur earlier in *K. phaffii*. Consequently, we sampled at 13 generations into the feed phase (T2)—the latest feasible sampling point given the fermentation duration constraints in our lab—and confirmed that steady state remained at the time of second sampling (T2).

We found that the cellular response to heterologous protein production not only varies depending on the specific protein of interest but is also influenced by the gene dosage strategy and promoter selection used to drive transcription of the heterologous gene, as described before (Hohenblum et al. 2004). Here, the response pattern appears more similar between strains expressing the same heterologous gene (i.e. hIP) but with different promoters and gene dosage (i.e. I1S and I6G) compared to strains sharing gene dosage and promoter (i.e. gene copies and p_GAP_) but have different heterologous genes of interest (i.e. I6G and M6G).

### Engineering strategies for establishing robust cell factories with improved performance

As resource competition in cells engineered to express a heterologous product, can negatively impact the productivity of cell factories (Kazemi Seresht et al. 2013a, D’Ambrosio et al. 2020), strategies to ease this burden are relevant to improve cell factory performance (Kastberg et al. 2022). Since cellular metabolism is a highly regulated net of enzyme-catalysed reactions working to derive energy and building blocks for cellular maintenance and growth, cells need to be manipulated for the redirection of cellular resources towards heterologous product formation. Traditional engineering strategies to unburden cell factories involves the synthetic overexpression of limiting factors such as cofactors and transcription factors, and for this study *AHA1* could be relevant for I1S, or knocking out other factors, like *ROX1*, which can help ease the bottlenecks in the cell factories (Liu et al. 2021, Rußmayer et al. 2023).

However, at the same time, it is crucial to balance the host cell’s needs ensuring that it maintains sufficient growth capacity and avoids adaptive mechanisms that could reduce or escape production. Such strategies for mitigating burden in bacteria were recently reviewed by Grob et al. (2021). Some of these strategies could be adapted to yeast cell factories. The results presented here, can provide a starting point for establishing new genetic targets for enhancing strain robustness and performance. For example, by investigating transcriptomes, researchers can identify burden-responsive promoters that can be used for the engineering of burden-driven feedback regulation loops to improve the robustness of production strains and ideally the production of heterologous protein, as already demonstrated in bacteria (Ceroni et al. 2018). To engineer such burden-driven negative feedback, we must identify burden-responsive promoters by investigating the list of differentially expressed genes in the transcriptomes of burdened cell factories relative to low producer strains and reference strain. To relieve the pressure on respiration shared in all three high-producer strains (M6G, I6G, and I1S) presented here, we can tune expression of the heterologous gene as a response to respiration-related genes being upregulated. We propose the promoter driving transcription of the gene encoding *HSP10 S. cerevisiae* ortholog with the locus tag PAS_chr1-4_0163 (C4QXM0 protein product) as a relevant candidate for engineering burden-driven feedback regulation. This specific gene is expressed at very low levels in the reference strain and the low producer strains and is significantly upregulated in all three high-producer strains. Importantly, although this gene is upregulated, it is still expressed at relatively low levels, meaning that negative feedback regulation should not be too strong. This cochaperonin was also found to be significantly upregulated in high secretion strains of the nonconventional yeast *Y. lipolytica* (Korpys-Woźniak and Celińska 2021). Thus, the promoter for the gene encoding *HSP10* could be interesting to study further as a possible universal burden-responsive module in secreting yeast cell factories.

In this study, we demonstrate that one expression cassette utilizing the strong novel promoter P*_SPI1_* produces the same level of heterologous hIP transcripts as six expression cassettes driven by the P*_GAP_* promoter. While using several copies of the same promoter, such as P_GAP_, can cause competition for the same transcriptional resources and have a negative effect on transcriptional activity of the target gene (Dou et al. 2021), using just one cassette with an ultrastrong promoter like P_SPI1_ can be a risky strategy due to escape routes such as recombination or epigenetic silencing of the promoter, which can be problematic during long-term cultivations (Rugbjerg and Sommer 2019). A way to circumvent these challenges could be to engineer synthetic ‘addiction circuits’ into the host genome rendering the cell addicted to production of the heterologous product by coupling growth to heterologous production (Grob et al. 2021). Such strategy was applied in fitness-burdened yeast cell factories, where an essential gene was coupled to expression of pathway intermediates for vanillin production, and shown to control evolutionary drift, thus prolonging the productive lifespan and enhancing the production of the cell factories (D’Ambrosio et al. 2020). A similar principle could prove valuable for building more burden-resistant strains producing a heterologous protein.

Lastly, in the M6G strain, a rate-limiting resource competition at the ribosomal level is inferred from the upregulation in ribosomal genes. To overcome such bottleneck, resource allocation controllers using orthogonal ribosomes proved to reduce burden in bacteria (Darlington et al. 2018). Transferring this kind of controller to translation burdened yeast cell factories, by allocating translation of synthetic genes to a different translation pool and controlling gene expression by varying the pool size of the orthogonal ribosome, could prove valuable for the engineering of robust yeast cell factories.

## Conclusions

The burden imposed by heterologous protein production is a critical consideration in biomanufacturing. The process from transcription to protein secretion includes several steps, which each require a complex network of factors that can create bottlenecks during expression. The work presented here provides new insights into the physiological and molecular response to secretion of heterologous proteins in *K. phaffii*. We use two similar peptide-like heterologous proteins to characterize burden. Despite their similarity, they induce different burden responses. Our results suggest that protein structure and amino acid (codon usage) composition affect the optimal expression levels, leading to different cellular reactions in response to the overproduction of heterologous proteins. By characterizing the burden arising from two different heterologous proteins in *K. phaffii*, we expect the knowledge gained here will contribute to the development of improved, burden-resistant production strains, by hypothesis-driven strain engineering.

## Supplementary Material

foaf007_Supplemental_File

## Data Availability

All RNA-seq raw files generated from this study have been deposited to the NCBI (Sayers et al. 2022) BioProject database with BioProject accession number PRJNA1144800 (https://www.ncbi.nlm.nih.gov/sra/PRJNA1144800). The mass spectrometry proteomics data have been deposited to the ProteomeXchange Consortium via the PRIDE (Perez-Riverol et al. 2022) partner repository with the dataset identifier PXD055378 and 10.6019/PXD055378 and the secretomics data PXD055382 and 10.6019/PXD055382. Source code for BioLector data analysis and figures are publicly available at GitHub with the repository identifier DOI: 10.5281/zenodo.13625781.
