# Supplementary material for: Characterizing heterologous protein burden in Komagataella phaffii
Source: FEMS Yeast Res. 2025 Feb 19;25:foaf007. doi: 10.1093/femsyr/foaf007 (PMC11881926; doi:10.1093/femsyr/foaf007)
Supplement: foaf007_Supplemental_File [file foaf007_supplemental_file.docx]

Characterizing heterologous protein burden in *Komagataella phaffii*

**Supplementary material**

Louise La Barbera Kastberg^1,2^, Irene Hjorth Jacobsen^1^, Emre Özdemir^2^, Christopher T. Workman^1,3^, Michael Krogh Jensen^2^, Jochen Förster^2^*

^1^Department of Biotechnology and Biomedicine, Technical University of Denmark, Lyngby, Denmark

^2^Novo Nordisk Foundation Center for Biosustainability, Technical University of Denmark, Lyngby, Denmark

^3^Present address: Tailwind Biotech, Ørestads Boulevard 55 A 3 301, 2300 København S,

Denmark

**Supplementary Figure 1.** **Codon usage scores in all *K. phaffii* genes and native and codon optimized heterologous genes**. Nc and tAI scores for all 5040 *K. phaffii* GS115 open reading frames plotted. Nc and tAI scores for native (green), Genewiz codon optimized (orange), Twist codon optimized (blue), and IDT codon optimized (yellow) sequences of the heterologous genes hIP and Mambalgin-1 are plotted as well. Abbreviations: Nc (number of effective codons), tAI (tRNA adaptation index), PCC (Pearson Correlation Coefficient), (hIP) human Insulin Precursor. This plot is adapted from (Kastberg et al., 2024).

**Supplementary Figure 2**. **Growth profiles from continuous cultivations**. Growth profiles are plotted as carbon evolution rate (CER) against time (h) and dotted vertical lines indicate sampling time 1 (T1) and time 2 (T2) for following strains: GS115 (reference strain), Insulin 1 cassette P_GAP_ (I1G), Insulin 6 cassettes P_GAP_ (I6G), Insulin 1 cassette P_SPI1_ (I1S), Mambalgin-1 1 cassette P_GAP_ (M1G), and Mambalgin-1 6 cassettes P_GAP_ (M6G).

**Supplementary Figure 3. RNA-seq** **time point correlation plots.** Scatter plot comparisons of two time points of normalized RNA-seq mean log_2_ counts from biological triplicates of A) GS115, B) I1G, C) I6G, D) I1S, E) M1G, F) M6G. Correlation between sampling time points in all strains is assessed with R^2^ value.

**
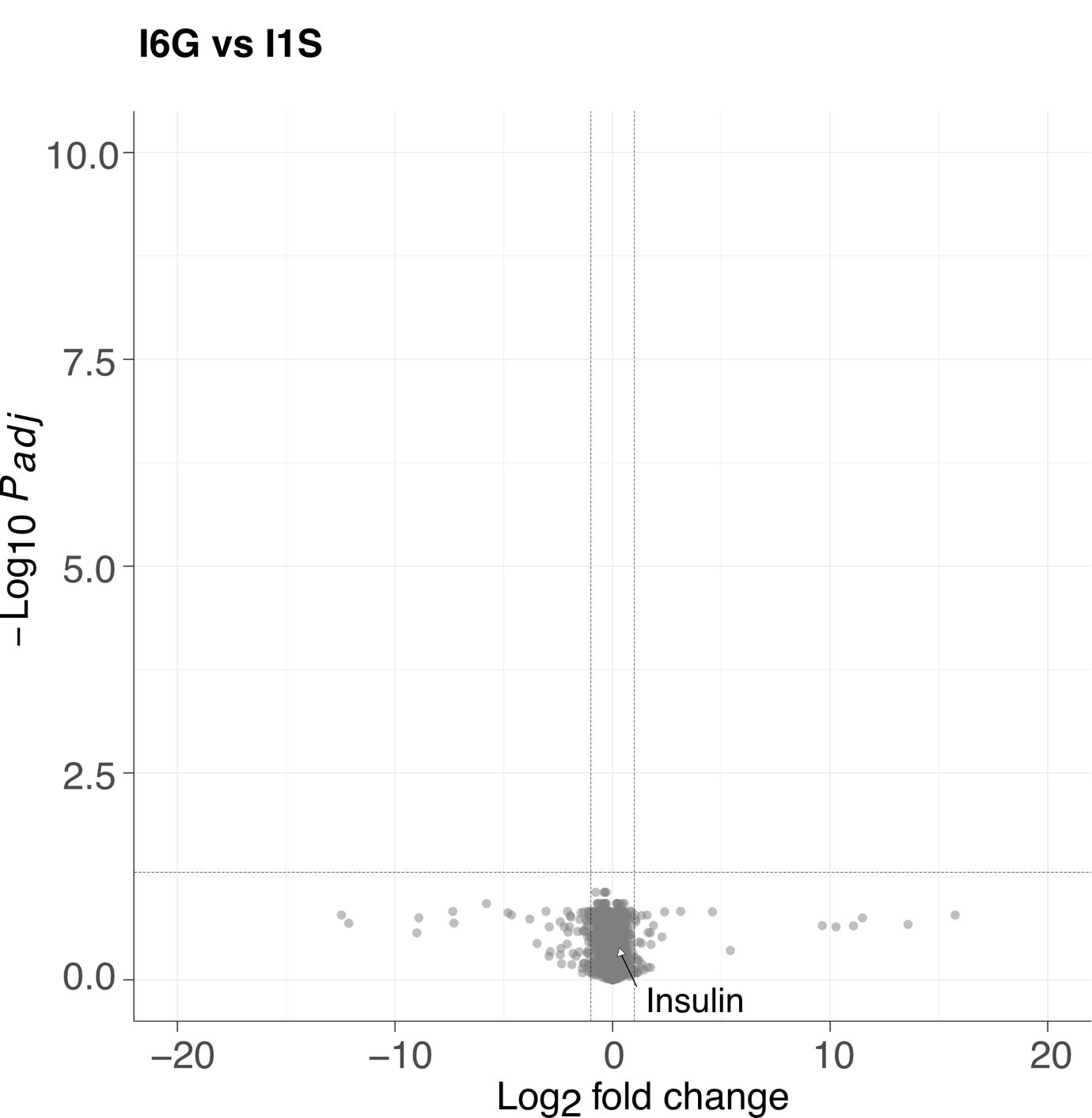
**

**Supplementary Figure 4. Insulin high-producer strains transcriptome differentially expression analysis**. Volcano plot displaying differentially expressed genes in I1S relative to I6G strain. Blue points indicate downregulation and red indicates upregulation (Transcript level, cutoff values (dashed lines) at |log_2_FC| >1 and adjusted p-value<0.05) blue = downregulated, red = upregulated, grey = NA.

**Supplementary Figure 5**. **Proteomics correlation plots.** Scatter plot comparisons of intracellular proteomics log2 normalized abundances from biological triplicates from two different time point samples (T1 and T2) showing correlation (Pearson correlation coefficient) between T1 and T2 in all strains and replicates for A) GS115 (reference strain), B) I1G, C) I1S, D) I6G (1outlier excluded), E) M1G, F) M6G.

**Supplementary Figure 6**. **Extracellular proteomics correlation plots.** Scatter plot comparisons of extracellular proteomics log2 normalized abundances from biological triplicates from two different time point samples (T1 and T2) showing correlation (Pearson correlation coefficient) between T1 and T2 in all strains and replicates for A) GS115 (reference strain) (3 outliers excluded), B) I1G, C) I1S, D) I6G (2 outliers excluded), E) M1G (1 outlier excluded), F) M6G.

**Supplementary Table 1**. Plasmids used in this study are listed.

| Plasmid name | Plasmid entity | Description | Reference |
| --- | --- | --- | --- |
| pCas9 | pDIV151 | Kp_GAPp_hsCas9_NatOPT plasmids | (Strucko et al., 2024) |
| pCas9-UidAsgRNA | pDIV272 | pFBF003_gRNA_uidA#2_Kp_hsCas9_NatOPT | (Strucko et al., 2024) |
| pCI-1sgRNA | pLK0015 | Cas9, Kp sgRNA CI 1.3 | (Kastberg et al., 2024) |
| pCI-2sgRNA | pLK0016 | Cas9, Kp sgRNA CI 2.1 | (Kastberg et al., 2024) |
| pCII-1sgRNA | pLK0025 | Cas9, Kp sgRNA CII 1.1 | (Kastberg et al., 2024) |
| pCIII-1sgRNA | pLK0031 | Cas9, Kp sgRNA CIII 1.3 | (Kastberg et al., 2024) |
| pCIV-1sgRNA | pLK0034 | Cas9, Kp sgRNA CIV 1.2 | (Kastberg et al., 2024) |
| pCIV-2sgRNA | pLK0040 | Cas9, Kp sgRNA CIV 2.4 | (Kastberg et al., 2024) |
| pCI2-CII1-CIV2sgRNA | pLK0084 | Cas9, Kp multiplex sgRNA CIV-2 CII-1 CI-2 | This study |
| pCIII1-CIV1sgRNA | pLK0085 | Cas9, Kp multiplex sgRNA CIV-1, CIII-1 | This study |

**Supplementary Table 2**. Gene, promoter, and sgRNA sequences. ⍺MF (*S. cerevisiae* ⍺ mating factor signal peptide),

| Fragment name | Sequence | Description |
| --- | --- | --- |
| CI2-CII1-CIV2sgRNA | TAACTCTGGCAACCAGTAACACGCTTAAGGTTTGGAACAACACTAAACTACCTTGCGGTACTACCATTGACACTACACATCCTTAATTCCAATCCTGTCTGGCCTCCTTCACCTTTTAACCATCTTGCCCATTCCAACTCGTGTCAGATTGCGTATCAAGTGAAAAAAAAAAATTTTAAATCTTTAACCCAATCAGGTAATAACTGTCGCCTCTTTTATCTGCCGCACTGCATGAGGTGTCCCCTTAGTGGGAAAGAGTACTGAGCCAACCCTGGAGGACAGCAAGGGAAAAATACCTACAACTTGCTTCATAATGGTCGTAAAAACAATCCTTGTCGGATATAAGTGTTGTAGACTGTCCCTTATCCTCTGCGATGTTCTTCCTCTCAAAGTTTGCGATTTCTCTCTATCAGAATTGCCATCAAGAGACTCAGGACTAATTTCGCAGTCCCACACGCACTCGTACATGATTGGCTGAAATTTCCCTAAAGAATTTCTTTTTCACGAAAATTTTTTTTTTACACAAGATTTTCAGCAGATATAAAATGGAGAGCAGGACCTCCGCTGTGACTCTTCTTTTTTTTCTTTTATTCTCACTACATACATTTTAGTTATTCGCCAACgCATCATTGGTCTAGTGGTAGAATTCATCGTTGCCATCGATGAGGCCCGTGTTCGaTTCACGGAtGATGCAaactgcactgtgtaacgctgGTTTTAGAGCTAGAAATAGCAAGTTAAAATAAGGCTAGTCCGTTATCAACTTGAAAAAGTGGCACCGAGTCGGTGCTTTTGCATCATTGGTCTAGTGGTAGAATTCATCGTTGCCATCGATGAGGCCCGTGTTCGATTCACGGATGATGCaaaaatcatcggttggttgggGTTTTAGAGCTAGAAATAGCAAGTTAAAATAAGGCTAGTCCGTTATCAACTTGAAAAAGTGGCACCGAGTCGGTGCTTTTgCATCATTGGTCTAGTGGTAGAATTCATCGTTGCCATCGATGAGGCCCGTGTTCGaTTCACGGAtGATGCAgctcccaagaataactcggtGTTTTAGAGCTAGAAATAGCAAGTTAAAATAAGGCTAGTCCGTTATCAACTTGAAAAAGTGGCACCGAGTCGGTGCTTTTGCATCATTGGTCTAGTGGTAGAATTCATCGTTGCCATCGATGAGGCCCGTGTTCGATTCACGGATGATGCaTCAAGAGGAtGTCAGAATGCCATTTGCCTGAGAGATGCAGGCTTCATTTTTGATACTTTTTTATTTGTAACCTATATAGTATAGGATTTTTTTTG | Multiplex sgRNA cassette inserted in backbone pDIV151. crRNA for integration site CI-2, CII-1, and CIV-2 are denoted in lower case letters. |
| pCIII1-CIV1sgRNA | TAACTCTGGCAACCAGTAACACGCTTAAGGTTTGGAACAACACTAAACTACCTTGCGGTACTACCATTGACACTACACATCCTTAATTCCAATCCTGTCTGGCCTCCTTCACCTTTTAACCATCTTGCCCATTCCAACTCGTGTCAGATTGCGTATCAAGTGAAAAAAAAAAATTTTAAATCTTTAACCCAATCAGGTAATAACTGTCGCCTCTTTTATCTGCCGCACTGCATGAGGTGTCCCCTTAGTGGGAAAGAGTACTGAGCCAACCCTGGAGGACAGCAAGGGAAAAATACCTACAACTTGCTTCATAATGGTCGTAAAAACAATCCTTGTCGGATATAAGTGTTGTAGACTGTCCCTTATCCTCTGCGATGTTCTTCCTCTCAAAGTTTGCGATTTCTCTCTATCAGAATTGCCATCAAGAGACTCAGGACTAATTTCGCAGTCCCACACGCACTCGTACATGATTGGCTGAAATTTCCCTAAAGAATTTCTTTTTCACGAAAATTTTTTTTTTACACAAGATTTTCAGCAGATATAAAATGGAGAGCAGGACCTCCGCTGTGACTCTTCTTTTTTTTCTTTTATTCTCACTACATACATTTTAGTTATTCGCCAACgCATCATTGGTCTAGTGGTAGAATTCATCGTTGCCATCGATGAGGCCCGTGTTCGaTTCACGGAtGATGCAcatctcatcctttagcaccgGTTTTAGAGCTAGAAATAGCAAGTTAAAATAAGGCTAGTCCGTTATCAACTTGAAAAAGTGGCACCGAGTCGGTGCTTTTGCATCATTGGTCTAGTGGTAGAATTCATCGTTGCCATCGATGAGGCCCGTGTTCGATTCACGGATGATGCattaccaagaatgaaacgcttGTTTTAGAGCTAGAAATAGCAAGTTAAAATAAGGCTAGTCCGTTATCAACTTGAAAAAGTGGCACCGAGTCGGTGCTTTTGCATCATTGGTCTAGTGGTAGAATTCATCGTTGCCATCGATGAGGCCCGTGTTCGATTCACGGATGATGCaTCAAGAGGAtGTCAGAATGCCATTTGCCTGAGAGATGCAGGCTTCATTTTTGATACTTTTTTATTTGTAACCTATATAGTATAGGATTTTTTTTG | Multiplex sgRNA cassette inserted in backbone pDIV151. crRNA for integration site CIII-1 and CIV-1 are denoted in lower case letters. |
| Human insulin pre-cursor | ATGAGATTTCCTTCAATTTTTACTGCAGTTTTATTCGCAGCATCCTCCGCATTAGCTGCTCCAGTCAACACTACAACAGAAGATGAAACGGCACAAATTCCGGCTGAAGCTGTCATCGGTTACTTAGATTTAGAAGGGGATTTCGATGTTGCTGTTTTGCCATTTTCCAACAGCACAAATAACGGGTTATTGTTTATAAATACTACTATTGCCAGCATTGCTGCTAAAGAAGAAGGGGTATCTTTGGATAAAAGAGAGGAGGCTGAAGCAGAGGCTCCAAAGTTTGTTAATCAACATTTGTGTGGTTCTCATTTGGTTGAAGCTTTGTATTTGGTTTGTGGTGAAAGAGGTTTTTTTTATACTCCAAAAGAATGGAAAGGTATTGTTGAACAATGTTGTACTTCTATTTGTTCTTTGTATCAATTGGAAAATTATTGTAATCATCATCACCATCATCAC | Codon optimised insulin precursor (EWK c-chain) with a Sc ⍺MF signal peptide, spacer peptide, and C-terminal his tag |
| Mambalgin-1 his tag | ATGAGATTTCCTTCAATTTTTACTGCAGTTTTATTCGCAGCATCCTCCGCATTAGCTGCTCCAGTCAACACTACAACAGAAGATGAAACGGCACAAATTCCGGCTGAAGCTGTCATCGGTTACTTAGATTTAGAAGGGGATTTCGATGTTGCTGTTTTGCCATTTTCCAACAGCACAAATAACGGGTTATTGTTTATAAATACTACTATTGCCAGCATTGCTGCTAAAGAAGAAGGGGTATCTTTGGATAAAAGAGAGGCTGAAGCTTTGAAATGTTATCAGCATGGGAAAGTAGTCACTTGTCATAGGGACATGAAGTTTTGTTACCATAACACCGGTATGCCATTTAGAAATTTGAAATTGATTTTGCAAGGTTGTTCTTCTTCTTGTTCTGAAACTGAAAATAATAAATGTTGTTCTACTGATAGATGTAATAAACATCATCACCATCATCAC | Codon optimised Mambalgin-1 with a Sc ⍺MF signal peptide and C-terminal his tag |
| GAP promoter | TTTTTGTAGAAATGTCTTGGTGTCCTCGTCCAATCAGGTAGCCATCTCTGAAATATCTGGCTCCGTTGCAACTCCGAACGACCTGCTGGCAACGTAAAATTCTCCGGGGTAAAACTTAAATGTGGAGTAATGGAACCAGAAACGTCTCTTCCCTTCTCTCTCCTTCCACCGCCCGTTACCGTCCCTAGGAAATTTTACTCTGCTGGAGAGCTTCTTCTACGGCCCCCTTGCAGCAATGCTCTTCCCAGCATTACGTTGCGGGTAAAACGGAGGTCGTGTACCCGACCTAGCAGCCCAGGGATGGAAAAGTCCCGGCCGTCGCTGGCAATAATAGCGGGCGGACGCATGTCATGAGATTATTGGAAACCACCAGAATCGAATATAAAAGGCGAACACCTTTCCCAATTTTGGTTTCTCCTGACCCAAAGACTTTAAATTTAATTTATTTGTCCCTATTTCAATCAATTGAACAACTAT |  |
| SPI1 promoter | ATACTATTCCTCCGCTCGTTTCTTTTTTCAGTGAGGTGTGTCGTGAAAGAAAACCCACAATTAAAGTGGTTTCCAGGACAACACCCAAAAAAAGGTATCAATGCCACTAGGCAGTCGGTTTTATTTTTGGTCACCCACGCAAAGAAGCACCCACCTCTTTTAGGTTTTAAGTTGTGGGAACAGTAACACCGCCTAGAGCTTCAGGAAAAACCAGTACCTGTGACCGCAATTCACCATGATGCAGAATGTTAATTTAAACGAGTGCCAAATCAAGATTTCAACAGACAAATCAATCGATCCATAGTTACCCATTCCAGCCTTTTCGTCGTCGAGCCTGCTTCATTCCTGCCTCAGGTGCATAACTTTGCATGAAAAGTCCAGATTAGGGCAGATTTTGAGTTTAAAATAGGAAATATAAACAAATATACCGCGAAAAAGGTTTGTTTATAGCTTTTCGCCTGGTGCCGTACGGTATAAATACATACTCTCCTCCCCCCCCTGGTTCTCTTTTTCTTTTGTTACTTACATTTTACCGTTCCGTCACTCGCTTCACTCAACAACAAAAATGTCTAAAGGTGAAGAATTATTCACTGGTGTTGTCCCAATTTTGGTTGAATTAGATGGTGATGT |  |
| AOX1 terminator | TCAAGAGGATGTCAGAATGCCATTTGCCTGAGAGATGCAGGCTTCATTTTTGATACTTTTTTATTTGTAACCTATATAGTATAGGATTTTTTTTGTCATTTTGTTTCTTCTCGTACGAGCTTGCTCCTGATCAGCCTATCTCGCAGCTGATGAATATCTTGTGGTAGGGGTTTGGGAAAATCATTCGAGTTTGATGTTTTTCTTGGTATTTCCCACTCCTCTTCAGAGTACAGAAGATTAAGTGAGA |  |

**Supplementary Table 3**. Mean values of max biomass measured from BioLector results for each strain. SD = standard deviation.

| Group | Mean | SD |
| --- | --- | --- |
| GS115 | 79.90 | 0.66 |
| I1G | 78.21 | 2.20 |
| I1S | 61.00 | 2.16 |
| I6G | 59.67 | 1.56 |
| M1G | 88.25 | 0.67 |
| M6G | 76.60 | 0.89 |

**Supplementary Table 4**. Pairwise t-test of max biomass from BioLector. n(1 or 2) = sample count for ref group (1) and compared strain (2), df = degrees of freedom, p = p-value, p.adj = adjusted p-value.

| Ref strain | Strain | n1 | n2 | stat | df | p | p.adj | p.adj.signif |
| --- | --- | --- | --- | --- | --- | --- | --- | --- |
| GS115 | I1G | 3 | 6 | 1.730 | 6.4446 | 0.131 | 0.131 | ns |
| GS115 | I1S | 3 | 6 | 19.647 | 6.4771 | 5.13E-07 | 3.08E-06 | **** |
| GS115 | I6G | 3 | 6 | 27.239 | 6.9841 | 2.38E-08 | 1.90E-07 | **** |
| GS115 | M1G | 3 | 6 | -17.773 | 4.1597 | 4.42E-05 | 0.000221 | *** |
| GS115 | M6G | 3 | 6 | 6.275 | 5.4569 | 0.001 | 0.003 | ** |
| I1G | I1S | 6 | 6 | 13.670 | 9.9974 | 8.53E-08 | 5.12E-07 | **** |
| I1G | I6G | 6 | 6 | 16.843 | 9.0165 | 4.02E-08 | 2.81E-07 | **** |
| I1G | M1G | 6 | 6 | -10.699 | 5.9272 | 4.26E-05 | 0.000213 | *** |
| I1G | M6G | 6 | 6 | 1.668 | 6.5872 | 0.142 | 0.262 | ns |
| I1G | GS115 | 6 | 3 | -1.730 | 6.4446 | 0.131 | 0.262 | ns |
| M1G | I1G | 6 | 6 | 10.699 | 5.9272 | 4.26E-05 | 0.00017 | *** |
| M1G | I1S | 6 | 6 | 29.472 | 5.9573 | 1.11E-07 | 5.55E-07 | **** |
| M1G | I6G | 6 | 6 | 41.208 | 6.7958 | 2.08E-09 | 1.46E-08 | **** |
| M1G | M6G | 6 | 6 | 25.644 | 9.3177 | 5.85E-10 | 4.68E-09 | **** |
| M1G | GS115 | 6 | 3 | 17.773 | 4.1597 | 4.42E-05 | 0.00017 | *** |

**Supplementary Table 5.** Residual glucose and by-product concentrations, biomass yield, and specific CO_2_ yield. Mean values of triplicates (n=3) ± standard deviation for values in time point T1 and T2.

|  | Glucose  (g L^-1^) | | Ethanol  (g L^-1^) | | Acetate  (g L^-1^) | | Biomass yield  (g_cdw_ g^-1^_glucose_) | | CO_2_ yield  (g_CO2_ g^-1^_cDW_) | |
| --- | --- | --- | --- | --- | --- | --- | --- | --- | --- | --- |
|  | T1 | T2 | T1 | T2 | T1 | T2 | T1 | T2 | T1 | T2 |
| Reference strain (GS115) | 0.140±0.01 | 0.135±0.02 | 0.234±0.03 | 0.225±0.05 | 0.321±0.03 | 0.309±0.05 | 0.58±0.15 | 0.6±0.1 | 0.72±0.15 | 0.67±0.09 |
| I1G | 0.143±0.01 | 0.144±0.01 | 0.243±0.02 | 0.244±0.02 | 0.328±0.02 | 0.331±0.02 | 0.62±0.2 | 0.54±0.16 | 0.7±0.18 | 0.78±0.22 |
| I6G | 0.142±0.01 | 0.140±0.00 | 0.226±0.02 | 0.227±0.01 | 0.322±0.02 | 0.316±0.01 | 0.6±0.12 | 0.56±0.1 | 0.68±0.09 | 0.72±0.07 |
| I1S | 0.152±0.00 | 0.149±0.01 | 0.250±0.01 | 0.264±0.02 | 0.349±0.01 | 0.341±0.02 | 0.54±0.04 | 0.56±0.04 | 0.74±0.03 | 0.71±0.03 |
| M1G | 0.155±0.01 | 0.1610.00 | 0.256±0.01 | 0.228±0.01 | 0.355±0.01 | 0.368±0.01 | 0.51±0.01 | 0.51±0.04 | 0.78±0.03 | 0.79±0.04 |
| M6G | 0.138±0.00 | 0.139±0.00 | 0.225±0.00 | 0.245±0.00 | 0.313±0.00 | 0.314±0.00 | 0.52±0.00 | 0.55±0.03 | 0.77±0.04 | 0.73±0.02 |

**Supplementary Table 6**. Pairwise t-tests of chemostat byproduct concentrations between strains and sample times. Sample time T1 and T2 are indicated as “1” or “2” after strain names. n(1 or 2) = sample count for ref group (1) and compared strain (2), df = degrees of freedom, p = p-value, p.adj = adjusted p-value.

| Product | Ref strain | Strain | n1 | n2 | stat | df | p | p.adj | p.adj.signif |
| --- | --- | --- | --- | --- | --- | --- | --- | --- | --- |
| Glucose | GS1151 | GS1152 | 3 | 3 | -0.007 | 3.999 | 0.995 | 1 | ns |
| Glucose | GS1151 | I1G1 | 3 | 3 | 0.036 | 2.710 | 0.974 | 1 | ns |
| Glucose | GS1151 | I1G2 | 3 | 3 | -0.425 | 2.023 | 0.711 | 1 | ns |
| Glucose | GS1151 | I1S1 | 3 | 3 | -0.841 | 2.048 | 0.487 | 1 | ns |
| Glucose | GS1151 | I1S2 | 3 | 3 | -0.847 | 2.047 | 0.485 | 1 | ns |
| Glucose | GS1151 | I6G1 | 3 | 3 | -0.828 | 2.210 | 0.487 | 1 | ns |
| Glucose | GS1151 | I6G2 | 3 | 3 | -0.833 | 2.192 | 0.486 | 1 | ns |
| Glucose | GS1151 | M1G1 | 3 | 3 | 0.435 | 3.156 | 0.691 | 1 | ns |
| Glucose | GS1151 | M1G2 | 3 | 3 | 0.444 | 3.184 | 0.685 | 1 | ns |
| Glucose | GS1151 | M6G1 | 3 | 3 | -0.940 | 2.169 | 0.44 | 1 | ns |
| Glucose | GS1151 | M6G2 | 3 | 3 | -0.939 | 2.170 | 0.44 | 1 | ns |
| Glucose | I1G1 | GS1151 | 3 | 3 | -0.036 | 2.710 | 0.974 | 1 | ns |
| Glucose | I1G1 | GS1152 | 3 | 3 | -0.046 | 2.729 | 0.967 | 1 | ns |
| Glucose | I1G1 | I1G2 | 3 | 3 | -1.072 | 2.126 | 0.39 | 1 | ns |
| Glucose | I1G1 | I1S1 | 3 | 3 | -2.004 | 2.258 | 0.168 | 1 | ns |
| Glucose | I1G1 | I1S2 | 3 | 3 | -2.017 | 2.256 | 0.167 | 1 | ns |
| Glucose | I1G1 | I6G1 | 3 | 3 | -1.830 | 3.060 | 0.163 | 1 | ns |
| Glucose | I1G1 | I6G2 | 3 | 3 | -1.854 | 2.984 | 0.161 | 1 | ns |
| Glucose | I1G1 | M1G1 | 3 | 3 | 0.650 | 3.730 | 0.554 | 1 | ns |
| Glucose | I1G1 | M1G2 | 3 | 3 | 0.661 | 3.704 | 0.548 | 1 | ns |
| Glucose | I1G1 | M6G1 | 3 | 3 | -2.104 | 2.878 | 0.13 | 1 | ns |
| Glucose | I1G1 | M6G2 | 3 | 3 | -2.100 | 2.883 | 0.13 | 1 | ns |
| Glucose | I6G1 | GS1151 | 3 | 3 | 0.828 | 2.210 | 0.487 | 1 | ns |
| Glucose | I6G1 | GS1152 | 3 | 3 | 0.830 | 2.216 | 0.487 | 1 | ns |
| Glucose | I6G1 | I1G1 | 3 | 3 | 1.830 | 3.060 | 0.163 | 1 | ns |
| Glucose | I6G1 | I1G2 | 3 | 3 | 1.750 | 2.435 | 0.199 | 1 | ns |
| Glucose | I6G1 | I1S1 | 3 | 3 | 0.013 | 2.860 | 0.99 | 1 | ns |
| Glucose | I6G1 | I1S2 | 3 | 3 | -0.008 | 2.852 | 0.994 | 1 | ns |
| Glucose | I6G1 | I6G2 | 3 | 3 | -0.010 | 3.993 | 0.992 | 1 | ns |
| Glucose | I6G1 | M1G1 | 3 | 3 | 2.216 | 2.644 | 0.125 | 1 | ns |
| Glucose | I6G1 | M1G2 | 3 | 3 | 2.207 | 2.626 | 0.127 | 1 | ns |
| Glucose | I6G1 | M6G1 | 3 | 3 | -0.359 | 3.954 | 0.738 | 1 | ns |
| Glucose | I6G1 | M6G2 | 3 | 3 | -0.354 | 3.957 | 0.741 | 1 | ns |
| Glucose | I1S1 | GS1151 | 3 | 3 | 0.841 | 2.048 | 0.487 | 1 | ns |
| Glucose | I1S1 | GS1152 | 3 | 3 | 0.843 | 2.049 | 0.486 | 1 | ns |
| Glucose | I1S1 | I1G1 | 3 | 3 | 2.004 | 2.258 | 0.168 | 1 | ns |
| Glucose | I1S1 | I1G2 | 3 | 3 | 3.154 | 3.576 | 0.04 | 0.441 | ns |
| Glucose | I1S1 | I1S2 | 3 | 3 | -0.036 | 4.000 | 0.973 | 1 | ns |
| Glucose | I1S1 | I6G1 | 3 | 3 | -0.013 | 2.860 | 0.99 | 1 | ns |
| Glucose | I1S1 | I6G2 | 3 | 3 | -0.027 | 2.929 | 0.98 | 1 | ns |
| Glucose | I1S1 | M1G1 | 3 | 3 | 2.342 | 2.149 | 0.135 | 1 | ns |
| Glucose | I1S1 | M1G2 | 3 | 3 | 2.330 | 2.145 | 0.137 | 1 | ns |
| Glucose | I1S1 | M6G1 | 3 | 3 | -0.490 | 3.040 | 0.658 | 1 | ns |
| Glucose | I1S1 | M6G2 | 3 | 3 | -0.483 | 3.034 | 0.662 | 1 | ns |
| Glucose | M6G1 | GS1151 | 3 | 3 | 0.940 | 2.169 | 0.44 | 1 | ns |
| Glucose | M6G1 | GS1152 | 3 | 3 | 0.944 | 2.174 | 0.438 | 1 | ns |
| Glucose | M6G1 | I1G1 | 3 | 3 | 2.104 | 2.878 | 0.13 | 1 | ns |
| Glucose | M6G1 | I1G2 | 3 | 3 | 2.430 | 2.537 | 0.109 | 1 | ns |
| Glucose | M6G1 | I1S1 | 3 | 3 | 0.490 | 3.040 | 0.658 | 1 | ns |
| Glucose | M6G1 | I1S2 | 3 | 3 | 0.466 | 3.031 | 0.672 | 1 | ns |
| Glucose | M6G1 | I6G1 | 3 | 3 | 0.359 | 3.954 | 0.738 | 1 | ns |
| Glucose | M6G1 | I6G2 | 3 | 3 | 0.356 | 3.983 | 0.74 | 1 | ns |
| Glucose | M6G1 | M1G1 | 3 | 3 | 2.431 | 2.523 | 0.109 | 1 | ns |
| Glucose | M6G1 | M1G2 | 3 | 3 | 2.419 | 2.509 | 0.111 | 1 | ns |
| Glucose | M6G1 | M6G2 | 3 | 3 | 0.004 | 4.000 | 0.997 | 1 | ns |
| Glucose | M6G1 | GS1151 | 3 | 3 | 0.940 | 2.169 | 0.44 | 1 | ns |
| Glucose | M6G1 | GS1152 | 3 | 3 | 0.944 | 2.174 | 0.438 | 1 | ns |
| Glucose | M6G1 | I1G1 | 3 | 3 | 2.104 | 2.878 | 0.13 | 1 | ns |
| Glucose | M6G1 | I1G2 | 3 | 3 | 2.430 | 2.537 | 0.109 | 1 | ns |
| Glucose | M6G1 | I1S1 | 3 | 3 | 0.490 | 3.040 | 0.658 | 1 | ns |
| Glucose | M6G1 | I1S2 | 3 | 3 | 0.466 | 3.031 | 0.672 | 1 | ns |
| Glucose | M6G1 | I6G1 | 3 | 3 | 0.359 | 3.954 | 0.738 | 1 | ns |
| Glucose | M6G1 | I6G2 | 3 | 3 | 0.356 | 3.983 | 0.74 | 1 | ns |
| Glucose | M6G1 | M1G1 | 3 | 3 | 2.431 | 2.523 | 0.109 | 1 | ns |
| Glucose | M6G1 | M1G2 | 3 | 3 | 2.419 | 2.509 | 0.111 | 1 | ns |
| Glucose | M6G1 | M6G2 | 3 | 3 | 0.004 | 4.000 | 0.997 | 1 | ns |
|  |  |  |  |  |  |  |  |  |  |
| Product | Ref strain | Strain | n1 | n2 | stat | df | p | p.adj | p.adj.signif |
| Acetate | GS1151 | GS1152 | 3 | 3 | 0.381 | 3.275 | 0.726 | 1 | ns |
| Acetate | GS1151 | I1G1 | 3 | 3 | -0.307 | 3.783 | 0.775 | 1 | ns |
| Acetate | GS1151 | I1G2 | 3 | 3 | -0.453 | 3.851 | 0.675 | 1 | ns |
| Acetate | GS1151 | I1S1 | 3 | 3 | -1.565 | 2.778 | 0.223 | 1 | ns |
| Acetate | GS1151 | I1S2 | 3 | 3 | -1.060 | 3.110 | 0.365 | 1 | ns |
| Acetate | GS1151 | I6G1 | 3 | 3 | -0.032 | 3.562 | 0.976 | 1 | ns |
| Acetate | GS1151 | I6G2 | 3 | 3 | 0.295 | 2.457 | 0.791 | 1 | ns |
| Acetate | GS1151 | M1G1 | 3 | 3 | -1.893 | 2.745 | 0.163 | 1 | ns |
| Acetate | GS1151 | M1G2 | 3 | 3 | -2.762 | 2.368 | 0.091 | 1 | ns |
| Acetate | GS1151 | M6G1 | 3 | 3 | 0.501 | 2.097 | 0.664 | 1 | ns |
| Acetate | GS1151 | M6G2 | 3 | 3 | 0.429 | 2.006 | 0.71 | 1 | ns |
| Acetate | I1G1 | GS1151 | 3 | 3 | 0.307 | 3.783 | 0.775 | 1 | ns |
| Acetate | I1G1 | GS1152 | 3 | 3 | 0.614 | 2.842 | 0.585 | 1 | ns |
| Acetate | I1G1 | I1G2 | 3 | 3 | -0.172 | 3.992 | 0.872 | 1 | ns |
| Acetate | I1G1 | I1S1 | 3 | 3 | -1.469 | 3.190 | 0.233 | 1 | ns |
| Acetate | I1G1 | I1S2 | 3 | 3 | -0.856 | 3.588 | 0.445 | 1 | ns |
| Acetate | I1G1 | I6G1 | 3 | 3 | 0.336 | 3.942 | 0.754 | 1 | ns |
| Acetate | I1G1 | I6G2 | 3 | 3 | 0.822 | 2.728 | 0.477 | 1 | ns |
| Acetate | I1G1 | M1G1 | 3 | 3 | -1.868 | 3.147 | 0.154 | 1 | ns |
| Acetate | I1G1 | M1G2 | 3 | 3 | -2.971 | 2.592 | 0.071 | 0.779 | ns |
| Acetate | I1G1 | M6G1 | 3 | 3 | 1.124 | 2.159 | 0.371 | 1 | ns |
| Acetate | I1G1 | M6G2 | 3 | 3 | 1.045 | 2.010 | 0.405 | 1 | ns |
| Acetate | I6G1 | GS1151 | 3 | 3 | 0.032 | 3.562 | 0.976 | 1 | ns |
| Acetate | I6G1 | GS1152 | 3 | 3 | 0.432 | 2.672 | 0.698 | 1 | ns |
| Acetate | I6G1 | I1G1 | 3 | 3 | -0.336 | 3.942 | 0.754 | 1 | ns |
| Acetate | I6G1 | I1G2 | 3 | 3 | -0.509 | 3.894 | 0.638 | 1 | ns |
| Acetate | I6G1 | I1S1 | 3 | 3 | -2.029 | 3.430 | 0.124 | 1 | ns |
| Acetate | I6G1 | I1S2 | 3 | 3 | -1.322 | 3.804 | 0.26 | 1 | ns |
| Acetate | I6G1 | I6G2 | 3 | 3 | 0.455 | 2.909 | 0.681 | 1 | ns |
| Acetate | I6G1 | M1G1 | 3 | 3 | -2.471 | 3.384 | 0.08 | 0.804 | ns |
| Acetate | I6G1 | M1G2 | 3 | 3 | -3.760 | 2.744 | 0.038 | 0.421 | ns |
| Acetate | I6G1 | M6G1 | 3 | 3 | 0.768 | 2.202 | 0.516 | 1 | ns |
| Acetate | I6G1 | M6G2 | 3 | 3 | 0.674 | 2.013 | 0.569 | 1 | ns |
| Acetate | I1S1 | GS1151 | 3 | 3 | 1.565 | 2.778 | 0.223 | 1 | ns |
| Acetate | I1S1 | GS1152 | 3 | 3 | 1.424 | 2.290 | 0.276 | 1 | ns |
| Acetate | I1S1 | I1G1 | 3 | 3 | 1.469 | 3.190 | 0.233 | 1 | ns |
| Acetate | I1S1 | I1G2 | 3 | 3 | 1.210 | 3.106 | 0.31 | 1 | ns |
| Acetate | I1S1 | I1S2 | 3 | 3 | 0.713 | 3.848 | 0.516 | 1 | ns |
| Acetate | I1S1 | I6G1 | 3 | 3 | 2.029 | 3.430 | 0.124 | 0.868 | ns |
| Acetate | I1S1 | I6G2 | 3 | 3 | 3.597 | 3.723 | 0.026 | 0.284 | ns |
| Acetate | I1S1 | M1G1 | 3 | 3 | -0.558 | 3.998 | 0.607 | 1 | ns |
| Acetate | I1S1 | M1G2 | 3 | 3 | -2.154 | 3.516 | 0.107 | 0.856 | ns |
| Acetate | I1S1 | M6G1 | 3 | 3 | 4.670 | 2.474 | 0.028 | 0.284 | ns |
| Acetate | I1S1 | M6G2 | 3 | 3 | 4.751 | 2.030 | 0.04 | 0.364 | ns |
| Acetate | M1G1 | GS1151 | 3 | 3 | 1.893 | 2.745 | 0.163 | 1 | ns |
| Acetate | M1G1 | GS1152 | 3 | 3 | 1.630 | 2.277 | 0.23 | 1 | ns |
| Acetate | M1G1 | I1G1 | 3 | 3 | 1.868 | 3.147 | 0.154 | 1 | ns |
| Acetate | M1G1 | I1G2 | 3 | 3 | 1.594 | 3.064 | 0.207 | 1 | ns |
| Acetate | M1G1 | I1S1 | 3 | 3 | 0.558 | 3.998 | 0.607 | 1 | ns |
| Acetate | M1G1 | I1S2 | 3 | 3 | 1.218 | 3.814 | 0.293 | 1 | ns |
| Acetate | M1G1 | I6G1 | 3 | 3 | 2.471 | 3.384 | 0.08 | 0.643 | ns |
| Acetate | M1G1 | I6G2 | 3 | 3 | 4.281 | 3.763 | 0.015 | 0.161 | ns |
| Acetate | M1G1 | M1G2 | 3 | 3 | -1.532 | 3.561 | 0.209 | 1 | ns |
| Acetate | M1G1 | M6G1 | 3 | 3 | 5.519 | 2.496 | 0.019 | 0.187 | ns |
| Acetate | M1G1 | M6G2 | 3 | 3 | 5.657 | 2.031 | 0.029 | 0.259 | ns |
| Acetate | M6G1 | GS1151 | 3 | 3 | -0.501 | 2.097 | 0.664 | 1 | ns |
| Acetate | M6G1 | GS1152 | 3 | 3 | 0.140 | 2.035 | 0.901 | 1 | ns |
| Acetate | M6G1 | I1G1 | 3 | 3 | -1.124 | 2.159 | 0.371 | 1 | ns |
| Acetate | M6G1 | I1G2 | 3 | 3 | -1.310 | 2.145 | 0.313 | 1 | ns |
| Acetate | M6G1 | I1S1 | 3 | 3 | -4.670 | 2.474 | 0.028 | 0.253 | ns |
| Acetate | M6G1 | I1S2 | 3 | 3 | -3.001 | 2.320 | 0.08 | 0.637 | ns |
| Acetate | M6G1 | I6G1 | 3 | 3 | -0.768 | 2.202 | 0.516 | 1 | ns |
| Acetate | M6G1 | I6G2 | 3 | 3 | -0.521 | 2.806 | 0.64 | 1 | ns |
| Acetate | M6G1 | M1G1 | 3 | 3 | -5.519 | 2.496 | 0.019 | 0.187 | ns |
| Acetate | M6G1 | M1G2 | 3 | 3 | -9.915 | 2.982 | 0.002 | 0.025 | * |
| Acetate | M6G1 | M6G2 | 3 | 3 | -0.485 | 2.249 | 0.671 | 1 | ns |
|  |  |  |  |  |  |  |  |  |  |
| Product | Ref strain | Strain | n1 | n2 | stat | df | p | p.adj | p.adj.signif |
| Ethanol | GS1151 | GS1152 | 3 | 3 | 0.426 | 3.004 | 0.699 | 1 | ns |
| Ethanol | GS1151 | I1G1 | 3 | 3 | -0.752 | 3.137 | 0.504 | 1 | ns |
| Ethanol | GS1151 | I1G2 | 3 | 3 | -0.903 | 2.816 | 0.437 | 1 | ns |
| Ethanol | GS1151 | I1S1 | 3 | 3 | -1.502 | 2.848 | 0.235 | 1 | ns |
| Ethanol | GS1151 | I1S2 | 3 | 3 | -0.879 | 3.593 | 0.434 | 1 | ns |
| Ethanol | GS1151 | I6G1 | 3 | 2 | 0.558 | 2.500 | 0.623 | 1 | ns |
| Ethanol | GS1151 | I6G2 | 3 | 3 | 0.683 | 2.438 | 0.554 | 1 | ns |
| Ethanol | GS1151 | M1G1 | 3 | 3 | -1.924 | 3.399 | 0.139 | 1 | ns |
| Ethanol | GS1151 | M1G2 | 3 | 3 | -2.836 | 2.752 | 0.073 | 0.802 | ns |
| Ethanol | GS1151 | M6G1 | 3 | 3 | 0.862 | 2.498 | 0.463 | 1 | ns |
| Ethanol | GS1151 | M6G2 | 3 | 3 | 0.636 | 2.049 | 0.589 | 1 | ns |
| Ethanol | I1G1 | GS1151 | 3 | 3 | 0.752 | 3.137 | 0.504 | 1 | ns |
| Ethanol | I1G1 | GS1152 | 3 | 3 | 0.890 | 2.334 | 0.456 | 1 | ns |
| Ethanol | I1G1 | I1G2 | 3 | 3 | -0.184 | 3.864 | 0.863 | 1 | ns |
| Ethanol | I1G1 | I1S1 | 3 | 3 | -1.094 | 3.891 | 0.337 | 1 | ns |
| Ethanol | I1G1 | I1S2 | 3 | 3 | -0.237 | 3.802 | 0.825 | 1 | ns |
| Ethanol | I1G1 | I6G1 | 3 | 2 | 1.375 | 1.538 | 0.335 | 1 | ns |
| Ethanol | I1G1 | I6G2 | 3 | 3 | 2.432 | 3.262 | 0.086 | 0.777 | ns |
| Ethanol | I1G1 | M1G1 | 3 | 3 | -1.675 | 3.930 | 0.171 | 1 | ns |
| Ethanol | I1G1 | M1G2 | 3 | 3 | -3.144 | 3.799 | 0.037 | 0.41 | ns |
| Ethanol | I1G1 | M6G1 | 3 | 3 | 2.683 | 3.393 | 0.066 | 0.656 | ns |
| Ethanol | I1G1 | M6G2 | 3 | 3 | 2.637 | 2.158 | 0.11 | 0.88 | ns |
| Ethanol | I6G1 | GS1151 | 2 | 3 | -0.558 | 2.500 | 0.623 | 1 | ns |
| Ethanol | I6G1 | GS1152 | 2 | 3 | 0.044 | 2.894 | 0.968 | 1 | ns |
| Ethanol | I6G1 | I1G1 | 2 | 3 | -1.375 | 1.538 | 0.335 | 1 | ns |
| Ethanol | I6G1 | I1G2 | 2 | 3 | -1.535 | 1.368 | 0.316 | 1 | ns |
| Ethanol | I6G1 | I1S1 | 2 | 3 | -2.090 | 1.384 | 0.226 | 1 | ns |
| Ethanol | I6G1 | I1S2 | 2 | 3 | -1.462 | 1.845 | 0.291 | 1 | ns |
| Ethanol | I6G1 | I6G2 | 2 | 3 | -0.093 | 1.189 | 0.939 | 1 | ns |
| Ethanol | I6G1 | M1G1 | 2 | 3 | -2.456 | 1.701 | 0.155 | 1 | ns |
| Ethanol | I6G1 | M1G2 | 2 | 3 | -3.332 | 1.336 | 0.134 | 1 | ns |
| Ethanol | I6G1 | M6G1 | 2 | 3 | 0.077 | 1.216 | 0.949 | 1 | ns |
| Ethanol | I6G1 | M6G2 | 2 | 3 | -0.170 | 1.021 | 0.892 | 1 | ns |
| Ethanol | I1S1 | GS1151 | 3 | 3 | 1.502 | 2.848 | 0.235 | 1 | ns |
| Ethanol | I1S1 | GS1152 | 3 | 3 | 1.303 | 2.239 | 0.31 | 1 | ns |
| Ethanol | I1S1 | I1G1 | 3 | 3 | 1.094 | 3.891 | 0.337 | 1 | ns |
| Ethanol | I1S1 | I1G2 | 3 | 3 | 1.009 | 3.998 | 0.37 | 1 | ns |
| Ethanol | I1S1 | I1S2 | 3 | 3 | 0.691 | 3.494 | 0.532 | 1 | ns |
| Ethanol | I1S1 | I6G1 | 3 | 2 | 2.090 | 1.384 | 0.226 | 1 | ns |
| Ethanol | I1S1 | I6G2 | 3 | 3 | 4.123 | 3.596 | 0.018 | 0.181 | ns |
| Ethanol | I1S1 | M1G1 | 3 | 3 | -0.783 | 3.681 | 0.481 | 1 | ns |
| Ethanol | I1S1 | M1G2 | 3 | 3 | -2.227 | 3.983 | 0.09 | 0.722 | ns |
| Ethanol | I1S1 | M6G1 | 3 | 3 | 4.360 | 3.718 | 0.014 | 0.156 | ns |
| Ethanol | I1S1 | M6G2 | 3 | 3 | 4.747 | 2.221 | 0.034 | 0.304 | ns |
| Ethanol | M1G1 | GS1151 | 3 | 3 | 1.924 | 3.399 | 0.139 | 1 | ns |
| Ethanol | M1G1 | GS1152 | 3 | 3 | 1.580 | 2.434 | 0.233 | 1 | ns |
| Ethanol | M1G1 | I1G1 | 3 | 3 | 1.675 | 3.930 | 0.171 | 1 | ns |
| Ethanol | M1G1 | I1G2 | 3 | 3 | 1.634 | 3.642 | 0.185 | 1 | ns |
| Ethanol | M1G1 | I1S1 | 3 | 3 | 0.783 | 3.681 | 0.481 | 1 | ns |
| Ethanol | M1G1 | I1S2 | 3 | 3 | 1.270 | 3.962 | 0.273 | 1 | ns |
| Ethanol | M1G1 | I6G1 | 3 | 2 | 2.456 | 1.701 | 0.155 | 1 | ns |
| Ethanol | M1G1 | I6G2 | 3 | 3 | 4.168 | 3.011 | 0.025 | 0.25 | ns |
| Ethanol | M1G1 | M1G2 | 3 | 3 | -1.052 | 3.557 | 0.359 | 1 | ns |
| Ethanol | M1G1 | M6G1 | 3 | 3 | 4.373 | 3.131 | 0.02 | 0.222 | ns |
| Ethanol | M1G1 | M6G2 | 3 | 3 | 4.507 | 2.121 | 0.041 | 0.37 | ns |
| Ethanol | M6G1 | GS1151 | 3 | 3 | -0.862 | 2.498 | 0.463 | 1 | ns |
| Ethanol | M6G1 | GS1152 | 3 | 3 | 0.005 | 2.136 | 0.997 | 1 | ns |
| Ethanol | M6G1 | I1G1 | 3 | 3 | -2.683 | 3.393 | 0.066 | 0.459 | ns |
| Ethanol | M6G1 | I1G2 | 3 | 3 | -3.276 | 3.755 | 0.034 | 0.269 | ns |
| Ethanol | M6G1 | I1S1 | 3 | 3 | -4.360 | 3.718 | 0.014 | 0.142 | ns |
| Ethanol | M6G1 | I1S2 | 3 | 3 | -2.521 | 2.957 | 0.087 | 0.524 | ns |
| Ethanol | M6G1 | I6G1 | 3 | 2 | -0.077 | 1.216 | 0.949 | 1 | ns |
| Ethanol | M6G1 | I6G2 | 3 | 3 | -0.401 | 3.983 | 0.709 | 1 | ns |
| Ethanol | M6G1 | M1G1 | 3 | 3 | -4.373 | 3.131 | 0.02 | 0.182 | ns |
| Ethanol | M6G1 | M1G2 | 3 | 3 | -7.080 | 3.826 | 0.002 | 0.027 | * |
| Ethanol | M6G1 | M6G2 | 3 | 3 | -0.739 | 2.386 | 0.526 | 1 | ns |
|  |  |  |  |  |  |  |  |  |  |
| Product | Ref strain | Strain | n1 | n2 | stat | df | p | p.adj | p.adj.signif |
| Biomass Yield | GS1151 | GS1152 | 3 | 3 | -0.208 | 3.415 | 0.847 | 1 | ns |
| Biomass Yield | GS1151 | I1G1 | 3 | 3 | -0.300 | 3.761 | 0.78 | 1 | ns |
| Biomass Yield | GS1151 | I1G2 | 3 | 3 | 0.303 | 3.997 | 0.777 | 1 | ns |
| Biomass Yield | GS1151 | I1S1 | 3 | 3 | 0.388 | 2.283 | 0.731 | 1 | ns |
| Biomass Yield | GS1151 | I1S2 | 3 | 3 | 0.171 | 2.253 | 0.878 | 1 | ns |
| Biomass Yield | GS1151 | I6G1 | 3 | 3 | -0.197 | 3.719 | 0.854 | 1 | ns |
| Biomass Yield | GS1151 | I6G2 | 3 | 3 | 0.153 | 3.346 | 0.887 | 1 | ns |
| Biomass Yield | GS1151 | M1G1 | 3 | 3 | 0.758 | 2.012 | 0.527 | 1 | ns |
| Biomass Yield | GS1151 | M1G2 | 3 | 3 | 0.772 | 2.306 | 0.512 | 1 | ns |
| Biomass Yield | GS1151 | M6G1 | 3 | 3 | 0.590 | 2.000 | 0.615 | 1 | ns |
| Biomass Yield | GS1151 | M6G2 | 3 | 3 | 0.340 | 2.119 | 0.764 | 1 | ns |
| Biomass Yield | I1G1 | GS1151 | 3 | 3 | 0.300 | 3.761 | 0.78 | 1 | ns |
| Biomass Yield | I1G1 | GS1152 | 3 | 3 | 0.167 | 2.934 | 0.878 | 1 | ns |
| Biomass Yield | I1G1 | I1G2 | 3 | 3 | 0.560 | 3.806 | 0.607 | 1 | ns |
| Biomass Yield | I1G1 | I1S1 | 3 | 3 | 0.675 | 2.169 | 0.564 | 1 | ns |
| Biomass Yield | I1G1 | I1S2 | 3 | 3 | 0.506 | 2.152 | 0.66 | 1 | ns |
| Biomass Yield | I1G1 | I6G1 | 3 | 3 | 0.163 | 3.219 | 0.88 | 1 | ns |
| Biomass Yield | I1G1 | I6G2 | 3 | 3 | 0.467 | 2.878 | 0.674 | 1 | ns |
| Biomass Yield | I1G1 | M1G1 | 3 | 3 | 0.965 | 2.007 | 0.436 | 1 | ns |
| Biomass Yield | I1G1 | M1G2 | 3 | 3 | 0.976 | 2.184 | 0.425 | 1 | ns |
| Biomass Yield | I1G1 | M6G1 | 3 | 3 | 0.835 | 2.000 | 0.492 | 1 | ns |
| Biomass Yield | I1G1 | M6G2 | 3 | 3 | 0.640 | 2.071 | 0.586 | 1 | ns |
| Biomass Yield | I6G1 | GS1151 | 3 | 3 | 0.197 | 3.719 | 0.854 | 1 | ns |
| Biomass Yield | I6G1 | GS1152 | 3 | 3 | -0.002 | 3.904 | 0.999 | 1 | ns |
| Biomass Yield | I6G1 | I1G1 | 3 | 3 | -0.163 | 3.219 | 0.88 | 1 | ns |
| Biomass Yield | I6G1 | I1G2 | 3 | 3 | 0.534 | 3.669 | 0.624 | 1 | ns |
| Biomass Yield | I6G1 | I1S1 | 3 | 3 | 0.810 | 2.491 | 0.488 | 1 | ns |
| Biomass Yield | I6G1 | I1S2 | 3 | 3 | 0.531 | 2.442 | 0.64 | 1 | ns |
| Biomass Yield | I6G1 | I6G2 | 3 | 3 | 0.436 | 3.860 | 0.686 | 1 | ns |
| Biomass Yield | I6G1 | M1G1 | 3 | 3 | 1.329 | 2.022 | 0.314 | 1 | ns |
| Biomass Yield | I6G1 | M1G2 | 3 | 3 | 1.303 | 2.531 | 0.299 | 1 | ns |
| Biomass Yield | I6G1 | M6G1 | 3 | 3 | 1.109 | 2.000 | 0.383 | 1 | ns |
| Biomass Yield | I6G1 | M6G2 | 3 | 3 | 0.765 | 2.208 | 0.518 | 1 | ns |
| Biomass Yield | I1S1 | GS1151 | 3 | 3 | -0.388 | 2.283 | 0.731 | 1 | ns |
| Biomass Yield | I1S1 | GS1152 | 3 | 3 | -0.932 | 2.665 | 0.428 | 1 | ns |
| Biomass Yield | I1S1 | I1G1 | 3 | 3 | -0.675 | 2.169 | 0.564 | 1 | ns |
| Biomass Yield | I1S1 | I1G2 | 3 | 3 | 0.032 | 2.267 | 0.977 | 1 | ns |
| Biomass Yield | I1S1 | I1S2 | 3 | 3 | -0.614 | 3.988 | 0.573 | 1 | ns |
| Biomass Yield | I1S1 | I6G1 | 3 | 3 | -0.810 | 2.491 | 0.488 | 1 | ns |
| Biomass Yield | I1S1 | I6G2 | 3 | 3 | -0.327 | 2.710 | 0.767 | 1 | ns |
| Biomass Yield | I1S1 | M1G1 | 3 | 3 | 1.314 | 2.174 | 0.31 | 1 | ns |
| Biomass Yield | I1S1 | M1G2 | 3 | 3 | 1.038 | 3.994 | 0.358 | 1 | ns |
| Biomass Yield | I1S1 | M6G1 | 3 | 3 | 0.708 | 2.004 | 0.552 | 1 | ns |
| Biomass Yield | I1S1 | M6G2 | 3 | 3 | -0.176 | 3.423 | 0.87 | 1 | ns |
| Biomass Yield | M1G1 | GS1151 | 3 | 3 | -0.758 | 2.012 | 0.527 | 1 | ns |
| Biomass Yield | M1G1 | GS1152 | 3 | 3 | -1.558 | 2.030 | 0.258 | 1 | ns |
| Biomass Yield | M1G1 | I1G1 | 3 | 3 | -0.965 | 2.007 | 0.436 | 1 | ns |
| Biomass Yield | M1G1 | I1G2 | 3 | 3 | -0.315 | 2.012 | 0.783 | 1 | ns |
| Biomass Yield | M1G1 | I1S1 | 3 | 3 | -1.314 | 2.174 | 0.31 | 1 | ns |
| Biomass Yield | M1G1 | I1S2 | 3 | 3 | -2.257 | 2.195 | 0.141 | 1 | ns |
| Biomass Yield | M1G1 | I6G1 | 3 | 3 | -1.329 | 2.022 | 0.314 | 1 | ns |
| Biomass Yield | M1G1 | I6G2 | 3 | 3 | -0.927 | 2.032 | 0.451 | 1 | ns |
| Biomass Yield | M1G1 | M1G2 | 3 | 3 | 0.147 | 2.161 | 0.896 | 1 | ns |
| Biomass Yield | M1G1 | M6G1 | 3 | 3 | -3.000 | 2.091 | 0.09 | 0.995 | ns |
| Biomass Yield | M1G1 | M6G2 | 3 | 3 | -2.285 | 2.414 | 0.128 | 1 | ns |
| Biomass Yield | M6G1 | GS1151 | 3 | 3 | -0.590 | 2.000 | 0.615 | 1 | ns |
| Biomass Yield | M6G1 | GS1152 | 3 | 3 | -1.301 | 2.001 | 0.323 | 1 | ns |
| Biomass Yield | M6G1 | I1G1 | 3 | 3 | -0.835 | 2.000 | 0.492 | 1 | ns |
| Biomass Yield | M6G1 | I1G2 | 3 | 3 | -0.151 | 2.000 | 0.894 | 1 | ns |
| Biomass Yield | M6G1 | I1S1 | 3 | 3 | -0.708 | 2.004 | 0.552 | 1 | ns |
| Biomass Yield | M6G1 | I1S2 | 3 | 3 | -1.640 | 2.004 | 0.242 | 1 | ns |
| Biomass Yield | M6G1 | I6G1 | 3 | 3 | -1.109 | 2.000 | 0.383 | 1 | ns |
| Biomass Yield | M6G1 | I6G2 | 3 | 3 | -0.659 | 2.001 | 0.578 | 1 | ns |
| Biomass Yield | M6G1 | M1G1 | 3 | 3 | 3.000 | 2.091 | 0.09 | 0.995 | ns |
| Biomass Yield | M6G1 | M1G2 | 3 | 3 | 0.758 | 2.004 | 0.527 | 1 | ns |
| Biomass Yield | M6G1 | M6G2 | 3 | 3 | -1.419 | 2.010 | 0.291 | 1 | ns |
|  |  |  |  |  |  |  |  |  |  |
| Product | Ref strain | Strain | n1 | n2 | stat | df | p | p.adj | p.adj.signif |
| CO2 Yield | GS1151 | GS1152 | 3 | 3 | 0.405 | 3.407 | 0.71 | 1 | ns |
| CO2 Yield | GS1151 | I1G1 | 3 | 3 | 0.105 | 3.877 | 0.921 | 1 | ns |
| CO2 Yield | GS1151 | I1G2 | 3 | 3 | -0.415 | 3.521 | 0.702 | 1 | ns |
| CO2 Yield | GS1151 | I1S1 | 3 | 3 | -0.235 | 2.167 | 0.835 | 1 | ns |
| CO2 Yield | GS1151 | I1S2 | 3 | 3 | 0.096 | 2.128 | 0.932 | 1 | ns |
| CO2 Yield | GS1151 | I6G1 | 3 | 3 | 0.376 | 3.228 | 0.73 | 1 | ns |
| CO2 Yield | GS1151 | I6G2 | 3 | 3 | -0.008 | 2.869 | 0.994 | 1 | ns |
| CO2 Yield | GS1151 | M1G1 | 3 | 3 | -0.721 | 2.186 | 0.54 | 1 | ns |
| CO2 Yield | GS1151 | M1G2 | 3 | 3 | -0.796 | 2.330 | 0.499 | 1 | ns |
| CO2 Yield | GS1151 | M6G1 | 3 | 3 | -0.592 | 2.245 | 0.608 | 1 | ns |
| CO2 Yield | GS1151 | M6G2 | 3 | 3 | -0.214 | 2.080 | 0.85 | 1 | ns |
| CO2 Yield | I1G1 | GS1151 | 3 | 3 | -0.105 | 3.877 | 0.921 | 1 | ns |
| CO2 Yield | I1G1 | GS1152 | 3 | 3 | 0.233 | 3.060 | 0.83 | 1 | ns |
| CO2 Yield | I1G1 | I1G2 | 3 | 3 | -0.476 | 3.839 | 0.66 | 1 | ns |
| CO2 Yield | I1G1 | I1S1 | 3 | 3 | -0.333 | 2.117 | 0.769 | 1 | ns |
| CO2 Yield | I1G1 | I1S2 | 3 | 3 | -0.055 | 2.090 | 0.961 | 1 | ns |
| CO2 Yield | I1G1 | I6G1 | 3 | 3 | 0.204 | 2.906 | 0.852 | 1 | ns |
| CO2 Yield | I1G1 | I6G2 | 3 | 3 | -0.135 | 2.622 | 0.903 | 1 | ns |
| CO2 Yield | I1G1 | M1G1 | 3 | 3 | -0.742 | 2.130 | 0.531 | 1 | ns |
| CO2 Yield | I1G1 | M1G2 | 3 | 3 | -0.806 | 2.231 | 0.497 | 1 | ns |
| CO2 Yield | I1G1 | M6G1 | 3 | 3 | -0.633 | 2.172 | 0.587 | 1 | ns |
| CO2 Yield | I1G1 | M6G2 | 3 | 3 | -0.315 | 2.056 | 0.782 | 1 | ns |
| CO2 Yield | I6G1 | GS1151 | 3 | 3 | -0.376 | 3.228 | 0.73 | 1 | ns |
| CO2 Yield | I6G1 | GS1152 | 3 | 3 | 0.052 | 3.968 | 0.961 | 1 | ns |
| CO2 Yield | I6G1 | I1G1 | 3 | 3 | -0.204 | 2.906 | 0.852 | 1 | ns |
| CO2 Yield | I6G1 | I1G2 | 3 | 3 | -0.741 | 2.617 | 0.52 | 1 | ns |
| CO2 Yield | I6G1 | I1S1 | 3 | 3 | -1.089 | 2.482 | 0.37 | 1 | ns |
| CO2 Yield | I6G1 | I1S2 | 3 | 3 | -0.553 | 2.371 | 0.628 | 1 | ns |
| CO2 Yield | I6G1 | I6G2 | 3 | 3 | -0.589 | 3.845 | 0.589 | 1 | ns |
| CO2 Yield | I6G1 | M1G1 | 3 | 3 | -1.880 | 2.535 | 0.173 | 1 | ns |
| CO2 Yield | I6G1 | M1G2 | 3 | 3 | -1.936 | 2.915 | 0.151 | 1 | ns |
| CO2 Yield | I6G1 | M6G1 | 3 | 3 | -1.644 | 2.695 | 0.209 | 1 | ns |
| CO2 Yield | I6G1 | M6G2 | 3 | 3 | -1.082 | 2.231 | 0.382 | 1 | ns |
| CO2 Yield | I1S1 | GS1151 | 3 | 3 | 0.235 | 2.167 | 0.835 | 1 | ns |
| CO2 Yield | I1S1 | GS1152 | 3 | 3 | 1.071 | 2.404 | 0.38 | 1 | ns |
| CO2 Yield | I1S1 | I1G1 | 3 | 3 | 0.333 | 2.117 | 0.769 | 1 | ns |
| CO2 Yield | I1S1 | I1G2 | 3 | 3 | -0.335 | 2.077 | 0.768 | 1 | ns |
| CO2 Yield | I1S1 | I1S2 | 3 | 3 | 1.240 | 3.930 | 0.284 | 1 | ns |
| CO2 Yield | I1S1 | I6G1 | 3 | 3 | 1.089 | 2.482 | 0.37 | 1 | ns |
| CO2 Yield | I1S1 | I6G2 | 3 | 3 | 0.443 | 2.710 | 0.69 | 1 | ns |
| CO2 Yield | I1S1 | M1G1 | 3 | 3 | -1.672 | 3.988 | 0.17 | 1 | ns |
| CO2 Yield | I1S1 | M1G2 | 3 | 3 | -1.664 | 3.608 | 0.179 | 1 | ns |
| CO2 Yield | I1S1 | M6G1 | 3 | 3 | -1.150 | 3.861 | 0.316 | 1 | ns |
| CO2 Yield | I1S1 | M6G2 | 3 | 3 | 0.097 | 3.550 | 0.928 | 1 | ns |
| CO2 Yield | M1G1 | GS1151 | 3 | 3 | 0.721 | 2.186 | 0.54 | 1 | ns |
| CO2 Yield | M1G1 | GS1152 | 3 | 3 | 1.801 | 2.449 | 0.19 | 1 | ns |
| CO2 Yield | M1G1 | I1G1 | 3 | 3 | 0.742 | 2.130 | 0.531 | 1 | ns |
| CO2 Yield | M1G1 | I1G2 | 3 | 3 | 0.000 | 2.086 | 1 | 1 | ns |
| CO2 Yield | M1G1 | I1S1 | 3 | 3 | 1.672 | 3.988 | 0.17 | 1 | ns |
| CO2 Yield | M1G1 | I1S2 | 3 | 3 | 2.975 | 3.867 | 0.043 | 0.47 | ns |
| CO2 Yield | M1G1 | I6G1 | 3 | 3 | 1.880 | 2.535 | 0.173 | 1 | ns |
| CO2 Yield | M1G1 | I6G2 | 3 | 3 | 1.389 | 2.785 | 0.266 | 1 | ns |
| CO2 Yield | M1G1 | M1G2 | 3 | 3 | -0.251 | 3.708 | 0.815 | 1 | ns |
| CO2 Yield | M1G1 | M6G1 | 3 | 3 | 0.389 | 3.926 | 0.718 | 1 | ns |
| CO2 Yield | M1G1 | M6G2 | 3 | 3 | 2.022 | 3.443 | 0.125 | 1 | ns |
| CO2 Yield | M6G1 | GS1151 | 3 | 3 | 0.592 | 2.245 | 0.608 | 1 | ns |
| CO2 Yield | M6G1 | GS1152 | 3 | 3 | 1.587 | 2.586 | 0.225 | 1 | ns |
| CO2 Yield | M6G1 | I1G1 | 3 | 3 | 0.633 | 2.172 | 0.587 | 1 | ns |
| CO2 Yield | M6G1 | I1G2 | 3 | 3 | -0.086 | 2.113 | 0.939 | 1 | ns |
| CO2 Yield | M6G1 | I1S1 | 3 | 3 | 1.150 | 3.861 | 0.316 | 1 | ns |
| CO2 Yield | M6G1 | I1S2 | 3 | 3 | 2.311 | 3.639 | 0.088 | 0.973 | ns |
| CO2 Yield | M6G1 | I6G1 | 3 | 3 | 1.644 | 2.695 | 0.209 | 1 | ns |
| CO2 Yield | M6G1 | I6G2 | 3 | 3 | 1.115 | 3.005 | 0.346 | 1 | ns |
| CO2 Yield | M6G1 | M1G1 | 3 | 3 | -0.389 | 3.926 | 0.718 | 1 | ns |
| CO2 Yield | M6G1 | M1G2 | 3 | 3 | -0.574 | 3.913 | 0.597 | 1 | ns |
| CO2 Yield | M6G1 | M6G2 | 3 | 3 | 1.380 | 3.171 | 0.257 | 1 | ns |

**Supplementary Table 7**. Differentially expressed genes (RNA-seq), cutoff at adjusted p-value < 0.05 and |log_2_FC| >1, in I1S relative to I1G.

| Gene | Log_2_FC |
| --- | --- |
| MSTRG.3998.11 | -13.58 |
| MSTRG.3742.2 | -13.20 |
| MSTRG.3998.16 | 12.77 |
| MSTRG.2862.3 | 11.48 |
| MSTRG.1399.8 | -11.47 |
| MSTRG.3998.7 | -11.34 |
| MSTRG.1399.5 | -10.75 |
| MSTRG.2136.4 | -10.13 |
| MSTRG.2633.24 | -10.10 |
| XM_002492742.1 | 9.09 |
| MSTRG.1399.1 | -7.96 |
| MSTRG.1399.2 | -7.31 |
| MSTRG.1399.3 | -6.87 |
| MSTRG.3742.5 | -6.61 |
| MSTRG.1399.4 | -6.14 |
| XM_002492686.1 | -5.64 |
| XM_002490674.1 | -5.27 |
| XM_002490676.1 | -5.11 |
| XM_002491313.1 | 5.01 |
| XM_002492948.1 | -4.97 |
| XM_002494335.1 | -4.90 |
| XM_002493617.1 | -4.75 |
| XM_002492687.1 | -4.67 |
| XM_002489868.1 | 3.99 |
| XM_002492206.1 | -3.94 |
| XM_002489890.1 | -3.73 |
| XM_002489408.1 | 3.67 |
| XM_002490840.1 | -3.54 |
| MSTRG.3952.1 | 3.38 |
| Insulin | 3.35 |
| XM_002490229.1 | 3.22 |
| XM_002490320.1 | -3.16 |
| MSTRG.135.1 | -3.08 |
| XM_002490827.1 | -3.05 |
| XM_002493440.1 | 3.00 |
| XM_002489947.1 | 2.86 |
| XM_002490001.1 | -2.81 |
| XM_002492332.1 | 2.67 |
| XM_002491521.1 | -2.65 |
| XM_002490680.1 | -2.64 |
| XM_002492354.1 | -2.54 |
| XM_002492152.1 | -2.47 |
| XM_002494291.1 | -2.39 |
| XM_002494336.1 | 2.31 |
| XM_002491035.1 | -2.30 |
| MSTRG.2136.5 | -2.29 |
| XM_002493203.1 | 2.28 |
| XM_002492183.1 | 2.27 |
| XM_002490287.1 | 2.26 |
| XM_002490567.1 | -2.21 |
| XM_002490475.1 | -2.18 |
| XM_002491358.1 | -2.13 |
| XM_002492843.1 | -2.11 |
| XM_002490110.1 | 2.03 |
| XM_002490765.1 | 2.00 |
| XM_002490311.1 | -1.99 |
| XM_002493049.1 | -1.99 |
| XM_002491539.1 | -1.98 |
| XM_002491129.1 | -1.97 |
| XM_002490494.1 | -1.95 |
| XM_002492877.1 | -1.95 |
| MSTRG.2621.1 | 1.94 |
| XM_002489983.1 | -1.94 |
| XM_002492526.1 | -1.90 |
| XM_002492527.1 | -1.90 |
| XM_002490661.1 | -1.85 |
| XM_002491634.1 | 1.82 |
| XM_002493052.1 | 1.81 |
| XM_002492351.1 | 1.77 |
| XM_002494294.1 | -1.77 |
| XM_002493008.1 | -1.76 |
| MSTRG.612.1 | -1.76 |
| XM_002490459.1 | -1.76 |
| XM_002491799.1 | -1.76 |
| XM_002492591.1 | -1.72 |
| XM_002492344.1 | -1.69 |
| XM_002493287.1 | -1.67 |
| XM_002493680.1 | -1.67 |
| XM_002490373.1 | -1.65 |
| XM_002493878.1 | -1.64 |
| XM_002493251.1 | -1.63 |
| XM_002489314.1 | -1.62 |
| XM_002493245.1 | -1.61 |
| XM_002491640.1 | -1.60 |
| XM_002491349.1 | -1.58 |
| XM_002489864.1 | -1.57 |
| XM_002492461.1 | -1.57 |
| MSTRG.618.1 | -1.57 |
| XM_002490194.1 | 1.56 |
| XM_002492340.1 | -1.56 |
| XM_002494235.1 | -1.55 |
| MSTRG.1239.1 | 1.54 |
| XM_002490769.1 | -1.53 |
| XM_002489945.1 | -1.53 |
| MSTRG.4024.3 | 1.52 |
| XM_002493047.1 | 1.51 |
| XM_002493033.1 | -1.51 |
| XM_002490137.1 | 1.49 |
| XM_002492708.1 | 1.48 |
| XM_002491822.1 | -1.47 |
| XM_002490496.1 | 1.47 |
| XM_002490142.1 | -1.46 |
| MSTRG.1434.1 | 1.46 |
| XM_002490662.1 | -1.45 |
| XM_002491881.1 | -1.45 |
| XM_002492717.1 | 1.44 |
| XM_002494021.1 | -1.43 |
| XM_002491386.1 | 1.43 |
| XM_002494106.1 | -1.43 |
| XM_002490155.1 | -1.41 |
| MSTRG.2136.3 | -1.41 |
| XM_002489969.1 | -1.41 |
| XM_002493372.1 | -1.40 |
| MSTRG.2644.2 | -1.39 |
| XM_002489955.1 | -1.38 |
| XM_002489366.1 | 1.37 |
| XM_002490457.1 | -1.36 |
| XM_002493625.1 | -1.36 |
| XM_002490312.1 | -1.35 |
| XM_002491843.1 | -1.33 |
| XM_002490593.1 | 1.33 |
| XM_002490018.1 | 1.33 |
| XM_002493317.1 | -1.33 |
| XM_002493441.1 | 1.32 |
| XM_002489514.1 | -1.30 |
| XM_002493752.1 | 1.30 |
| XM_002492134.1 | -1.29 |
| XM_002490071.1 | 1.28 |
| XM_002489824.1 | -1.28 |
| XM_002491629.1 | -1.28 |
| XM_002491528.1 | -1.28 |
| XM_002492818.1 | -1.27 |
| XM_002489720.1 | -1.27 |
| XM_002490628.1 | -1.26 |
| XM_002489556.1 | 1.26 |
| XM_002491944.1 | -1.25 |
| XM_002489978.1 | 1.25 |
| XM_002489403.1 | 1.25 |
| XM_002489731.1 | -1.24 |
| XM_002493972.1 | -1.24 |
| XM_002490851.1 | -1.24 |
| XM_002492256.1 | -1.24 |
| XM_002490364.1 | -1.23 |
| XM_002489807.1 | -1.23 |
| XM_002489694.1 | -1.22 |
| XM_002493663.1 | 1.22 |
| XM_002492525.1 | -1.21 |
| XM_002492163.1 | -1.21 |
| XM_002493347.1 | 1.21 |
| XM_002491385.1 | 1.21 |
| XM_002493946.1 | 1.20 |
| XM_002493983.1 | 1.20 |
| XM_002492408.1 | -1.19 |
| XM_002489817.1 | 1.19 |
| XM_002494019.1 | -1.18 |
| XM_002491821.1 | -1.18 |
| XM_002493896.1 | 1.18 |
| XM_002493080.1 | -1.18 |
| XM_002494151.1 | -1.18 |
| XM_002492924.1 | -1.18 |
| XM_002492352.1 | -1.17 |
| XM_002489790.1 | -1.17 |
| XM_002490843.1 | -1.17 |
| XM_002493569.1 | -1.16 |
| XM_002493714.1 | -1.16 |
| MSTRG.2203.6 | -1.15 |
| XM_002492749.1 | 1.15 |
| XM_002489994.1 | -1.15 |
| XM_002492187.1 | -1.15 |
| MSTRG.2817.1 | -1.15 |
| XM_002492772.1 | -1.15 |
| XM_002493511.1 | -1.15 |
| XM_002492018.1 | -1.15 |
| XM_002490636.1 | -1.15 |
| XM_002493368.1 | -1.15 |
| XM_002492406.1 | -1.14 |
| XM_002491046.1 | -1.14 |
| XM_002493276.1 | -1.14 |
| XM_002492535.1 | 1.14 |
| XM_002490805.1 | -1.13 |
| XM_002493046.1 | -1.11 |
| XM_002491882.1 | 1.11 |
| XM_002492716.1 | 1.11 |
| XM_002490025.1 | 1.11 |
| XM_002492020.1 | 1.11 |
| XM_002490522.1 | -1.11 |
| XM_002489313.1 | -1.10 |
| XM_002490797.1 | -1.10 |
| MSTRG.2633.7 | -1.09 |
| XM_002493991.1 | -1.09 |
| XM_002489589.1 | -1.09 |
| XM_002490751.1 | -1.09 |
| XM_002490087.1 | -1.09 |
| XM_002493551.1 | -1.09 |
| XM_002493871.1 | -1.08 |
| XM_002492180.1 | -1.08 |
| XM_002491027.1 | 1.08 |
| XM_002490337.1 | 1.08 |
| XM_002494240.1 | -1.08 |
| XM_002490335.1 | 1.08 |
| XM_002494026.1 | -1.08 |
| XM_002492192.1 | -1.07 |
| XM_002491768.1 | 1.07 |
| XM_002490331.1 | 1.06 |
| XM_002491632.1 | 1.06 |
| XM_002491348.1 | 1.06 |
| XM_002491006.1 | 1.05 |
| XM_002491330.1 | 1.05 |
| MSTRG.1380.1 | -1.05 |
| XM_002490613.1 | -1.05 |
| XM_002493293.1 | -1.05 |
| XM_002491749.1 | -1.05 |
| XM_002490524.1 | -1.05 |
| XM_002493083.1 | -1.05 |
| XM_002489352.1 | -1.04 |
| MSTRG.1247.1 | -1.03 |
| XM_002492637.1 | -1.03 |
| XM_002491259.1 | -1.03 |
| XM_002494231.1 | -1.03 |
| XM_002492882.1 | 1.03 |
| XM_002492718.1 | 1.03 |
| XM_002493011.1 | 1.02 |
| XM_002491260.1 | -1.02 |
| XM_002489422.1 | -1.02 |
| XM_002492986.1 | 1.02 |
| XM_002489879.1 | -1.02 |
| XM_002491707.1 | -1.01 |
| XM_002490285.1 | -1.01 |
| XM_002493044.1 | -1.01 |
| XM_002490409.1 | -1.01 |
| XM_002494226.1 | -1.01 |
| XM_002492164.1 | -1.01 |
| XM_002493396.1 | -1.01 |
| XM_002492287.1 | 1.00 |
| XM_002493025.1 | -1.00 |
| XM_002490761.1 | -1.00 |
| XM_002492964.1 | -1.00 |

**Supplementary Table 8**. Differentially expressed genes (RNA-seq), cutoff at adjusted p-value < 0.05 and |log_2_FC| >1, in I6G relative to I1G.

| Gene | Log_2_FC |
| --- | --- |
| MSTRG.3742.5 | -12.02 |
| XM_002490674.1 | -5.81 |
| XM_002490676.1 | -5.07 |
| XM_002494335.1 | -4.77 |
| MSTRG.3952.1 | 4.23 |
| XM_002489890.1 | -3.53 |
| XM_002489868.1 | 3.34 |
| XM_002490840.1 | -2.98 |
| Insulin | 2.90 |
| XM_002493440.1 | 2.41 |
| XM_002492206.1 | -1.98 |
| XM_002490229.1 | 1.88 |
| XM_002492183.1 | 1.84 |
| XM_002490001.1 | -1.73 |
| XM_002492843.1 | -1.73 |
| XM_002492288.1 | -1.72 |
| XM_002494291.1 | -1.69 |
| XM_002493203.1 | 1.69 |
| XM_002492332.1 | 1.61 |
| MSTRG.2633.4 | -1.42 |
| MSTRG.2621.1 | 1.38 |
| XM_002491129.1 | -1.36 |
| XM_002489955.1 | -1.34 |
| XM_002492134.1 | -1.18 |
| XM_002490110.1 | 1.17 |
| XM_002492256.1 | -1.15 |
| XM_002489807.1 | -1.14 |
| XM_002489313.1 | -1.08 |
| XM_002492018.1 | -1.07 |
| MSTRG.1239.1 | 1.07 |
| XM_002494106.1 | -1.06 |
| XM_002490194.1 | 1.02 |

**Supplementary Table 9**. Differentially expressed genes (RNA-seq), cutoff at adjusted p-value < 0.05 and log_2_FC >|1|, in M6G relative to M1G.

| Gene | Log_2_FC |
| --- | --- |
| MSTRG.4283.2 | -11.79 |
| XM_002492879.1 | -5.06 |
| XM_002493524.1 | 5.03 |
| XM_002492008.1 | -3.41 |
| XM_002490320.1 | -3.00 |
| MSTRG.2666.1 | 2.86 |
| XM_002490229.1 | 2.47 |
| Mambalgin | 2.25 |
| XM_002489366.1 | 2.00 |
| XM_002492110.1 | -1.43 |
| XM_002491632.1 | 1.19 |
| XM_002491122.1 | -1.17 |
| XM_002493252.1 | 1.03 |
| XM_002491314.1 | -1.02 |
| XM_002489556.1 | 1.00 |
| XM_002491882.1 | 0.97 |
| XM_002490496.1 | 0.95 |
| XM_002492137.1 | -0.90 |
| XM_002492843.1 | -0.87 |
| XM_002493202.1 | -0.83 |
| XM_002490567.1 | -0.81 |
| XM_002491348.1 | 0.80 |
| XM_002490106.1 | 0.79 |
| XM_002489824.1 | -0.77 |
| XM_002491828.1 | 0.77 |
| XM_002489364.1 | 0.77 |
| XM_002493167.1 | -0.76 |
| XM_002491543.1 | 0.76 |
| XM_002490495.1 | 0.73 |
| XM_002490662.1 | -0.73 |
| XM_002489353.1 | 0.71 |
| XM_002492319.1 | -0.70 |
| XM_002492287.1 | 0.69 |
| XM_002493934.1 | -0.66 |
| XM_002491768.1 | 0.65 |
| XM_002493858.1 | -0.63 |
| XM_002493972.1 | -0.63 |
| XM_002494242.1 | 0.62 |
| XM_002492708.1 | 0.61 |
| MSTRG.4283.1 | 0.61 |
| XM_002490068.1 | 0.59 |
| XM_002490416.1 | -0.58 |
| XM_002489731.1 | -0.58 |
| XM_002490139.1 | -0.56 |
| XM_002492332.1 | 0.56 |
| XM_002493909.1 | 0.55 |
| XM_002491989.1 | 0.55 |
| XM_002492516.1 | 0.55 |
| XM_002490503.1 | -0.55 |
| XM_002491601.1 | 0.54 |
| XM_002491394.1 | 0.54 |
| XM_002493169.1 | -0.54 |
| XM_002490768.1 | -0.54 |
| XM_002493282.1 | -0.53 |
| XM_002493728.1 | 0.53 |
| XM_002491330.1 | 0.53 |
| XM_002493525.1 | 0.52 |
| XM_002491512.1 | 0.52 |
| XM_002493280.1 | 0.52 |
| XM_002491772.1 | 0.51 |
| XM_002491436.1 | 0.51 |
| XM_002493165.1 | -0.50 |
| XM_002492584.1 | 0.50 |
| XM_002490135.1 | 0.50 |
| XM_002490214.1 | 0.50 |
| XM_002489950.1 | 0.50 |
| XM_002489613.1 | 0.49 |
| XM_002491161.1 | -0.48 |
| XM_002489438.1 | -0.47 |
| XM_002490093.1 | 0.47 |
| XM_002490663.1 | -0.47 |
| XM_002493104.1 | 0.47 |
| XM_002491683.1 | 0.46 |
| XM_002491573.1 | 0.46 |
| XM_002494144.1 | 0.46 |
| XM_002492114.1 | -0.46 |
| XM_002489928.1 | 0.46 |
| XM_002491825.1 | 0.46 |
| XM_002492637.1 | -0.45 |
| XM_002493131.1 | -0.45 |
| XM_002493364.1 | -0.45 |
| XM_002493771.1 | 0.44 |
| XM_002489988.1 | 0.44 |
| XM_002490351.1 | 0.43 |
| XM_002491160.1 | 0.43 |
| XM_002490308.1 | 0.43 |
| XM_002494164.1 | 0.43 |
| XM_002492207.1 | -0.43 |
| XM_002493879.1 | 0.43 |
| XM_002493203.1 | 0.42 |
| XM_002493826.1 | -0.42 |
| XM_002489707.1 | -0.42 |
| XM_002493011.1 | 0.42 |
| XM_002492291.1 | -0.42 |
| XM_002493479.1 | -0.41 |
| XM_002492105.1 | 0.41 |
| XM_002489708.1 | -0.41 |
| XM_002494296.1 | 0.41 |
| XM_002489823.1 | 0.41 |
| XM_002493878.1 | -0.40 |
| XM_002491453.1 | -0.40 |
| XM_002494284.1 | -0.40 |
| XM_002489603.1 | 0.40 |
| XM_002489816.1 | -0.40 |
| XM_002490447.1 | -0.39 |
| XM_002494153.1 | -0.39 |
| XM_002490572.1 | 0.39 |
| XM_002492479.1 | -0.39 |
| XM_002493917.1 | -0.39 |
| XM_002493421.1 | 0.38 |
| MSTRG.4159.2 | 0.38 |
| XM_002491926.1 | -0.38 |
| XM_002489313.1 | -0.37 |
| XM_002492095.1 | 0.37 |
| XM_002493816.1 | -0.37 |
| XM_002492136.1 | 0.37 |
| XM_002490011.1 | -0.37 |
| XM_002489437.1 | 0.36 |
| XM_002491339.1 | 0.36 |
| XM_002492682.1 | 0.35 |
| XM_002492632.1 | 0.35 |
| XM_002490274.1 | 0.35 |
| XM_002489942.1 | 0.35 |
| XM_002490667.1 | 0.35 |
| XM_002490405.1 | 0.35 |
| XM_002491461.1 | 0.35 |
| XM_002494181.1 | 0.34 |
| XM_002491351.1 | 0.34 |
| XM_002494074.1 | 0.34 |
| XM_002493704.1 | -0.34 |
| XM_002493243.1 | 0.34 |
| MSTRG.2731.1 | 0.34 |
| XM_002494020.1 | -0.34 |
| XM_002492688.1 | -0.33 |
| XM_002491426.1 | -0.33 |
| XM_002494067.1 | 0.33 |
| XM_002491014.1 | -0.33 |
| XM_002491332.1 | 0.33 |
| XM_002492058.1 | 0.33 |
| XM_002490061.1 | 0.33 |
| XM_002491942.1 | 0.33 |
| XM_002491771.1 | 0.33 |
| XM_002489344.1 | 0.33 |
| XM_002490885.1 | -0.32 |
| XM_002489805.1 | -0.32 |
| XM_002491489.1 | 0.32 |
| XM_002491844.1 | 0.32 |
| XM_002494091.1 | 0.32 |
| MSTRG.446.1 | 0.32 |
| XM_002491469.1 | 0.32 |
| XM_002493333.1 | -0.32 |
| XM_002494115.1 | 0.32 |
| XM_002492480.1 | -0.31 |
| XM_002491174.1 | 0.31 |
| XM_002490185.1 | 0.31 |
| XM_002490672.1 | 0.31 |
| MSTRG.2619.1 | 0.31 |
| XM_002493297.1 | 0.31 |
| XM_002494324.1 | -0.31 |
| XM_002492601.1 | -0.31 |
| XM_002493487.1 | 0.31 |
| XM_002493100.1 | 0.31 |
| XM_002491439.1 | 0.30 |
| XM_002491759.1 | 0.30 |
| XM_002493156.1 | 0.30 |
| XM_002491765.1 | 0.30 |
| XM_002489876.1 | -0.30 |
| XM_002490149.1 | 0.30 |
| XM_002490988.1 | 0.30 |
| XM_002493371.1 | -0.30 |
| XM_002489755.1 | 0.30 |
| XM_002494203.1 | 0.30 |
| XM_002493263.1 | -0.30 |
| XM_002491750.1 | 0.29 |
| XM_002492071.1 | 0.29 |
| XM_002493693.1 | 0.29 |
| XM_002490291.1 | -0.29 |
| XM_002490618.1 | -0.29 |
| XM_002493758.1 | 0.29 |
| XM_002491456.1 | 0.29 |
| XM_002492506.1 | 0.29 |
| MSTRG.3548.1 | 0.29 |
| XM_002492094.1 | 0.29 |
| XM_002493161.1 | -0.29 |
| XM_002492943.1 | -0.28 |
| XM_002492707.1 | -0.28 |
| XM_002493090.1 | 0.28 |
| XM_002493269.1 | 0.28 |
| XM_002491832.1 | 0.28 |
| XM_002491457.1 | -0.28 |
| XM_002491310.1 | 0.28 |
| XM_002489971.1 | 0.28 |
| XM_002492191.1 | -0.28 |
| XM_002490074.1 | 0.28 |
| XM_002491933.1 | 0.28 |
| XM_002494030.1 | -0.28 |
| XM_002493385.1 | 0.27 |
| XM_002489382.1 | 0.27 |
| XM_002491224.1 | -0.27 |
| XM_002491308.1 | 0.27 |
| XM_002490927.1 | 0.27 |
| XM_002492962.1 | -0.27 |
| XM_002492239.1 | 0.27 |
| MSTRG.735.1 | 0.27 |
| XM_002492107.1 | -0.27 |
| XM_002489342.1 | 0.27 |
| XM_002493503.1 | -0.27 |
| XM_002491438.1 | -0.26 |
| XM_002491102.1 | -0.26 |
| XM_002489338.1 | 0.26 |
| XM_002492860.1 | 0.26 |
| XM_002491774.1 | 0.26 |
| XM_002493820.1 | 0.26 |
| XM_002490777.1 | 0.26 |
| XM_002492575.1 | -0.26 |
| XM_002491321.1 | -0.26 |
| XM_002492400.1 | 0.26 |
| XM_002492082.1 | 0.26 |
| XM_002494239.1 | 0.25 |
| XM_002491795.1 | 0.25 |
| XM_002491781.1 | 0.25 |
| MSTRG.4513.1 | 0.25 |
| XM_002494323.1 | -0.25 |
| XM_002494152.1 | 0.25 |
| XM_002492104.1 | -0.25 |
| XM_002490372.1 | -0.25 |
| XM_002492039.1 | 0.25 |
| XM_002489429.1 | 0.25 |
| XM_002493744.1 | 0.24 |
| XM_002494159.1 | -0.24 |
| XM_002490729.1 | 0.24 |
| XM_002490160.1 | 0.24 |
| XM_002489933.1 | 0.24 |
| XM_002493584.1 | 0.24 |
| XM_002491848.1 | -0.24 |
| XM_002494216.1 | 0.24 |
| XM_002492662.1 | 0.24 |

**Supplementary Table 10**. Differentially expressed genes (RNA-seq), cutoff at adjusted p-value < 0.05 and |log2FC| >1, in I1S relative to GS115.

| Gene | Log_2_FC |
| --- | --- |
| Insulin | 23.37 |
| XM_002492099.1 | 13.49 |
| MSTRG.3998.5 | 12.94 |
| MSTRG.3742.2 | -12.45 |
| MSTRG.3998.13 | -12.24 |
| MSTRG.3998.6 | 12.13 |
| MSTRG.3998.7 | -11.98 |
| MSTRG.3998.11 | -11.65 |
| MSTRG.2862.3 | 11.48 |
| MSTRG.1399.5 | -11.47 |
| MSTRG.2633.24 | -11.15 |
| MSTRG.2136.4 | -9.53 |
| MSTRG.4544.1 | 8.86 |
| MSTRG.1399.1 | -7.87 |
| MSTRG.3742.5 | -7.70 |
| MSTRG.1399.2 | -7.28 |
| MSTRG.1399.3 | -7.01 |
| MSTRG.1399.4 | -6.61 |
| MSTRG.3998.14 | -6.21 |
| MSTRG.2827.2 | -5.91 |
| XM_002493617.1 | -5.22 |
| XM_002489890.1 | -5.01 |
| XM_002492687.1 | -4.98 |
| XM_002492948.1 | -4.68 |
| XM_002491313.1 | 4.45 |
| XM_002489868.1 | 4.15 |
| XM_002489408.1 | 4.00 |
| XM_002490629.1 | 3.99 |
| XM_002492686.1 | -3.93 |
| XM_002492206.1 | -3.77 |
| XM_002490229.1 | 3.51 |
| MSTRG.1399.7 | -3.44 |
| XM_002490827.1 | -3.34 |
| XM_002490674.1 | -3.33 |
| XM_002490680.1 | -3.28 |
| XM_002492182.1 | 3.27 |
| XM_002490676.1 | -3.24 |
| XM_002493440.1 | 3.18 |
| XM_002492877.1 | -3.15 |
| XM_002490001.1 | -3.09 |
| XM_002489947.1 | 2.88 |
| XM_002492354.1 | -2.79 |
| MSTRG.3742.4 | -2.75 |
| XM_002491634.1 | 2.72 |
| XM_002492332.1 | 2.71 |
| XM_002490765.1 | 2.71 |
| XM_002494336.1 | 2.58 |
| XM_002492152.1 | -2.58 |
| XM_002491026.1 | 2.55 |
| XM_002490311.1 | -2.49 |
| XM_002491358.1 | -2.45 |
| XM_002492675.1 | -2.45 |
| XM_002490320.1 | -2.41 |
| XM_002490567.1 | -2.35 |
| XM_002491035.1 | -2.35 |
| XM_002493203.1 | 2.33 |
| XM_002491521.1 | -2.33 |
| XM_002492843.1 | -2.31 |
| MSTRG.2621.1 | 2.26 |
| XM_002490877.1 | 2.24 |
| XM_002490840.1 | -2.23 |
| XM_002489983.1 | -2.13 |
| XM_002491799.1 | -2.12 |
| XM_002490496.1 | 2.11 |
| XM_002490110.1 | 2.09 |
| XM_002494291.1 | -2.04 |
| XM_002492440.1 | -2.04 |
| XM_002490494.1 | -2.02 |
| XM_002493983.1 | 2.00 |
| XM_002490475.1 | -1.99 |
| XM_002491539.1 | -1.98 |
| XM_002492351.1 | 1.95 |
| MSTRG.1239.1 | 1.95 |
| XM_002493008.1 | -1.94 |
| XM_002493251.1 | -1.89 |
| XM_002490593.1 | 1.87 |
| XM_002490661.1 | -1.85 |
| XM_002491882.1 | 1.84 |
| XM_002489699.1 | -1.81 |
| XM_002492708.1 | 1.78 |
| XM_002493052.1 | 1.75 |
| XM_002491129.1 | -1.74 |
| XM_002493033.1 | -1.73 |
| XM_002492717.1 | 1.73 |
| XM_002491027.1 | 1.73 |
| XM_002491632.1 | 1.72 |
| XM_002493752.1 | 1.70 |
| XM_002490025.1 | 1.68 |
| XM_002490769.1 | -1.68 |
| XM_002492134.1 | -1.67 |
| MSTRG.612.1 | -1.66 |
| XM_002490373.1 | -1.64 |
| XM_002492591.1 | -1.63 |
| XM_002489366.1 | 1.63 |
| MSTRG.3662.4 | -1.62 |
| XM_002493245.1 | -1.60 |
| XM_002492344.1 | -1.58 |
| XM_002491629.1 | -1.58 |
| XM_002490194.1 | 1.58 |
| XM_002490312.1 | -1.57 |
| XM_002490137.1 | 1.56 |
| XM_002491528.1 | -1.56 |
| XM_002492461.1 | -1.53 |
| MSTRG.1380.1 | -1.53 |
| XM_002493878.1 | -1.53 |
| XM_002491349.1 | -1.53 |
| XM_002491822.1 | -1.51 |
| XM_002489969.1 | -1.51 |
| XM_002490851.1 | -1.48 |
| XM_002492183.1 | 1.48 |
| XM_002491386.1 | 1.47 |
| XM_002492192.1 | -1.47 |
| XM_002489720.1 | -1.46 |
| XM_002489556.1 | 1.45 |
| MSTRG.4386.1 | 1.45 |
| XM_002490662.1 | -1.45 |
| XM_002489955.1 | -1.45 |
| XM_002489790.1 | -1.44 |
| XM_002493625.1 | -1.43 |
| XM_002493680.1 | -1.42 |
| XM_002489731.1 | -1.42 |
| MSTRG.2862.2 | 1.42 |
| XM_002491348.1 | 1.41 |
| XM_002492020.1 | 1.41 |
| MSTRG.4024.3 | 1.40 |
| XM_002490071.1 | 1.39 |
| XM_002490155.1 | -1.39 |
| XM_002493287.1 | -1.38 |
| XM_002491843.1 | -1.38 |
| XM_002493044.1 | -1.37 |
| XM_002494021.1 | -1.36 |
| MSTRG.618.1 | -1.36 |
| XM_002491260.1 | -1.36 |
| XM_002489817.1 | 1.35 |
| XM_002492340.1 | -1.35 |
| XM_002489824.1 | -1.35 |
| XM_002491385.1 | 1.35 |
| XM_002492716.1 | 1.35 |
| XM_002491881.1 | -1.34 |
| XM_002494151.1 | -1.34 |
| XM_002494235.1 | -1.33 |
| XM_002491888.1 | 1.32 |
| XM_002489994.1 | -1.32 |
| XM_002491640.1 | -1.32 |
| XM_002493885.1 | 1.32 |
| XM_002493972.1 | -1.31 |
| XM_002492408.1 | -1.31 |
| XM_002492406.1 | -1.30 |
| XM_002493441.1 | 1.30 |
| XM_002492718.1 | 1.29 |
| XM_002491006.1 | 1.29 |
| XM_002492047.1 | 1.28 |
| MSTRG.2633.6 | -1.28 |
| XM_002494294.1 | -1.28 |
| XM_002489582.1 | 1.28 |
| XM_002492828.1 | -1.28 |
| XM_002490613.1 | -1.28 |
| XM_002489718.1 | -1.27 |
| XM_002491330.1 | 1.27 |
| XM_002492287.1 | 1.27 |
| XM_002491314.1 | -1.27 |
| XM_002489422.1 | -1.27 |
| XM_002493749.1 | 1.26 |
| XM_002489807.1 | -1.25 |
| XM_002492876.1 | 1.25 |
| XM_002490628.1 | -1.25 |
| XM_002493896.1 | 1.25 |
| XM_002492021.1 | 1.24 |
| XM_002489352.1 | -1.24 |
| MSTRG.1575.1 | 1.24 |
| XM_002491883.1 | 1.23 |
| XM_002492724.1 | 1.23 |
| XM_002493551.1 | -1.23 |
| XM_002489864.1 | -1.22 |
| XM_002491768.1 | 1.22 |
| XM_002494106.1 | -1.22 |
| XM_002492749.1 | 1.22 |
| XM_002493946.1 | 1.21 |
| XM_002493991.1 | -1.20 |
| XM_002493276.1 | -1.20 |
| XM_002492018.1 | -1.20 |
| XM_002493396.1 | -1.20 |
| XM_002489781.1 | 1.19 |
| XM_002489978.1 | 1.19 |
| XM_002489589.1 | -1.19 |
| XM_002491944.1 | -1.19 |
| XM_002493083.1 | -1.18 |
| XM_002493047.1 | 1.18 |
| MSTRG.2817.1 | -1.18 |
| XM_002491989.1 | 1.18 |
| XM_002490663.1 | -1.17 |
| XM_002490572.1 | 1.17 |
| XM_002493858.1 | -1.17 |
| XM_002491821.1 | -1.17 |
| XM_002493347.1 | 1.17 |
| XM_002492163.1 | -1.17 |
| XM_002492613.1 | 1.17 |
| XM_002489613.1 | 1.17 |
| XM_002490409.1 | -1.16 |
| XM_002493293.1 | -1.16 |
| MSTRG.2203.6 | -1.16 |
| XM_002493167.1 | -1.16 |
| XM_002493470.1 | 1.15 |
| MSTRG.2644.2 | -1.14 |
| XM_002493372.1 | -1.14 |
| XM_002490231.1 | -1.14 |
| XM_002489314.1 | -1.13 |
| XM_002494058.1 | -1.13 |
| XM_002492256.1 | -1.13 |
| XM_002494240.1 | -1.13 |
| XM_002490335.1 | 1.13 |
| XM_002492164.1 | -1.12 |
| XM_002493871.1 | -1.12 |
| XM_002492180.1 | -1.11 |
| MSTRG.3551.1 | 1.11 |
| XM_002492932.1 | 1.11 |
| XM_002489669.1 | -1.11 |
| XM_002493821.1 | 1.11 |
| XM_002493663.1 | 1.11 |
| XM_002491926.1 | -1.11 |
| XM_002492352.1 | -1.10 |
| XM_002493080.1 | -1.10 |
| XM_002489964.1 | -1.10 |
| XM_002493929.1 | 1.10 |
| XM_002491206.1 | -1.09 |
| XM_002492818.1 | -1.08 |
| XM_002492516.1 | 1.08 |
| XM_002492165.1 | -1.08 |
| XM_002493569.1 | -1.08 |
| MSTRG.4408.8 | 1.08 |
| XM_002492349.1 | 1.08 |
| XM_002493733.1 | 1.07 |
| XM_002492637.1 | -1.07 |
| XM_002489811.1 | -1.07 |
| XM_002489681.1 | 1.06 |
| MSTRG.1384.4 | -1.06 |
| XM_002492685.1 | -1.06 |
| XM_002490331.1 | 1.06 |
| XM_002490522.1 | -1.06 |
| XM_002491356.1 | 1.06 |
| XM_002491707.1 | -1.06 |
| XM_002493962.1 | 1.05 |
| XM_002490306.1 | 1.05 |
| XM_002492187.1 | -1.05 |
| XM_002493011.1 | 1.05 |
| XM_002493934.1 | -1.05 |
| XM_002493368.1 | -1.05 |
| XM_002491161.1 | -1.05 |
| XM_002493500.1 | -1.05 |
| XM_002491512.1 | 1.05 |
| XM_002489946.1 | 1.05 |
| XM_002493292.1 | -1.04 |
| XM_002494020.1 | -1.04 |
| XM_002490085.1 | -1.04 |
| XM_002492902.1 | -1.04 |
| XM_002492986.1 | 1.04 |
| XM_002490577.1 | -1.04 |
| XM_002490457.1 | -1.04 |
| XM_002490751.1 | -1.04 |
| XM_002493658.1 | -1.03 |
| XM_002493904.1 | 1.03 |
| XM_002490011.1 | -1.03 |
| XM_002491351.1 | 1.03 |
| XM_002489403.1 | 1.03 |
| XM_002491793.1 | -1.03 |
| XM_002491601.1 | 1.03 |
| XM_002491876.1 | -1.02 |
| XM_002489698.1 | 1.02 |
| XM_002493156.1 | 1.02 |
| XM_002493405.1 | 1.02 |
| XM_002492230.1 | 1.02 |
| XM_002493648.1 | 1.02 |
| MSTRG.3055.1 | -1.02 |
| XM_002490285.1 | -1.01 |
| XM_002493010.1 | -1.01 |
| XM_002492714.1 | 1.01 |
| XM_002492760.1 | 1.01 |
| XM_002490185.1 | 1.01 |
| XM_002489866.1 | 1.01 |
| XM_002491394.1 | 1.01 |
| XM_002489409.1 | -1.01 |
| XM_002489396.1 | -1.01 |
| XM_002492924.1 | -1.01 |
| XM_002493525.1 | 1.01 |
| XM_002490086.1 | -1.01 |
| XM_002492926.1 | 1.00 |

**Supplementary Table 11**. Differentially expressed genes (RNA-seq), cutoff at adjusted p-value < 0.05 and |log_2_FC| >1, in I6G relative to GS115.

| Gene | Log_2_FC |
| --- | --- |
| Insulin | 22.92 |
| MSTRG.713.9 | -15.29 |
| MSTRG.3742.5 | -13.11 |
| MSTRG.2633.24 | -11.15 |
| MSTRG.4544.1 | 9.83 |
| MSTRG.2827.2 | -7.12 |
| XM_002489890.1 | -4.82 |
| MSTRG.1399.5 | -4.12 |
| XM_002490674.1 | -3.87 |
| XM_002489868.1 | 3.50 |
| MSTRG.1399.7 | -3.49 |
| XM_002490550.1 | -3.30 |
| XM_002490676.1 | -3.20 |
| XM_002493617.1 | -3.00 |
| XM_002492877.1 | -2.59 |
| XM_002493440.1 | 2.59 |
| MSTRG.3742.4 | -2.44 |
| XM_002490765.1 | 2.31 |
| XM_002490229.1 | 2.16 |
| XM_002490001.1 | -2.01 |
| XM_002491634.1 | 2.00 |
| XM_002492440.1 | -1.98 |
| XM_002492843.1 | -1.93 |
| XM_002492206.1 | -1.82 |
| XM_002492354.1 | -1.78 |
| XM_002493203.1 | 1.74 |
| XM_002492288.1 | -1.73 |
| XM_002490567.1 | -1.72 |
| XM_002491358.1 | -1.71 |
| MSTRG.2621.1 | 1.70 |
| XM_002490840.1 | -1.67 |
| XM_002492332.1 | 1.65 |
| XM_002492134.1 | -1.56 |
| XM_002493983.1 | 1.54 |
| MSTRG.1239.1 | 1.48 |
| XM_002491314.1 | -1.46 |
| XM_002491027.1 | 1.46 |
| MSTRG.4386.1 | 1.45 |
| XM_002491632.1 | 1.45 |
| XM_002490496.1 | 1.41 |
| XM_002489955.1 | -1.40 |
| XM_002490311.1 | -1.39 |
| XM_002493033.1 | -1.39 |
| XM_002492876.1 | 1.37 |
| XM_002491799.1 | -1.34 |
| XM_002494291.1 | -1.34 |
| MSTRG.2633.6 | -1.31 |
| XM_002490593.1 | 1.28 |
| XM_002492152.1 | -1.26 |
| XM_002491882.1 | 1.25 |
| XM_002493551.1 | -1.23 |
| XM_002490110.1 | 1.22 |
| XM_002489790.1 | -1.22 |
| XM_002490025.1 | 1.21 |
| XM_002493251.1 | -1.19 |
| XM_002493403.1 | 1.18 |
| XM_002492708.1 | 1.18 |
| XM_002490572.1 | 1.17 |
| XM_002493752.1 | 1.16 |
| XM_002492020.1 | 1.16 |
| XM_002489807.1 | -1.15 |
| XM_002493749.1 | 1.15 |
| XM_002493167.1 | -1.15 |
| XM_002491129.1 | -1.14 |
| XM_002492018.1 | -1.13 |
| XM_002491629.1 | -1.12 |
| XM_002492717.1 | 1.12 |
| XM_002492021.1 | 1.11 |
| MSTRG.702.1 | -1.10 |
| XM_002492351.1 | 1.09 |
| MSTRG.1380.1 | -1.08 |
| XM_002491006.1 | 1.08 |
| MSTRG.4408.8 | 1.05 |
| MSTRG.2862.2 | 1.05 |
| XM_002492256.1 | -1.04 |
| XM_002493885.1 | 1.04 |
| XM_002490194.1 | 1.04 |
| XM_002489556.1 | 1.03 |
| XM_002493625.1 | -1.03 |
| XM_002493044.1 | -1.02 |
| XM_002491260.1 | -1.02 |
| MSTRG.919.1 | -1.01 |
| XM_002490137.1 | 1.01 |
| XM_002493083.1 | -1.01 |

**Supplementary Table 12**. Differentially expressed genes (RNA-seq), cutoff at adjusted p-value < 0.05 and |log_2_FC| >1, in M6G relative to GS115.

| Gene | Log_2_FC |
| --- | --- |
| Mambalgin | 22.16 |
| MSTRG.3274.4 | -15.19 |
| MSTRG.4283.2 | -11.84 |
| MSTRG.1418.5 | -11.31 |
| MSTRG.1418.9 | -10.94 |
| MSTRG.2663.21 | -7.21 |
| XM_002493524.1 | 5.52 |
| XM_002492879.1 | -5.12 |
| XM_002492008.1 | -4.99 |
| XM_002490232.1 | -4.51 |
| XM_002489890.1 | -4.43 |
| MSTRG.3816.5 | -4.01 |
| XM_002492877.1 | -3.07 |
| MSTRG.2666.1 | 2.91 |
| XM_002490877.1 | 2.86 |
| XM_002493617.1 | -2.66 |
| XM_002490320.1 | -2.33 |
| XM_002490593.1 | 2.29 |
| XM_002490229.1 | 2.28 |
| XM_002489366.1 | 2.26 |
| XM_002489439.1 | 2.23 |
| XM_002490827.1 | -2.14 |
| XM_002492876.1 | 2.11 |
| XM_002491637.1 | 2.06 |
| XM_002491358.1 | -2.05 |
| XM_002489983.1 | -1.93 |
| XM_002492687.1 | -1.90 |
| XM_002490098.1 | 1.90 |
| XM_002490496.1 | 1.87 |
| MSTRG.2674.1 | 1.85 |
| XM_002490457.1 | -1.85 |
| XM_002491632.1 | 1.80 |
| XM_002490001.1 | -1.73 |
| XM_002490450.1 | 1.72 |
| XM_002492619.1 | 1.71 |
| XM_002492354.1 | -1.70 |
| XM_002491799.1 | -1.65 |
| XM_002492675.1 | -1.63 |
| MSTRG.3038.1 | 1.63 |
| XM_002493245.1 | -1.62 |
| XM_002491794.1 | 1.60 |
| XM_002489556.1 | 1.48 |
| MSTRG.2894.7 | 1.48 |
| XM_002489669.1 | -1.47 |
| XM_002492591.1 | -1.47 |
| XM_002490662.1 | -1.46 |
| XM_002491882.1 | 1.45 |
| XM_002493983.1 | 1.40 |
| XM_002492152.1 | -1.38 |
| XM_002490680.1 | -1.36 |
| XM_002492843.1 | -1.36 |
| XM_002493551.1 | -1.35 |
| XM_002493680.1 | -1.34 |
| XM_002489720.1 | -1.33 |
| XM_002493752.1 | 1.31 |
| XM_002493252.1 | 1.31 |
| XM_002493083.1 | -1.30 |
| XM_002493033.1 | -1.29 |
| XM_002489955.1 | -1.29 |
| XM_002494253.1 | -1.27 |
| XM_002492902.1 | -1.25 |
| XM_002493934.1 | -1.23 |
| XM_002492134.1 | -1.22 |
| XM_002493641.1 | -1.22 |
| MSTRG.4474.1 | 1.21 |
| XM_002491348.1 | 1.21 |
| XM_002491314.1 | -1.20 |
| XM_002490851.1 | -1.19 |
| MSTRG.1348.1 | -1.15 |
| XM_002490285.1 | -1.14 |
| XM_002490661.1 | -1.14 |
| XM_002490495.1 | 1.12 |
| XM_002493749.1 | 1.11 |
| MSTRG.3603.1 | 1.10 |
| XM_002493427.1 | -1.10 |
| XM_002493858.1 | -1.08 |
| XM_002491793.1 | -1.08 |
| XM_002493663.1 | 1.07 |
| MSTRG.930.2 | 1.07 |
| XM_002490139.1 | -1.06 |
| XM_002490613.1 | -1.05 |
| XM_002493167.1 | -1.05 |
| MSTRG.4599.6 | 1.05 |
| XM_002492708.1 | 1.04 |
| XM_002493500.1 | -1.04 |
| MSTRG.827.1 | 1.02 |
| XM_002493169.1 | -1.02 |
| XM_002491260.1 | -1.01 |
| XM_002491636.1 | 1.01 |
| XM_002493044.1 | -1.01 |
| XM_002491543.1 | 1.00 |

**Supplementary Table 13**. Differentially expressed intracellular proteins, cutoff at adjusted p-value < 0.05 and |log_2_FC| >1, in all insulin precursor producing strains.

| I1G-GS115 |  |
| --- | --- |
| Protein | **Log_2_FC** |
| Insulin | 9.39 |
| C4QYP6 | 3.82 |
|  |  |
| I6G-GS115 |  |
| Protein | **Log_2_FC** |
| Insulin | 12.14 |
|  |  |
| I1S-GS115 |  |
| Protein | **Log_2_FC** |
| Insulin | 13.50 |
| C4QZB8 | -1.46 |
| C4R3R2 | 1.08 |
| C4R6N2 | -2.09 |
| C4R1A5 | -2.30 |
| C4R082 | -2.53 |
| C4R8P6 | 1.41 |
| C4QYZ8 | 1.18 |
| C4QZD3 | -2.51 |
| C4R2I5 | -2.04 |
| C4QYL0 | -1.19 |
| C4R6E4 | -1.14 |
| C4R887 | 1.08 |
| C4R0H6 | 1.06 |
| C4QXI5 | 1.43 |
| C4QXC8 | 1.67 |
| C4R573 | -2.28 |
| C4R8G2 | -2.65 |
| C4QZG8 | 1.40 |
| C4QX11 | -1.50 |
| C4R2V5 | -1.02 |
| C4R3K8 | 1.27 |
| C4R0S0 | 1.12 |
| C4R746 | -1.75 |
| C4R343 | -1.43 |
| C4R3T5 | -1.49 |
| C4QYV2 | 1.16 |
| C4R046 | 1.19 |
| C4R1U4 | 1.16 |
| C4R0X7 | 1.19 |
| C4R8E3 | 1.17 |
| C4R3J4 | 1.21 |
| C4R7W5 | 1.41 |
| C4QWL1 | -1.90 |
| C4R5H0 | 1.58 |
| C4QZE2 | -1.03 |
| C4R0B7 | 1.08 |
| C4QX12 | 1.50 |
| C4R8A7 | -1.46 |
| C4R4I0 | 1.65 |
| C4QV85 | 2.00 |
| C4R3C4 | -2.39 |
| C4QW90 | -1.93 |
|  |  |
| I6G-I1G |  |
| Protein | **Log_2_FC** |
| C4QYP6 - | 3.54 |
| C4QYW7 - | 2.69 |
|  |  |
| I1S-I1G |  |
| Protein | **Log_2_FC** |
| C4QYP6 | -3.32 |
| C4QZB8 | -1.61 |
| C4R9G3 | -1.31 |
| Insulin | 4.11 |
| C4QYW7 | -2.59 |
| C4R3R2 | 1.08 |
| C4QYL0 | -1.36 |
| C4QX11 | -1.92 |
| C4R492 | 1.01 |
| C4R2I5 | -2.22 |
| C4R6N2 | -2.11 |
| C4R0A6 | -1.10 |
| C4QXI3 | 1.17 |
| C4R6T1 | 1.33 |
| C4R1A5 | -2.32 |
| C4QXM2 | -1.21 |
| C4R573 | -2.63 |
| C4QZD3 | -2.61 |
| C4R887 | 1.15 |
| C4R746 | -2.11 |
| C4R082 | -2.49 |
| C4R8P6 | 1.41 |
| C4R6E4 | -1.12 |
| C4R0S0 | 1.22 |
| C4QXI5 | 1.39 |
| C4QVC7 | 1.39 |
| C4R8D2 | -1.49 |
| C4QWQ7 | 1.22 |
| C4R0K3 | 1.03 |
| C4R2T6 | -1.19 |
| C4R3T5 | -1.60 |
| C4QXC8 | 1.59 |
| C4R6F5 | 1.08 |
| C4QVG8 | 1.07 |
| C4R3H3 | -1.32 |
| C4QW82 | -1.32 |
| C4R3G0 | 3.09 |
| C4R2V5 | -1.02 |
| C4R692 | 1.16 |
| C4R2N4 | 1.11 |
| C4R046 | 1.21 |
| C4R0X7 | 1.23 |
| C4QYD5 | -3.17 |
| C4R8G2 | -2.42 |
| C4QZE2 | -1.06 |
| C4R648 | -1.08 |
| C4R8A7 | -1.51 |
| C4QY50 | -1.97 |
| C4R3A2 | -1.01 |
| C4QW48 | 3.55 |
| C4QZ06 | -2.89 |
| C4QVG7 | 1.07 |
| C4R4H6 | -1.29 |
| C4QXA2 | 1.57 |
| C4R343 | -1.31 |
| C4R0B7 | 1.06 |
| C4R8P7 | -1.10 |
| C4QZG8 | 1.23 |
| C4R372 | -1.69 |
| C4QW90 | -1.88 |
| C4R0Z2 | -2.06 |
| C4QVC5 | -1.14 |
| C4R6B6 | 1.02 |
| C4R5H0 | 1.48 |
| C4QYW9 | 1.47 |
| C4QVL3 | 1.16 |
| C4QYK6 | 1.13 |
| C4QYV2 | 1.00 |
| C4R3K8 | 1.06 |
| C4QV85 | 1.94 |

**Supplementary Table 14**. Differentially expressed intracellular proteins, cutoff at adjusted p-value < 0.05 and |log_2_FC| >1, in all Mambalgin-1 producing strains.

| M1G-GS115 |  |
| --- | --- |
| Protein | **Log_2_FC** |
| Mambalgin-1 | 3.61 |
|  |  |
| M6G-GS115 |  |
| Protein | **Log_2_FC** |
| Mambalgin-1 | 7.77 |
| C4R4B4 | 2.10 |
| C4R088 | -1.39 |
|  |  |
| M6G-M1G |  |
| Protein | **Log_2_FC** |
| Mambalgin-1 | 4.16 |

**Supplementary Table 15**. Differentially expressed secreted proteins, cutoff at adjusted p-value < 0.05 and |log_2_FC| >1, in all Insulin precursor producing strains.

| I1G-GS115 |  |  |
| --- | --- | --- |
| Protein | **Log_2_FC** | **Signal peptide** |
| Insulin | 11.12 | Yes |
|  |  |  |
| I6G-GS115 |  |  |
| Protein | **Log_2_FC** |  |
| Insulin | 12.19 | Yes |
|  |  |  |
| I1S-GS115 |  |  |
| Protein | **Log_2_FC** |  |
| Insulin | 10.40 | Yes |
| C4QYW7 | -3.73 | Yes |
| C4R3J9 | -2.09 | No |
| C4QZ06 | -2.26 | No |
| C4R6A0 | 2.45 | No |
|  |  |  |
| I6G-I1G |  |  |
| Protein | **Log_2_FC** |  |
| C4QYW7 | -3.73 | Yes |
| C4QZD0 | -3.91 | Yes |
| C4R272 | 3.04 | No |
| C4R6P7 | 2.09 | Yes |
| C4R6M2 | 1.38 | Yes |
| C4QXC8 | 2.86 | No |
|  |  |  |
| I1S-I1G |  |  |
| Protein | **Log_2_FC** |  |
| C4QYW7 | -4.68 | Yes |
| C4QZD0 | -4.49 | Yes |
| C4R6P7 | 2.05 | Yes |
| C4QZ06 | -2.11 | No |
| C4R3J9 | -1.77 | No |
| C4R8C5 | 1.69 | No |
| C4R6A0 | 1.49 | No |
| C4QWT6 | -3.07 | No |
| C4R2I5 | -2.71 | No |
| C4R0M8 | -1.58 | No |
| C4QW42 | -1.58 | No |
| C4R2S1 | -2.05 | No |
| C4R8G2 | -2.14 | No |
| C4R3R2 | 1.38 | No |
| C4QZ90 | -1.74 | No |
| C4R3C4 | -1.74 | Yes |
| C4R489 | -3.14 | Yes |
| C4QZC2 | 1.44 | Yes |
| C4R5T4 | -3.50 | No |
| C4QXY3 | 1.14 | No |
| C4R7G7 | -1.04 | Yes |
| C4R0X7 | 1.07 | No |
| C4R862 | -1.30 | No |

**Supplementary Table 16**. Differentially expressed secreted proteins, cutoff at adjusted p-value < 0.05 and |log_2_FC| >1, in all Mambalgin-1 producing strains.

| M1G-GS115 |  |  |
| --- | --- | --- |
| Protein | **Log_2_FC** | **Signal peptide** |
| Mambalgin-1 | 9.45 | Yes |
|  |  |  |
| M6G-GS115 |  |  |
| Protein | **Log_2_FC** |  |
| Mambalgin-1 | 11.23 | Yes |
|  |  |  |
| M6G-M1G |  |  |
| Protein | **Log_2_FC** |  |
| C4R146 | -1.26 | No |
| C4QV80 | -1.65 | No |
| C4R0T7 | -1.00 | No |
| C4QYE9 | -2.07 | No |
| Mambalgin-1 | 1.78 | Yes |
| C4QXC0 | -1.70 | No |

## Bibliography

Kastberg, L. L. B., Petrov, M. S., Strucko, T., Jensen, M. K., & Workman, C. T. (2024). Codon-tRNA Coadaptation Bias for Identifying Strong Native Promoters in Komagataella phaffii. *ACS Synthetic Biology*, *13*(3), 714–720. https://doi.org/10.1021/acssynbio.3c00567

Strucko, T., Gadar-Lopez, A.-E., Frøhling, F. B., Frost, E. T., Iversen, E. F., Olsson, H., Jarczynska, Z. D., & Mortensen, U. H. (2024). Oligonucleotide-based CRISPR-Cas9 toolbox for efficient engineering of Komagataella phaffii. *FEMS Yeast Research*, foae026. https://doi.org/10.1093/femsyr/foae026
